# A New Approach to the Nonparametric Behrens–Fisher Problem With Compatible Confidence Intervals

**DOI:** 10.1002/bimj.70096

**Published:** 2025-11-09

**Authors:** Stephen Schüürhuis, Frank Konietschke, Edgar Brunner

**Affiliations:** ^1^ Institute of Biometry and Clinical Epidemiology Charit‐Universitätsmedizin Berlin Freie Universität Berlin and Humboldt‐Universität zu Berlin Berlin Germany; ^2^ Department of Medical Statistics Universitätsmedizin Göttingen Göttingen Germany

**Keywords:** Birnbaum–Klose inequality, Brunner–Munzel test, Mann–Whitney effect θ, studentized permutation test, $C^{2}$‐test

## Abstract

We propose a new method to address the nonparametric Behrens–Fisher problem, allowing for unequal distribution functions across the two samples. The procedure tests the null hypothesis $H_{0}:\theta =1/2$, where θ=P(X<Y)+1/2P(X=Y) denotes the Mann–Whitney effect. Apart from the trivial case of one‐point distributions, no restrictions are imposed on the underlying data distribution. The test is derived by evaluating the ratio of the true variance $\sigma_{N}^{2}$ of the Mann–Whitney effect estimator ${\hat{\theta }}_{N}$ to its theoretical maximum, as derived from the Birnbaum–Klose inequality. Through simulations, we demonstrate that the proposed test effectively controls the type‐I error rate under various conditions, including small and unbalanced sample sizes, and different data‐generating mechanisms. Notably, it provides better control of the type‐I error rate than the widely used Brunner–Munzel test, particularly at small significance levels such as α=0.005. We further construct range‐preserving compatible confidence intervals and show that they exhibit improved coverage compared to the confidence intervals compatible to the Brunner–Munzel test. Finally, we illustrate the application of the method in a clinical trial example.

## 1 Introduction

Comparing the means (expectations) of two independent groups with potentially different distributions (variances) is a fundamental problem in statistics, commonly encountered in fields such as medicine, biology, psychology, social sciences, and education. When the assumption of equal variances between groups is questionable in case of underlying normal distributions, the classical *t*‐test may no longer be appropriate, leading to the well‐known parametric Behrens–Fisher problem. However, the assumption of normality itself may not hold in many practical data analysis scenarios, as the underlying distributions from which the data are sampled can be skewed or exhibit heavy tails. Moreover, the assumption of the normal distribution is inappropriate for ordinal data such as data observed on Likert scales. In such cases, means do not offer a meaningful definition of a treatment effect. Instead, it appears more appropriate to frame the problem as the *nonparametric* Behrens–Fisher problem of testing

$$\begin{array}{c} H_{0}:\theta =P\left(X_{1}<X_{2}\right)+1/2P\left(X_{1}=X_{2}\right)=1/2, \end{array}$$

where θ represents the Mann–Whitney effect, a measure that offers a meaningful assessment of treatment effects, regardless of the underlying data distribution.

Many authors have developed methods for the nonparametric Behrens–Fisher problem, offering robust alternatives that relax the restrictive assumptions of parametric approaches (see, e.g., Fligner and Policello, 1981; Brunner and Munzel, 2000; Neubert and Brunner, 2007; Pauly et al., 2016). These nonparametric methods are particularly valuable in settings like preclinical phases or rare diseases, where accurate comparisons between treatment groups with limited sample sizes are essential. Among various procedures, the test proposed by Brunner and Munzel (2000) is widely recommended for comparing two heteroscedastic samples due to its accurate control of the type‐I error rate for α≥0.05 and sufficiently large‐sample sizes (Karch, 2021; Wilcox, 2003). However, the Brunner–Munzel test has two main weaknesses: (1) it behaves liberally and over‐rejects the null hypothesis at small‐sample sizes and/or low‐significance levels and (2) its compatible confidence intervals are not range‐preserving and show poor coverage for large effects θ (Noguchi et al., 2021; Pauly et al., 2016). Those properties are particularly noteworthy given recent developments advocating for the adoption of more stringent significance levels (see, e.g., Johnson, 2013; Benjamin et al., 2018; Held, 2019). Moreover, in the setting of multiple hypothesis testing, standard correction methods often produce adjusted α‐levels that fall well below the conventional threshold of α=0.05. Accordingly, it is the goal of this paper to address the limitations of the Brunner–Munzel test in detail, proposing a new closed‐form procedure that improves performance also at small significance levels and provides range‐preserving compatible confidence intervals.

Previous approaches addressing the nonparametric Behrens–Fisher problem include the tests by Fligner and Policello (1981) which is valid only for continuous distributions and Funatogawa and Funatogawa (2023), who developed a testing procedure for ordered categorical data. In contrast, the Brunner–Munzel test imposes no restrictions on the underlying distributions (with the trivial exception of one‐point distributions) and is often regarded as the preferred method (Karch, 2021; Wilcox, 2003), underpinned by being implemented in various software packages, such as brunnermunzel (Ara 2022), lawstat (Gastwirth et al. 2023), or rankFD by Konietschke and Brunner (2023). In their paper, they propose approximating the distribution of the test statistic using a Satterthwaite–Welch–Smith t‐approximation (Satterthwaite, 1946; Welch, 1938; Smith, 1936), which works well for sufficiently large‐sample sizes but may prove inadequate for small‐sample sizes and low‐significance levels. In response, Neubert and Brunner (2007) proposed approximating the distribution of the test statistic using a studentized permutation approach, demonstrating improved type‐I error rate control for small‐sample sizes and low‐significance levels (see Pauly et al., 2016; Noguchi et al., 2021). However, permutation tests rely on resampling for p‐value computation, which can be computationally intensive and limit their practicality in certain applications. Additionally, the associated confidence intervals are not necessarily range‐preserving, requiring range‐preserving transformations, such as the logit‐tranformation as proposed by Pepe (2003) and also discussed by Kaufmann et al. (2005) and Pauly et al. (2016). Yet, it seems reasonable to conclude that the Brunner–Munzel test, as a closed‐form procedure, and the studentized permutation test, as a resampling method, currently serve as benchmark approaches for addressing the nonparametric Behrens–Fisher problem.

Conceptually, the Brunner–Munzel test and the corresponding studentized permutation test are similar, as both seek to correct for liberal tendencies by approximating the distribution of the test statistic: the former uses a t‐approximation, while the latter employs a studentized permutation distribution. For both tests, the need for approximations partly arises from using the DeLong et al. (1988) variance estimator, which may be biased in both directions, with a positive bias in the case of continuous distributions (Brunner and Konietschke 2025). Moreover, the t‐approximation of the Brunner–Munzel test statistic is theoretically arguable, as the numerator, which includes the asymptotically normal effect estimator, and the denominator, which contains the DeLong variance estimator, are not stochastically independent.

Here, we approach the nonparametric Behrens–Fisher problem from a different perspective: rather than proposing a new approximation of the distribution of the test statistic, we derive a new test by considering the ratio of the variance of the Mann–Whitney effect estimator, $\sigma_{N}^{2}$, to its theoretical upper bound as derived from the Birnbaum–Klose inequality (Birnbaum and Klose 1957). Unlike existing approaches relying on the variance estimator by DeLong et al. (1988), we employ the unbiased rank‐based variance estimator introduced by Brunner and Konietschke (2025). Additionally, we provide compatible confidence intervals for the new method and show that they are range‐preserving. To narrow the focus of our study, we concentrate on a test with a closed‐form solution, excluding resampling‐based methods in the present paper. Using simulation, we illustrate that the new test procedure has an improved type‐I error rate control, while maintaining power comparable to the Brunner–Munzel and studentized permutation tests. Furthermore, we compare the coverage probability of the associated confidence intervals, illustrating that the new confidence intervals provide satisfactory coverage for an increased range of true effects θ as compared to the confidence intervals obtained by inverting the Brunner–Munzel test.

This paper is organized as follows. Section 2 presents a motivating data example. The statistical model, estimators, and methods under consideration are detailed in Sections 3 and 4. A comprehensive simulation study targeting the type‐I error rate and power of the tests, as well as the coverage probability of the compatible confidence intervals, is presented in Section 5. The data example is revisited in Section 6. Finally, the paper closes with a discussion and an outlook on potential developments. Proofs and more detailed derivations are deferred to the Appendix.

## 2 Data Example

To illustrate the application of all the methods under consideration, we reanalyze the data from a shoulder tip pain study conducted by Jorgensen et al. (1995) and later reported by Lumley (1996), which was also used in the papers by Brunner and Munzel (2000) and Neubert and Brunner (2007). This randomized controlled trial introduces a specific suction procedure to remove air from the abdomen after a laparoscopic surgery as a new intervention. A total of n=22 patients are randomized to the treatment group, while n=19 patients are in the control group. Pain is assessed using a score ranging from 1 (no pain) to 5 (severe pain) on the evening of the second‐day post‐surgery. That is, this trial's primary endpoint has an ordinal measurement level. For details on the procedure and the trial, we refer to Lumley (1996) and the original trial by Jorgensen et al. (1995). Table 1 summarizes the trial data utilizing absolute frequencies of the different pain score values. The boxplot illustrates the empirical distribution of the data demonstrating that the pain score is highly skewed in the intervention group.

**TABLE 1:** Pain scores on the evening of Day 2 after laparoscopic surgery: comparison between 22 patients with specific suction procedure and 19 control patients.

	Pain score					
Treatment arm	1	2	3	4	5	Total
Specific suction	16	5	0	1	0	22
Control	4	1	5	7	2	19


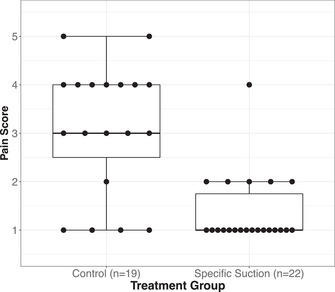


## 3 Notation and Nonparametric Model

### 3.1 The Mann–Whitney Effect

We consider the nonparametric model stated by Brunner and Munzel (2000) and consider independent random variables $X_{ik}∼F_{i},\;k=1,\cdots ,n_{i}$, where i=1,2 denotes the treatment arms and $N=n_{1}+n_{2}$ the total sample size. Note that the distributions $F_{i}$ may be arbitrary, excluding the case of one‐point distributions. The estimand most naturally associated with the nonparametric Behrens–Fisher problem is the Mann–Whitney effect

(1) $$\begin{array}{ccc} \theta & = & \int F_{1}\;dF_{2}=P\left(X_{11}<X_{21}\right)+1/2P\left(X_{11}=X_{21}\right), \end{array}$$

where $F_{i}=\left(F_{i}^{+}+F_{i}^{-}\right)/2$ represents the normalized version of the right and left continuous cumulative distribution functions (Ruymgaart 1980). Note that this definition provides a unified notation that covers continuous and discrete data. In practice, θ can be interpreted as the probability that a random observation drawn from $F_{2}$ will be larger than a random observation drawn from $F_{1}$ while accounting for the probability of ties. That is, if θ>(<)0.5, $X_{11}$ is said to tend to smaller (larger) values than $X_{21}$. If θ=0.5, $X_{11}$ and $X_{21}$ are said to be stochastically comparable. Correspondingly, the nonparametric Behrens–Fisher problem can be formulated in terms of the null hypothesis $H_{0}:\theta =1/2$, representing the two‐sided hypothesis of no tendency toward larger values in either population. The commonly used estimator ${\hat{\theta }}_{N}$ of θ can be derived by replacing the theoretical distribution functions $F_{i}$ by their empirical counterparts ${\hat{F}}_{i}$, resulting in

(2) $$\begin{array}{ccc} {\hat{\theta }}_{N} & = & \int {\hat{F}}_{1}d{\hat{F}}_{2}=\frac{1}{n_{1}}\left({\bar{R}}_{2\cdot }-\frac{n_{2}+1}{2}\right), \end{array}$$

where ${\bar{R}}_{2\cdot }=\frac{1}{n_{2}}\sum_{i=1}^{n_{2}}R_{ik}$ is the mean of all ranks $R_{ik}$ and $R_{ik}$ is the rank of observation $X_{ik}$ among all i=1,⋯,N observations, see Brunner et al. (2019) (Section 3.3, Result 3.1) for details. For asymptotic results, we assume that $N/n_{i}$ is uniformly bounded, that is, $N/n_{i}\leq N_{0}<\infty$, compare Brunner et al. (2019) for details (Section 3, Assumptions 3.17 and 3.19).

The construction of tests for $H_{0}:\theta =1/2$ requires knowledge about the variance $\sigma_{N}^{2}\;=\;Var\left({\hat{\theta }}_{N}\right)$ or $\gamma_{N}^{2}\;=\;Var\left(\sqrt{N}\;{\hat{\theta }}_{N}\right)=N\sigma_{N}^{2}$ of the estimator ${\hat{\theta }}_{N}$. Many papers address the estimation of $\sigma_{N}^{2}$. For example, Fligner and Policello (1981) proposed an estimator valid only for continuous distribution functions. In contrast, Bamber (1975) provided an unbiased estimator that remains valid in the presence of ties but did not consider a rank‐based representation of the estimator (for details, we refer to Brunner and Konietschke 2025). The following section introduces the variance estimators used for the tests in this paper. Specifically, we present the estimator proposed by DeLong et al. (1988), which is used in the Brunner–Munzel test, as well as the rank‐based representation of the Bamber (1975) estimator provided by Brunner and Konietschke (2025), which is used in our proposed test. It is worth noting that alternative variance estimators, including the Perme–Manevski estimator (Perme and Manevski 2019) and the Hanley–McNeil estimator (Hanley and McNeil 1982), are available and have been discussed in detail by Brunner and Konietschke (2025).

### 3.2 Variance Estimation

#### 3.2.1 Estimator by DeLong et al. (1988)

For the derivation of this variance estimator, Brunner and Munzel (2000) used the asymptotic variance

$$\begin{array}{ccc} v_{N}^{2} & = & N\left(\frac{\sigma_{1}^{2}}{n_{1}}+\frac{\sigma_{2}^{2}}{n_{2}}\right) \end{array}$$

  based on the asymptotic equivalence theorem, as discussed in Brunner et al. (2019) (Section 7, Theorem 7.16) or Brunner and Munzel (2000). Here, $\sigma_{1}^{2}=Var\left(F_{2}\left(X_{1i}\right)\right)$ and $\sigma_{2}^{2}=Var\left(F_{1}\left(X_{2j}\right)\right)$ are generally unknown and need to be estimated from the data. With $R_{ik}^{\left(i\right)}$ denoting the internal rank of observation $X_{ik}$ within sample i and

(3) $$\begin{array}{ccc} R_{ik}^{*} & = & R_{ik}-R_{ik}^{\left(i\right)} \end{array}$$

the so‐called placement of $X_{ik}$ in the respective other sample $i^{\prime }\neq i$ (Orban and Wolfe 1982), replacing $F_{i}$ with the corresponding empirical distribution function ${\hat{F}}_{i}$ leads to the $L_{2}$‐consistent estimator

(4) $$\begin{array}{ccc} {\hat{\sigma }}_{i}^{2} & = & \frac{1}{{\left(N-n_{i}\right)}^{2}\left(n_{i}-1\right)}\sum_{k=1}^{n_{i}}{\left(R_{ik}^{*}-\;{\bar{R}}_{i\cdot }+\frac{n_{i}+1}{2}\right)}^{2} \end{array}$$

of $\sigma_{i}^{2}$. The corresponding estimator for $v_{N}^{2}$ is obtained by the plug‐in estimator

(5) $$\begin{array}{ccc} {\hat{v}}_{\text{DL}}^{2} & = & N\left(\frac{{\hat{\sigma }}_{1}^{2}}{n_{1}}+\frac{{\hat{\sigma }}_{2}^{2}}{n_{2}}\right). \end{array}$$

Since the estimator provided by Brunner and Munzel (2000) is identical to that of DeLong et al. (1988), we refer to it as the DeLong estimator for the remainder of the paper. Note that this estimator is positively biased for continuous underlying distributions, as discussed by Brunner and Konietschke (2025).

#### 3.2.2 Estimator by Brunner and Konietschke (2025)

The true variance $\sigma_{N}^{2}$ of the Mann–Whitney estimator ${\hat{\theta }}_{N}$ in Equation (2) can be expressed as

$$\begin{array}{c} \sigma_{N}^{2}=\frac{1}{n_{1}n_{2}}\left[\left(n_{1}-1\right)\sigma_{2}^{2}+\left(n_{2}-1\right)\sigma_{1}^{2}+\theta \left(1-\theta \right)-\frac{\tau }{4}\right], \end{array}$$

where again $\sigma_{1}^{2}=Var\left(F_{2}\left(X_{1i}\right)\right)$ and $\sigma_{2}^{2}=Var\left(F_{1}\left(X_{2j}\right)\right)$ and τ denotes the probability of ties in the overlap region of $F_{1}$ and $F_{2}$. This representation of $\sigma_{N}^{2}$ can be found already in Bamber (1975), where an unbiased estimator of $\sigma_{N}^{2}$ based on different U‐statistics is suggested without further discussion. In a recent paper, Brunner and Konietschke (2025) introduced a rank‐based version of Bamber's variance estimator by considering the U‐statistic representation of ${\hat{\theta }}_{N}$ instead of the rank‐based form in Equation (2). They derived the following unbiased rank‐based estimator

(6) $$\begin{array}{c} {\hat{\sigma }}_{N}^{2}=\frac{1}{d_{N}}\left[\sum_{i=1}^{2}\sum_{k=1}^{n_{i}}{\left(R_{ik}^{*}-{\bar{R}}_{i\cdot }^{*}\right)}^{2}-n_{1}n_{2}\left({\hat{\theta }}_{N}\left(1-{\hat{\theta }}_{N}\right)-\frac{{\hat{\tau }}_{N}}{4}\right)\right], \end{array}$$

where $d_{N}=n_{1}\left(n_{1}-1\right)n_{2}\left(n_{2}-1\right)$ and ${\bar{R}}_{i\cdot }^{*}=\frac{1}{n_{1}}\sum_{i=1}^{n_{i}}R_{ik}^{*}$ represents the mean of the placements $R_{ik}^{*}$ defined in Equation (3). The probability of ties in the overlap region of $F_{1}$ and $F_{2}$, namely $\tau =P(X_{1j}=X_{2i})$, can be estimated by

(7) $$\begin{array}{ccc} {\hat{\tau }}_{N} & = & \frac{1}{n_{1}}\left[{\bar{R}}_{2\cdot }^{\;+}-{\bar{R}}_{2\cdot }^{\;-}-\left({\bar{R}}_{2\cdot }^{(2)+}-{\bar{R}}_{2\cdot }^{(2)-}\right)\right] \end{array}$$

where ${\bar{R}}_{2\cdot }^{+}$ and ${\bar{R}}_{2\cdot }^{+}$ represent the means of the maximum and minimum ranks $R_{2k}^{+}$ and $R_{2k}^{-}$ among all N observations and ${\bar{R}}_{2\cdot }^{(2)+}$ and ${\bar{R}}_{2\cdot }^{(2)-}$ denote the means of the maximum and minimum internal ranks $R_{2k}^{(2)+}$ and $R_{2k}^{(2)-}$ among all $n_{2}$ observations in the second sample, respectively. For details, see Brunner and Konietschke (2025).

In contrast to the DeLong estimator ${\hat{v}}_{\text{DL}}^{2}$, this estimator is unbiased and non‐negative for all sample sizes $n_{i}\geq 2$. Moreover, Brunner and Konietschke (2025) showed that ${\hat{\sigma }}_{N}^{2}$ is bounded by ${\hat{\theta }}_{N}\left(1-{\hat{\theta }}_{N}\right)/\left(m-1\right)$, where $m=\min\{n_{1},n_{2}\}$. This sharp upper bound can be considered an empirical version of the Birnbaum–Klose inequality (1957). Given these preferable properties, we will use this variance estimator in the new test procedure.

## 4 Tests for the Nonparametric Behrens–Fisher Problem

### 4.1 The Brunner–Munzel–Test (2000)

To derive tests for the nonparametric Behrens–Fisher Problem $H_{0}:\theta =1/2$, first consider the quantity $T_{N}=\sqrt{N}\left({\hat{\theta }}_{N}-1/2\right)/\gamma_{N}$, where $\gamma_{N}^{2}=Var\left(\sqrt{N}\;{\hat{\theta }}_{N}\right)$ and note that, asymptotically, $T_{N}$ has a standard normal distribution, i.e., $P(|T_{N}|>z_{1-\alpha /2})=\alpha$ for N→∞ if $\gamma_{N}>0$ and $N/n_{i}\leq N_{0}<\infty$, i=1,2. Let ${\hat{\gamma }}_{N}^{2}$ denote some consistent estimator of $\gamma_{N}^{2}$, then by Slutsky's theorem,

(8) $$\begin{array}{ccc} P\left(|\sqrt{N}\left({\hat{\theta }}_{N}-1/2\right)/{\hat{\gamma }}_{N}|>z_{1-\alpha /2}\right) & = & \alpha \;\text{as}\;N\to \infty \;. \end{array}$$

In particular, if ${\hat{\gamma }}_{N}^{2}={\hat{v}}_{\text{DL}}^{2}$ as given in Equation (5), then, under $H_{0}$,

(9) $$\begin{array}{ccc} T_{N}^{\text{BM}} & = & \sqrt{N}\left({\hat{\theta }}_{N}-1/2\right)/\;{\hat{v}}_{DL}\;\overset{\;d\;}{\to }\;N\left(0,1\right) \end{array}$$

is the test statistic of the Brunner–Munzel test, which, asymptotically, follows a standard normal distribution. Simulation studies have demonstrated that large‐sample sizes are required for a satisfactory approximation. In many medical and biological applications, however, the number of available observations may be quite small. Consequently, Brunner and Munzel (2000) proposed to approximate the distribution of $T_{N}^{\text{BM}}$ by a $t_{f}$‐distribution, where the degrees of freedom are estimated through

(10) $$\begin{array}{c} \hat{f}=\frac{{\left[\sum_{i=1}^{2}{\hat{\sigma }}_{i}^{2}/\left(N-n_{i}\right)\right]}^{2}}{\sum_{i=1}^{2}{\left\{{\hat{\sigma }}_{i}^{2}/\left(N-n_{i}\right)\right\}}^{2}/\left(n_{i}-1\right)}\;, \end{array}$$

and ${\hat{\sigma }}_{i}^{2}$, i=1,2, is given in Equation (4). The estimated degrees of freedom $\hat{f}$ are obtained by replacing the variance estimators in the parametric Satterthwaite–Smith–Welch approximation by ${\hat{\sigma }}_{i}^{2}$ in Equation (4). Finally, the hypothesis $H_{0}:\theta =1/2$ is rejected at a two‐sided significance level α if

(11) $$\begin{array}{c} |T_{N}^{\text{BM}}|>t_{\hat{f},1-\alpha /2}\;, \end{array}$$

where $t_{\hat{f},1-\alpha /2}$ denotes the 1−α/2‐quantile of a central t‐distribution with $\hat{f}$ degrees of freedom.

**FIGURE 1:**
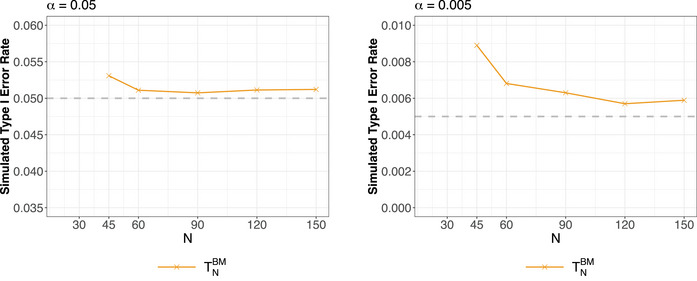
Simulated type‐I error rate of the Brunner–Munzel test $T_{N}^{\text{BM}}$ for normal distributions $F_{1}=N\left(0,1\right)$ and $F_{2}=N\left(0,9\right)$ based on 100,000 replications at a two‐sided nominal significance level for α=0.05 (left) and α=0.005 (right). The sample sizes are chosen such that $n_{1}=2n_{2}$, resulting in a so‐called negative pairing of the variances and sample sizes. The dashed gray line represents the nominal significance level.

Exemplary results of the type‐I error rate of the test are presented in Figure 1, considering the case of heteroscedastic normal samples $F_{1}=N\left(0,1\right)$ and $F_{2}=N\left(0,9\right)$ with sample sizes $n_{1}=2n_{2}$, such that the larger variance corresponds to the smaller group. The results demonstrate that the Brunner–Munzel test may become slightly liberal for small‐sample sizes at a significance level of α=0.05. Notably, the test exhibits a clear liberal tendency at a lower α‐level of 0.5%, consistent with the findings of Noguchi et al. (2021). This behavior suggests that the t‐approximation may be inadequate in certain scenarios and motivates the exploration of alternative methods.

The 100(1−α)%‐confidence interval most naturally associated with this test can be obtained by inverting the test in Equation (11) leading to the confidence interval

(12) $$\begin{array}{c} \theta_{L,U}^{\text{BM}}={\hat{\theta }}_{N}\mp t_{\hat{f},1-\alpha /2}\frac{{\hat{v}}_{\text{DL}}}{\sqrt{N}}. \end{array}$$

Although this confidence interval is, by construction, compatible to the test decision from the test in Equation (11), it suffers from a poor coverage probability for larger effects θ (Pauly et al. 2016). Additionally, the confidence interval is not range‐preserving. To illustrate this drawback, consider the artificial dataset in Table 2. Using these data, we obtain a Mann–Whitney effect estimate of ${\hat{\theta }}_{N}=0.8$ and a p‐value of 0.0239, indicating the rejection of $H_{0}:\theta =1/2$ at a two‐sided significance level of α=0.05. The corresponding compatible 95%‐confidence interval calculated based on Equation (12) is [0.55,1.05], demonstrating a non‐range preserving confidence interval since 1.05>1. Alternatively, a range‐preserving logit‐transformation based confidence interval (see, e.g., Pauly et al., 2016; Kaufmann et al., 2005; Pepe, 2003) could be used.

**TABLE 2:** Example dataset to illustrate the use of the confidence interval in Equation (12).

Groups	Outcome
Group 1	1956, 3828, 2051, 3721, 3233, 2000, 4000, 4428, 2603, 2370
Group 2	820, 3364, 1957, 1851, 2984, 744, 2044

This, however, is not compatible with the decision of the Brunner–Munzel test, which can be shown by simple counterexamples.

### 4.2 The Neubert and Brunner (2007) Test

To address the liberal behavior of the Brunner–Munzel test for small‐sample sizes and α∈{0.005,0.01}, Neubert and Brunner (2007) proposed a studentized permutation test, see also Pauly et al. (2016). The first step in conducting the test is to calculate the test statistic $T_{N}^{\text{BM}}$ from Equation (9). Afterward, their approach involves collecting all data into a vector

$$\begin{array}{c} X=(X_{11},\cdots ,X_{1n_{1}},X_{21},\cdots ,X_{2n_{2}}), \end{array}$$

randomly permute the observed data $n_{p}$ times and compute the Brunner–Munzel test statistic $T_{N,h}^{\text{BM}}$ for each permuted dataset. All test statistics are stored in a vector $T_{\mathrm{perm}}^{\mathrm{BM}}=\left(T_{N,1}^{\text{BM}},\text{…},T_{N,n_{p}}^{\text{BM}}\right)$ and the two‐sided p‐value is computed as

(13) $$\begin{array}{c} p\text{-value}=2\cdot \min\left\{\frac{1}{n_{p}}\sum_{h=1}^{n_{p}}1_{\left\{T_{N,h}^{\text{BM}}\leq T_{N}^{\text{BM}}\right\}},\frac{1}{n_{p}}\sum_{h=1}^{n_{p}}1_{\left\{T_{N,h}^{\text{BM}}\geq T_{N}^{\text{BM}}\right\}}\right\}. \end{array}$$

Finally, the null hypothesis is rejected if the p‐value obtained from the empirical permutation distribution of $T_{\mathrm{perm}}^{\mathrm{BM}}$ is smaller than the nominal significance level of α.

Remarks 4.1 Note that this computation may yield a p‐value of 0, even though the actual value cannot be zero considering the full permutation space. An alternative computation strategy is to include the identity permutation, resulting in $\left(U+1\right)/\left(n_{p}+1\right)$, where U denotes one of the sums in Equation (13) (Phipson and Smyth, 2010; Hemerik and Goeman, 2018). For a large number of permutations $n_{p}$, however, the difference between the two methods becomes negligible.

Based on the approximation of the test statistic using the permutation distribution, a 100(1−α)%‐confidence interval can be constructed as

(14) $$\begin{array}{c} \theta_{L,U}^{\text{perm}}=\left[{\hat{\theta }}_{N}-c_{1}\frac{{\hat{v}}_{\text{DL}}}{\sqrt{N}},{\hat{\theta }}_{N}-c_{2}\frac{{\hat{v}}_{\text{DL}}}{\sqrt{N}}\right], \end{array}$$

where $c_{1}$ and $c_{2}$ denote the empirical 1−α/2 and α/2‐quantile of $T_{\mathrm{perm}}^{\mathrm{BM}}$.

Pauly et al. (2016) provided a rigorous proof stating that the conditional permutation distribution of the studentized test statistics is approximately standard normal for any value of θ, justifying the construction of compatible confidence intervals. By simulation, Neubert and Brunner (2007) already demonstrated that this test adequately controls the type‐I error rate especially in small‐sample sizes, indicating that the distribution of the permuted test statistics approximates the null distribution of $T_{N}^{\text{BM}}$ quite well. Noguchi et al. (2021) illustrated that the studentized permutation test robustly controls the type‐I error rate also for a nominal significance level as small as α=0.005. Therefore, we include this test in this paper for comparison. This test is a well‐known alternative to the standard Brunner–Munzel test, referring to it as studentized permutation test in the following.

Since the test relies on iteratively permuting the data and re‐computing the ranks and the statistic for each permutation, it tends to be computationally intensive. While this is not an issue when analyzing a single dataset, simulation‐based power evaluations and trial designs may involve quite long run times. In this context, analytical solutions would offer an advantage. Moreover, the corresponding confidence interval tends to be liberal for larger effects, as noted by Pauly et al. (2016).

Based on the aforementioned limitations, it is our intention to develop a novel analytical test procedure that remains valid even at smaller nominal significance levels, such as 0.001≤α≤0.05. The motivation for focusing on smaller nominal α‐levels stems from recent recommendations advocating for more stringent significance thresholds, such as 0.5% (see, e.g., Johnson, 2013; Benjamin et al., 2018; Held, 2019). Furthermore, in the context of multiple testing, commonly used correction procedures often yield adjusted α‐levels that are substantially smaller than the conventional threshold of α=0.05. A second key requirement for the new test procedure is the availability of confidence intervals for the Mann–Whitney effect θ that are compatible with the test decision and range‐preserving, unlike those derived from directly inverting the Brunner–Munzel test—as illustrated in Table 2. Additionally, the proposed procedure must remain well‐defined and stable even in extreme cases, such as complete sample separation, where the estimated variance ${\hat{\sigma }}_{N}^{2}=0$.

In the next section, we will introduce a new test procedure that follows a different strategy, rather than merely replacing the unknown variance $\sigma_{N}^{2}$ of ${\hat{\theta }}_{N}$ with a consistent estimator and then providing an approximation of the asymptotic normal distribution. The test will then be compared to the Brunner–Munzel test and the corresponding studentized permutation test by means of a simulation study.

### 4.3 A New Test for $H_{0}:\theta =1/2$

To derive the proposed test of the hypothesis $H_{0}:\theta =1/2$, we again start by noting that for large‐sample sizes,

(15) $$\begin{array}{ccc} P\left(|{\hat{\theta }}_{N}-\theta |/\sigma_{N}>z_{1-\alpha /2}\right) & = & \alpha \end{array}$$

since ${\hat{\theta }}_{N}$ is asymptotically normally distributed. Various options to approximate the unknown variance $\sigma_{N}^{2}$ in Equation (15) are available. The most straightforward solution directly replaces $\sigma_{N}^{2}$ with a suitable consistent variance estimator. For instance, replacing $\sigma_{N}^{2}$ with the estimator ${\hat{v}}_{\text{DL}}^{2}$ in Equation (5), one obtains the Brunner–Munzel statistic as presented earlier. Alternatively, one could replace the unknown variance $\sigma_{N}^{2}$ by the theoretical upper bound of $\sigma_{N}^{2}$ which is given by $\sigma_{N,\max}^{2}=\theta \left(1-\theta \right)/m$ according to the Birnbaum–Klose inequality (Birnbaum and Klose 1957). Here, $m=\min\{n_{1},n_{2}\}$ denotes the smaller sample size in both groups. By using the theoretical upper bound of the variance, this approach does not require estimating the variance and the resulting test will, unsurprisingly, be quite conservative.

The key idea of the new approach is to estimate the ratio between the true variance $\sigma_{N}^{2}$ and its maximum value $\sigma_{N,\max}^{2}$ by an appropriate estimator. Here, we simply estimate both the numerator and the denominator of

(16) $$\begin{array}{ccc} r & = & \frac{\sigma_{N,\max}^{2}}{\sigma_{N}^{2}}\;=\;\frac{\theta (1-\theta )}{m\;\sigma_{N}^{2}} \end{array}$$

by plugging in the estimators ${\hat{\theta }}_{N}$ and ${\hat{\sigma }}_{N}^{2}$ from Equations (2) and (6) and we obtain

(17) $$\begin{array}{ccc} \hat{r} & = & \frac{{\hat{\theta }}_{N}\left(1-{\hat{\theta }}_{N}\right)}{m\;{\hat{\sigma }}_{N}^{2}}. \end{array}$$

By approximating r with $\hat{r}$, the true variance $\sigma_{N}^{2}$ can be expressed as a quadratic function of θ and of the quantity

(18) $$\begin{array}{ccc} \hat{q} & = & \frac{{\hat{\sigma }}_{N}^{2}}{{\hat{\theta }}_{N}\left(1-{\hat{\theta }}_{N}\right)}\;, \end{array}$$

which can be computed from the data. More concretely, setting $r\approx \hat{r}$ yields the following approximation of the true variance:

$$\begin{array}{ccc} \sigma_{N}^{2} & \approx & \frac{{\hat{\sigma }}_{N}^{2}}{{\hat{\theta }}_{N}\left(1-{\hat{\theta }}_{N}\right)}\;\theta \left(1-\theta \right)\;=\;\hat{q}\;\theta \left(1-\theta \right)\;. \end{array}$$

  Inserting this relation in Equation (15), one obtains

(19) $$\begin{array}{ccc} P\left(|{\hat{\theta }}_{N}-\theta |/\sigma_{N}>z_{1-\alpha /2}\right) & = & P\left({\left({\hat{\theta }}_{N}-\theta \right)}^{2}>\sigma_{N}^{2}c_{1-\alpha }\right)\;\\ & \approx & P\left({\left({\hat{\theta }}_{N}-\theta \right)}^{2}>\hat{q}\theta \left(1-\theta \right)c_{1-\alpha }\right)=\alpha ,\;\;\;\; \end{array}$$

where $c_{1-\alpha }=z_{1-\alpha /2}^{2}$ denotes the (1−α)‐quantile of the $\chi_{1}^{2}$‐distribution. The last expression in Equation (19) is used (1) to construct a test for the null hypothesis $H_{0}:\theta =1/2$ and (2) to derive a compatible confidence interval for the Mann–Whitney effect θ by using Wilson's idea (Wilson 1927).

An asymptotic test for $H_{0}:\theta =1/2$ is obtained by substituting θ=1/2 into Equation (19), yielding

(20) $$\begin{array}{c} C^{2}=\frac{4}{\hat{q}}\;\;{\left({\hat{\theta }}_{N}-1/2\right)}^{2}\;\;\overset{H_{0}}{∼}\;\;\chi_{1}^{2} \end{array}$$

and the hypothesis $H_{0}:\theta =1/2$ is rejected at the significance level α if $C^{2}>c_{1-\alpha }$. The corresponding p‐value is computed as p‐value $=1-F_{\chi_{1}^{2}}\left(C^{2}\right)$, where $F_{\chi_{1}^{2}}$ denotes the cumulative distribution function of the central $\chi_{1}^{2}$‐distribution. For the remainder of the paper, we will refer to this test as $C^{2}$‐test.

Remarks 4.2

 

1. As discussed by Brunner and Konietschke (2025) (Section 4.4), the DeLong estimator can be biased in either direction in general. For continuous distributions the bias is positive. Thus, the DeLong estimator overestimates the variance in case of no ties which counteracts the otherwise more liberal behavior of the Brunner–Munzel test. Consequently, simply replacing the biased DeLong estimator of the asymptotic variance with the unbiased estimator ${\hat{\sigma }}_{N}^{2}$ in Equation (6) would result in an even more liberal test in this case which can be demonstrated by simulations. We therefore decided not to pursue this approach further.
2. In Equation (17), an alternative approximation is given by ${\hat{r}}^{*}={\hat{\theta }}_{N}\left(1-{\hat{\theta }}_{N}\right)/\left[\left(m-1\right){\hat{\sigma }}_{N}^{2}\right]$ since Brunner and Konietschke (2025) showed that ${\hat{\sigma }}_{N}^{2}\leq {\hat{\theta }}_{N}\left(1-{\hat{\theta }}_{N}\right)/\left(m-1\right)$ which may be regarded as an “empirical Birnbaum–Klose” inequality. Then, letting $r\approx {\hat{r}}^{*}$ leads to the approximation $\sigma_{N}^{2}\;\approx \;\frac{m-1}{m}\;\hat{q}\;\theta \left(1-\theta \right)$, thereby inducing a slight dependency on the smaller sample size by the factor (m−1)/m where $m=\min\{n_{1},n_{2}\}$. For the sake of simplicity we will set this factor (which converges to 1 for m→∞) equal to 1 also for small‐sample sizes since simulations suggest that the resulting test does not demonstrate significant improvements.
3. The $C^{2}$‐statistic in Equation (20) can also be expressed as $C^{2}={\left({\hat{\theta }}_{N}-1/2\right)}^{2}/{\tilde{\sigma }}_{N}^{2}$, where
$$\begin{array}{ccc} {\tilde{\sigma }}_{N}^{2} & = & \frac{{\hat{\sigma }}_{N}^{2}}{4{\hat{\theta }}_{N}\left(1-{\hat{\theta }}_{N}\right)}\;. \end{array}$$
  This formulation shows that deriving a test based on the ratio of the true variance $\sigma_{N}^{2}$ to the maximum variance $\sigma_{N,\max}^{2}$ results in a testing procedure using the unbiased variance estimator ${\hat{\sigma }}_{N}^{2}$, scaled by the inflation factor $1/[4{\hat{\theta }}_{N}\left(1-{\hat{\theta }}_{N}\right)]\geq 1$. Hence, this approach yields a compromise between the true variance $\sigma_{N}^{2}$ and its theoretical upper bound 1/(4m) under the null hypothesis $H_{0}:\theta =1/2$, as $\sigma_{N}^{2}\;\leq \;\theta \left(1-\theta \right)/m\;\overset{H_{0}}{=}\;1/\left(4m\right)$.
4. While it is theoretically possible to replace the unbiased variance estimator in Equation (17) with the DeLong estimator ${\hat{v}}_{\text{DL}}^{2}/N$, thereby yielding a $C^{2}$‐test involving the DeLong variance estimator, we do not intend to pursue this option further. This decision is motivated by two considerations:
  a. The use of ${\hat{\sigma }}_{N}^{2}$ is naturally justified by the fact that this estimator has the sharp upper bound ${\hat{\theta }}_{N}\left(1-{\hat{\theta }}_{N}\right)/\left(m-1\right)\approx {\hat{\theta }}_{N}\left(1-{\hat{\theta }}_{N}\right)/m$ as noted in the previous item. Note also that the ratio ${\hat{r}}^{*}$ in Item 2 requires an upper bound of the variance estimator which is not known for the DeLong variance estimator.
  b. Simulation studies suggest that substituting the DeLong estimator yields no considerable improvement in performance.

Confidence intervals compatible with the $C^{2}$‐test can also be derived directly from Equation (19),

$$\begin{array}{c} P\left({\left({\hat{\theta }}_{N}-\theta \right)}^{2}\leq \hat{q}\;\theta \left(1-\theta \right)\;c_{1-\alpha }\right)\;\approx \;\;1-\alpha . \end{array}$$

Applying the ideas of Wilson (1927) for binomial proportions, an approximate 100(1−α)% confidence interval for θ is obtained by solving the quadratic inequality ${\left({\hat{\theta }}_{N}-\theta \right)}^{2}<\hat{q}\;c_{1-\alpha }\;\theta \left(1-\theta \right)$ for θ, leading to the confidence bounds

(21) $$\begin{array}{ccc} \theta_{L,U}^{r} & = & \frac{1}{2\left(1+\hat{q}c_{1-\alpha }\right)}\\ & & \times \left(2{\hat{\theta }}_{N}+\hat{q}\;c_{1-\alpha }\mp \sqrt{{\hat{q}}^{\;2}c_{1-\alpha }^{2}+4\;\hat{q}\;{\hat{\theta }}_{N}\left(1-{\hat{\theta }}_{N}\right)\;c_{1-\alpha }}\right). \end{array}$$

Here, the index r refers to the fact that we consider the ratio in Equation (16). By construction, the confidence interval $\theta_{L,U}^{r}$ is compatible with the decision derived from the $C^{2}$‐test since both procedures are derived from Equation (19) and it follows that $C^{2}>c_{1-\alpha }$ if $1/2\notin [\theta_{L}^{r},\theta_{U}^{r}]$ and vice versa.

Remarks 4.3

1. Note that, similar as the confidence intervals for binomial proportions by Wilson (1927), the confidence intervals in Equation (21) are asymmetric with respect to ${\hat{\theta }}_{N}$. This may be advantageous when the asymptotic normality of the estimator is questionable, e.g., in small samples and extreme effects. For details on the derivation, we refer to Appendix A.1.
2. The confidence interval $\theta_{L,U}^{r}$ in Equation (21) is range‐preserving by construction. The corresponding proof can be found in Appendix A.2. To illustrate this property, we revisit the data example in Table 2. Applying the $C^{2}$‐test to analyze the data, we obtain a p‐value of 0.0282 and the 95% confidence interval [0.53,0.93], which is both range‐preserving and compatible with the test decision.
3. The conclusion from the $C^{2}$‐test in Equation (20) is not compatible with the confidence interval derived from the Brunner–Munzel test or corresponding range‐preserving transformations, such as the logit‐ or probit‐transformation. Hence, the $C^{2}$‐test result should always be reported alongside the confidence interval in Equation (21).

In case of completely separated samples, we have ${\hat{\theta }}_{N}\in \left\{0,1\right\}$ and ${\hat{\sigma }}_{N}^{2}=0$, resulting in undefined values of the $C^{2}$‐test statistic. Although of limited practical relevance, this scenario may occasionally occur in simulations with small‐sample sizes, even for moderate deviations of θ from 1/2. Therefore, it is necessary to address this situation separately. To ensure that no distribution is excluded, we propose setting $\sigma_{N}^{2}=\sigma_{N,\max}^{2}=\theta \left(1-\theta \right)/m$, as the Birnbaum–Klose inequality is sharp. Consequently, there exist distributions for which the equality $\sigma_{N}^{2}=\theta \left(1-\theta \right)/m$ holds exactly. In this case, $\hat{q}$ in Equation (19) is replaced with the sharp upper bound of its theoretical counterpart $q=\sigma_{N}^{2}/\left[\theta \left(1-\theta \right)\right]\leq 1/m$. As a result, variance estimation is no longer required, leading to the simplified test statistic

(22) $$\begin{array}{ccc} C_{\sigma_{N,\max}}^{2} & = & 4\;m\;{\left({\hat{\theta }}_{N}-1/2\right)}^{2}\;. \end{array}$$

By plugging in ${\hat{\theta }}_{N}\in \left\{0,1\right\}$, this statistic becomes $C_{\sigma_{N,\max}}^{2}=m$ which only depends on the minimum sample size m. The p‐value of this test would then be given by $p\text{-value}=1-F_{\chi_{1}^{2}}\left(m\right)$. Hence for, a significance level of α=0.05 or α=0.01, this test will consistently reject the null hypothesis $H_{0}:\theta =1/2$ if m≥4 or m≥7, respectively. This result seems reasonable, as the criterion for rejecting the null hypothesis is expected to be comparatively liberal when observing effects as extreme as $\hat{\theta }\in \left\{0,1\right\}$.

To obtain compatible confidence intervals also in these extreme cases, we again approximate the true variance through the upper bound $\sigma_{N,\max}^{2}$ and solve the quadratic inequality ${\left({\hat{\theta }}_{N}-\theta \right)}^{2}\leq c_{1-\alpha }\theta \left(1-\theta \right)/m$ for θ, leading to the confidence interval

(23) $$\begin{array}{c} \theta_{L,U}^{\text{BK}}=\frac{1}{2(m+c_{1-\alpha })}\left(2m{\hat{\theta }}_{N}+c_{1-\alpha }\mp \sqrt{4m{\hat{\theta }}_{N}\left(1-{\hat{\theta }}_{N}\right)c_{1-\alpha }+c_{1-\alpha }^{2}}\right) \end{array}$$

analogously to the confidence interval in Equation (21). Here, the index BK refers to the fact that we use the bound of the Birnbaum–Klose inequality to replace the unknown variance (Birnbaum and Klose 1957). With ${\hat{\theta }}_{N}\in \left\{0,1\right\}$, those boundaries further simplify to

(24) $$\begin{array}{c} \theta_{L}^{\text{BK}}\left(1\right)=\frac{m}{m+c_{1-\alpha }}\;\text{and}\;\theta_{U}^{\text{BK}}\left(1\right)=1 \end{array}$$

and

(25) $$\begin{array}{c} \theta_{L}^{\text{BK}}\left(0\right)=0\;\text{and}\;\theta_{U}^{\text{BK}}\left(0\right)=\frac{c_{1-\alpha }}{m+c_{1-\alpha }}, \end{array}$$

hence turning into one‐sided confidence intervals for the extreme values of ${\hat{\theta }}_{N}$. Note that the confidence interval in Equation (23) does not require estimation of the variance $\sigma_{N}^{2}$. It remains conservative for all distributions  provided that the normal approximation of ${\hat{\theta }}_{N}$ can be considered as sufficiently accurate.

A more detailed investigation of the confidence interval in Equation (21) is beyond the scope of this paper and is considered as a separate research question requiring particular considerations for ‘larger’ values of θ or if the estimator ${\hat{\theta }}_{N}$ is ‘close’ or equal to the boundaries 0 or 1.

## 5 Simulation Study

Neubert and Brunner (2007) conducted extensive simulations demonstrating that the studentized permutation test effectively controls the preassigned type‐I error level α under the null hypothesis, even for very small‐sample sizes, outperforming the Brunner–Munzel test in this regard. Additionally, they provided some power simulation results. In this section, we compare both approaches to the $C^{2}$‐test by examining the type‐I error rate and assessing the power of all three methods. Furthermore, we evaluate the coverage of the corresponding confidence intervals.

### 5.1 Type‐I Error Rate Simulation

The distributions used for generating data considered in the type‐I error simulation study are summarized in Table 3. These settings encompass metric (discrete and continuous) and ordinal data. Additionally, equal and unequal variances, along with symmetric and skewed distributions, are considered. The Laplace distribution is included to also investigate heavy‐tailed distributions. The settings involving heteroscedastic distributions are depicted in the Supporting information, Section 1. For details on simulating ordered categorical data, we refer to Appendix A.3. We consider balanced as well as unbalanced designs involving the following combinations of sample sizes.

**TABLE 3:** Distributions considered in the type‐I error rate simulation study. All scenarios are chosen such that the null hypothesis that $H_{0}:\theta =1/2$ holds. This also includes the case of $F_{1}=F_{2}$. Note that for the Poisson and Exponential distributions, θ=1/2 can occur if and only if $F_{1}=F_{2}$. In the right column, $\kappa_{i}^{2}$ represents the variance of the distribution $F_{i}$.

Setting	Distribution	Parameters	$\kappa_{2}^{2}/\kappa_{1}^{2}$
1	Normal distribution^1^	$F_{1}=N\left(0,1\right)$ and $F_{2}=N\left(0,1\right)$	1
2	Normal distribution	$F_{1}=N\left(0,1\right)$ and $F_{2}=N\left(0,9\right)$	9
3	Beta distribution^1^	$F_{1}=B\left(1,1\right)$ and $F_{2}=B\left(1,1\right)$	1
4	Beta distribution^1^	$F_{1}=B\left(2,5\right)$ and $F_{2}=B\left(2,5\right)$	1
5	Beta distribution	$F_{1}=B\left(5,5\right)$ and $F_{2}=B\left(1,1\right)$	3.67
6	Beta distribution	$F_{1}=B\left(5,5\right)$ and $F_{2}=B\left(2,2\right)$	2.20
7	Ordered categorical data^1^	Based on $F_{1}=B\left(1,1\right)$ and $F_{2}=B\left(1,1\right)$	1
8	Ordered categorical data^1^	Based on $F_{1}=B\left(2,5\right)$ and $F_{2}=B\left(2,5\right)$	1
9	Ordered categorical data	Based on $F_{1}=B\left(5,5\right)$ and $F_{2}=B\left(1,1\right)$	3.67
10	Ordered categorical data	Based on $F_{1}=B\left(5,5\right)$ and $F_{2}=B\left(2,2\right)$	2.20
11	Poisson distribution^1^	$F_{1}=Pois\left(1\right)$ and $F_{2}=Pois\left(1\right)$	1
12	Exponential distribution^1^	$F_{1}=Exp\left(1\right)$ and $F_{2}=Exp\left(1\right)$	1
13	Laplace distribution^1^	$F_{1}=L\left(0,1\right)$ and $F_{2}=L\left(0,1\right)$	1
14	Laplace distribution	$F_{1}=L\left(0,1\right)$ and $F_{2}=L\left(0,3\right)$	9

1 $F_{1}=F_{2}$.

$$\begin{array}{cc} \left(n_{1},n_{2}\right) & \in \{(15,15),(30,30),(45,45),(60,60),(75,75)\}\\ \left(n_{1},n_{2}\right) & \in \{(15,30),(20,40),(30,60),(40,80),(50,100)\}\\ \left(n_{1},n_{2}\right) & \in \{(30,15),(40,20),(60,30),(80,40),(100,50)\}. \end{array}$$

That is, we consider the sample size ratios $n_{1}/n_{2}\in \left\{0.5,1,2\right\}$, covering both positive pairing (larger variance in the larger group) and negative pairing (larger variance in the smaller group) for normal, beta, and Laplace distributions in case of heteroscedastic distributions. Note that, to accommodate the asymptotic nature of the proposed method, we opted not to go below $n_{i}<15$ in our simulation study. The nominal α‐level is set to α∈{0.001,0.005,0.01,0.05}. This approach is motivated by recent recommendations advocating for the use of more stringent significance levels α<0.05 which are also used in α‐adjusting methods for multiple comparisons. We use $n_{\text{iter}}=100,000$ for all settings, giving rise to a Monte Carlo error of about 0.0001 to 0.0007 for α=0.001 to α=0.05. Finally, we estimate the type‐I error rate as $\frac{1}{n_{\text{iter}}}\sum_{i=1}^{n_{\text{iter}}}1_{\left\{\phi_{g}\left(X_{i}\right)=1\right\}}$ where 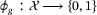 represents one of the statistical tests $g=\{C^{2},T_{N}^{\text{BM}},T_{\text{perm}}^{\text{BM}}\}$ considered throughout this manuscript. For the studentized permutation test, we set $n_{p}=10,000$ as the number of permutations.

For all analyses, we used our implementations in the software package R (Version 4.1.2) in conjunction with the RStudio IDE (Version 2022.07.1) (R Core Team 2021). In the case of separate samples, the variance estimator ${\hat{v}}_{\text{DL}}^{2}$ used for the Brunner–Munzel and the corresponding permutation test becomes zero such that corresponding tests and confidence intervals are undefined. Details on handling this singularity can be found in Appendix A.4. All code necessary to reproduce the presented results is included in the Supporting information and publicly available on GitHub at https://github.com/SteSchueuerhuis/C2‐Test‐Code.

**FIGURE 2:**
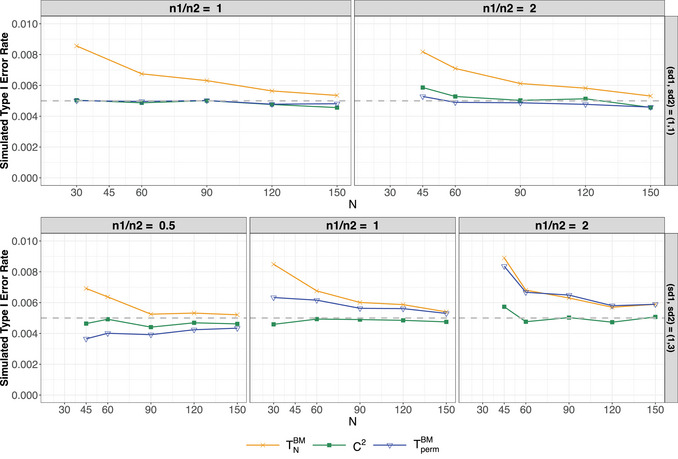
Simulated type‐I error rate for normal distributions $F_{1}=N\left(0,1\right)$ and $F_{2}=N\left(0,\sigma_{2}^{2}\right)$ based on 100,000 replications at a two‐sided nominal significance level of α=0.005 for all considered tests. In the upper row of the graphs, the variances are equal $\sigma_{1}^{2}=\sigma_{2}^{2}$ while in the lower row, $\sigma_{2}^{2}=9\sigma_{1}^{2}$. The results for the so‐called positive pairing are displayed in the left graph of the bottom row and the results for the negative paring in the right graph of the bottom row. The dashed gray line represents the nominal significance level.

**FIGURE 3:**
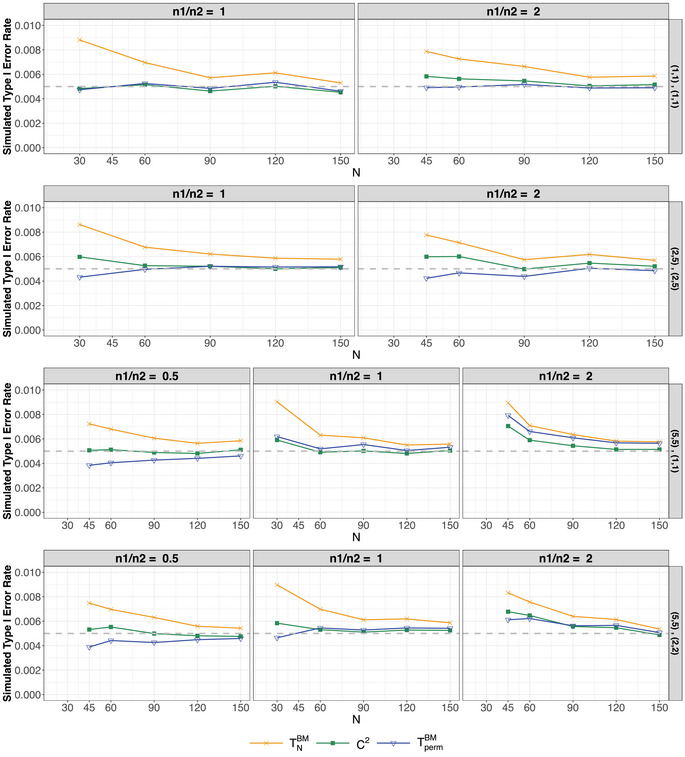
Simulated type‐I error rate for ordered categorical data settings 7–10 in Table 3 based on 100,000 replications at a two‐sided nominal significance level of α=0.005 for all considered tests. In the upper two rows of the graphs, the variances are equal while in the lower two rows the variances are unequal. In case of heteroscedastic samples, results for the positive pairing are displayed in the left graph and the results for the negative paring in the right graph. The dashed gray line represents the nominal significance level.

Exemplary results from the type‐I error rate simulations for settings 1, 2, and 7–10 in Table 3 are illustrated in Figures 2 and 3 for α=0.005. Additional simulation results are provided in the Supporting information. Here, we briefly summarize and discuss the findings for all tests included in the simulation study, beginning with a description of the plot setup. The columns of the plot represent different sample size settings. For homoscedastic settings, the balanced case is shown in the left column and the unbalanced case in the right column. For heteroscedastic samples, the left column corresponds to $n_{2}=2n_{1}$, the center column to $n_{1}=n_{2}$, and the right column to $n_{1}=2n_{2}$. The rows correspond to different parameter settings for the respective distributions. For example, in the lower row of Figure 2, $F_{1}=N\left(0,1\right)$ and $F_{2}=N\left(0,9\right)$. Here, the lower right plot illustrates the case of negatively paired variances, while the lower left plot represents positive pairing. In all figures, the x‐axis shows the total sample size $N=n_{1}+n_{2}$, and the y‐axis displays the simulated type‐I error rate. The gray dashed line indicates the target nominal α‐level. Based on the presented figures and the comprehensive simulation study detailed in the Supporting information, the simulation results can be summarized as follows:

- The **Brunner–Munzel test** ($T_{N}^{\text{BM}}$), represented by the orange lines with crosses, demonstrates liberal behavior for small‐sample sizes across most examined scenarios, regardless of the underlying distribution. This tendency becomes more pronounced as the significance level decreases. However, as the sample size increases, the type‐I error rate converges to the nominal α‐level. These findings align with previous research, such as Noguchi et al. (2021) and Pauly et al. (2016), which similarly observed that the test exhibits a liberal tendency, particularly at smaller significance levels.
- The **studentized permutation test** ($T_{\text{perm}}^{\text{BM}}$), represented by the blue line with triangles, exhibits improved type‐I error rate control across nearly all underlying distributions, particularly for small‐sample sizes. This suggests that the studentized permutation distribution effectively approximates the null distribution of the Brunner–Munzel test statistic, aligning with the theoretical foundation established by Pauly et al. (2016), who also demonstrated that the permutation test can robustly control the type‐I error rate for even smaller sample sizes. However, it is important to note that when variances are either negatively (larger variance in smaller group) or positively paired (larger variance in larger group), the studentized permutation test appears to inherit the behavior of the original Brunner–Munzel test, exhibiting liberal or conservative tendencies, respectively. This pattern is evident, for instance, in the lower row of Figure 2, as well as in cases involving heteroscedastic Beta distributions and corresponding 5‐point Likert scales.
- The $C^{2}$ **‐test** (green line with squares) demonstrates improved type‐I error rate control compared to the Brunner–Munzel test. This improvement generally holds across different underlying distributions and significance levels α, suggesting that the $\chi_{1}^{2}$‐approximation used in Equation (20) is valid. The only exception arises in small‐sample scenarios involving highly skewed 5‐point Likert‐scale data, as illustrated in Figure 3, where the underlying distribution is $F_{i}=B\left(2,5\right),i=1,2$. Here, the test slightly exceeds the target α‐level. This behavior may be attributed to the extreme skewness of the simulated data, where the discretization causes most values to fall into a low number of categories. As a result, the data may be close to an empirical one‐point distribution, causing variance estimation to degenerate and leading to liberal type‐I error rates. Note also that for α=0.05, the $C^{2}$‐test may tend to be slightly conservative compared to the Brunner–Munzel test, in particular in setting 2 and setting 5 of Table 3 (see Supporting information).

### 5.2 Power Simulation

In the previous section, we examined the type‐I error rate, illustrating that the proposed $C^{2}$‐test has decent small‐sample size properties. In practice, we are additionally concerned with the statistical power of the tests. Table 4 summarizes the settings we consider for power simulation. The generate alternatives, we determine the distributional parameters μ,α, and λ such that they correspond to some target Mann–Whitney effect $\tilde{\theta }>0.5$. Exemplary distributions are depicted in the Supporting information, Section 1. Regarding sample sizes, we consider $\left(n_{1},n_{2}\right)\in \left\{\left(15,15\right),\left(15,30\right),\left(30,15\right)\right\}$ to encompass both balanced and unbalanced sample sizes. We again used 100,000 iterations in the simulation study.

**TABLE 4:** Distributions considered in the power simulation study. All scenarios are chosen such that the alternative hypothesis $H_{1}:\theta =\tilde{\theta }$ for some $\tilde{\theta }>0.5$ holds.

Setting	Distribution	Control	Treatment
1	Normal distribution	$F_{1}=N\left(0,1\right)$	$F_{2}=N\left(\mu ,1\right)$
2	Normal distribution	$F_{1}=N\left(0,1\right)$	$F_{2}=N\left(\mu ,9\right)$
3	Ordered categorical data	Based on $F_{1}=B\left(1,1\right)$	Based on $F_{2}=B\left(\alpha ,1\right)$
4	Exponential distribution	$F_{1}=Exp\left(\lambda \right)$	$F_{2}=Exp\left(1\right)$

Since the results for the other distributions are virtually identical, we present only the power for settings 1 and 2 in the normal distribution case (see Table 4) in the main manuscript. The results for the other settings are provided in the Supporting information. The x‐axes represent the true Mann–Whitney effect θ, while the y‐axes display the simulated power. The gray dashed line indicates the conventionally chosen target power level of 80%. Note that power curves appeared to be largely overlapping.

**FIGURE 4:**
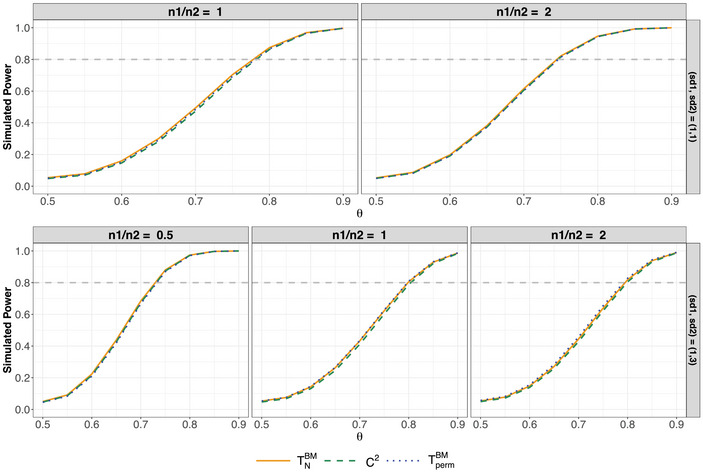
Power for normal distributions $F_{1}=N\left(0,1\right)$ and $F_{2}=N\left(\mu ,\sigma_{2}^{2}\right)$ based on 100,000 replications at a two‐sided nominal significance level of α=0.05. In the upper row, $\sigma_{2}^{2}=1$ while $\sigma_{2}^{2}=9$ in the lower row. $F_{1}$ and $F_{2}$ are chosen such that θ∈[0.5,0.9]. The dashed gray lines indicate the target power of 80%.

Figure 4 illustrates that differences in power among the tests are minimal, regardless of the variances or sample size configurations. Overall, the Brunner–Munzel and the permutation tests exhibit slightly higher power than the $C^{2}$‐test, although these differences are negligible. Notable discrepancies occur only when $\sigma_{1}^{2}<\sigma_{2}^{2}$ while $n_{1}>n_{2}$, as highlighted in the bottom‐right panel of Figure 4. However, it should be noted that the slightly higher power of the Brunner–Munzel and permutation test likely stems from their slight liberality in scenarios involving negatively paired variances and small‐sample sizes, as illustrated in the bottom‐right plots of Figure 2. These findings conclude that the $C^{2}$‐test achieves satisfactory power while providing improved control of the type‐I error rate across the scenarios considered compared to the Brunner–Munzel test.

### 5.3 Coverage Simulation

**FIGURE 5:**
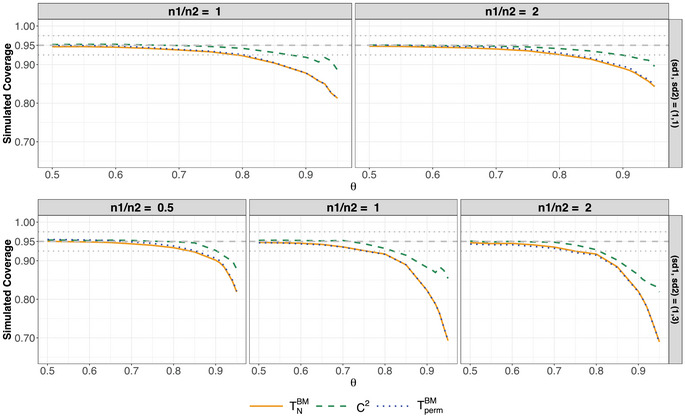
Simulated coverage probability for normal distributions $F_{1}=N\left(0,1\right)$ and $F_{2}=N\left(0,\sigma_{2}^{2}\right)$ based on 100,000 replications at a two‐sided nominal significance level of α=0.05 for all considered confidence intervals. In the upper row, $\sigma_{2}^{2}=1$ while $\sigma_{2}^{2}=9$ in the lower row. Gray dotted lines represent the robust Bradley (1978) limits [0.925,0.975].

Lastly, we briefly address the coverage probabilities of the confidence intervals in Equations (12), (14), and (21), derived from the Brunner–Munzel test, the studentized permutation test, and the $C^{2}$‐test. To this end, we simulated normally distributed data as outlined in Table 4, considering Mann–Whitney effects ranging from 0.5 to 0.95, ensuring consideration of rather extreme effects. Figure 5 presents the results for homoscedastic samples in the top row (left: $n_{1}=n_{2}=15$; right: $\left(n_{1},n_{2}\right)=\left(30,15\right)$) and for heteroscedastic samples in the bottom row (left: $\left(n_{1},n_{2}\right)=\left(15,30\right)$; center: $n_{1}=n_{2}=15$; right: $\left(n_{1},n_{2}\right)=\left(30,15\right)$).

In Figure 5, all methods show a reasonable coverage at small values of θ. However, as θ increases, all confidence intervals tend to become liberal at some point. Specifically, the confidence intervals derived from inverting the Brunner–Munzel test and the studentized permutation test show a decline in coverage, falling below 0.925 for approximately θ>0.8 in the homoscedastic case (upper row). In contrast, the confidence interval associated with the $C^{2}$‐test maintains higher coverage over a broader range of θ. Notably, its coverage drops below 0.925 only at θ>0.9 in the unbalanced case and θ>0.85 in the balanced case. A similar trend is observed in the heteroscedastic scenario (lower row), where the $C^{2}$‐test also extends the range of θ for which coverage remains adequate, outperforming the alternative methods. In conclusion, the $C^{2}$‐test provides a compatible confidence interval with improved coverage across a wider range of θ while additionally being range‐preserving. Methods aimed at improving coverage specifically for extreme values of θ deserve particular attention, as emphasized by Perme and Manevski (2019). While the confidence interval based on the $C^{2}$‐test already demonstrates an improvement over competing methods, addressing its behavior at the boundaries remains an open question for future research.

The results of the coverage probability simulations can also be interpreted in the context of diagnostic testing. To this end, note that the Mann–Whitney effect θ is known to be equivalent to the area under the receiver operating characteristic (ROC) curve (AUC), a common measure of discriminatory accuracy in diagnostic testing (see, e.g., Bamber, 1975; Pauly et al., 2016). Although testing the null hypothesis of no discriminatory power, i.e., $H_{0}:\theta =1/2$, is primarily of interest, Pauly et al. (2016) emphasized that it may also be relevant to test hypotheses for arbitrary AUC values. This is particularly important when evaluating whether a new diagnostic test or biomarker shows improved accuracy over an existing one with an assumed AUC of $\theta_{0}>1/2$. To test the hypothesis $H_{0}:\theta =\theta_{0}$, a test statistic $C_{\theta_{0}}^{2}$ can be derived from Equation (19) by substituting θ with $\theta_{0}$ and the null hypothesis is rejected if $C_{\theta_{0}}^{2}>c_{1-\alpha }$. Since rejecting $H_{0}:\theta =\theta_{0}$ is equivalent to observing that $\theta_{0}\notin \left[\theta_{L}^{r},\theta_{U}^{r}\right]$, the simulation results on coverage suggest that the test maintains the nominal α‐level for moderately large values of $\theta_{0}$. However, because the AUC is a composite measure that summarizes both sensitivity and specificity, evaluating diagnostic accuracy may require a more detailed analysis of these two components separately. Since the focus of this paper is on deriving a test for the nonparametric Behrens–Fisher problem, a detailed investigation of the confidence interval's coverage probability and the test for $H_{0}:\theta =\theta_{0}\neq 1/2$, respectively, is beyond the scope of this work.

## 6 Analysis of the Example

In this section, we revisit the data from the shoulder tip pain study in Section 2, which aimed to determine whether a specific suction procedure reduces pain in patients after laparoscopic surgery more effectively than the standard of care. In this context, values of θ>0.5 indicate a tendency toward lower pain scores in the treatment group, suggesting the procedure's effectiveness, while values of θ<0.5 favor the control group. The results are summarized in Table 5.

**TABLE 5:** Results of the shoulder tip pain trial in Section 2.

Descriptive results		
Sample sizes	$n_{1}=19$ (Control)	$n_{2}=22$ (Treatment)
Mann–Whitney effect	${\hat{\theta }}_{N}=0.837$	
Variance estimates	${\hat{v}}_{\text{DL}}^{2}=0.172$	(Brunner–Munzel test)
	$N{\hat{\sigma }}_{N}^{2}=0.171$	($C^{2}$‐test)

Tests and confidence intervals			
	Brunner–Munzel test	Stud. perm. test	$C^{2}$‐test
Test statistic	5.20	5.20	14.9
p‐Value	0.0000187	0.0001 *	0.000116
Decision (α=5%)	Reject $H_{0}$	Reject $H_{0}$	Reject $H_{0}$
95% confidence intervals	[0.704,0.970]	[0.708,0.970]	[0.677,0.927]

* None of the permuted test statistics was greater than or equal to the observed test statistic. Following Section 4.2, we therefore report $1/\left(n_{p}+1\right)=1/\left(10,000+1\right)\approx 0.0001$ as the minimal p‐value.

We observe that the DeLong variance estimate (used in the Brunner–Munzel test) and the unbiased variance estimate (used in the $C^{2}$‐test) are quite close. Regarding the confidence intervals, we observe a slightly larger upper bound for the interval corresponding to the Brunner–Munzel test compared to a slightly smaller lower bound for the interval corresponding to the $C^{2}$‐test. Despite these minor differences, all three tests consistently lead to the conclusion that the null hypothesis $H_{0}:\theta =1/2$ can be rejected at a nominal significance level of α=0.05 in favor of the treatment group, indicating that the specific suction procedure significantly reduced the pain scores in comparison to the control treatment.

## 7 Discussion and Outlook

The nonparametric Behrens–Fisher problem of testing $H_{0}:\theta =1/2$ addresses the comparison of two independent groups without relying on the assumption of equal variances or normality, where θ denotes the well‐known Mann–Whitney effect. Since θ is well‐defined even for discrete data, this hypothesis is especially useful when parametric assumptions are doubtful or when it is inappropriate to impose distributional assumptions. Consequently, the nonparametric Behrens–Fisher problem offers a flexible and broadly applicable framework. To date, the Brunner–Munzel test (Brunner and Munzel 2000) is the primarily recommended method for testing $H_{0}:\theta =1/2$. However, it faces important limitations: first, it tends to exceed the nominal type‐I error rate at small‐sample sizes and low‐significance levels, which limits its validity, particularly when adjustments for multiple testing are necessary. Second, compatible confidence intervals may become liberal for large effect sizes and are not range‐preserving by construction. On the other hand, range‐preserving confidence intervals obtained by the logit‐ or probit‐transformation are not compatible with the decisions from the Brunner–Munzel test. These limitations served as motivation to revisit the nonparametric Behrens–Fisher problem.

To address these drawbacks, we proposed a new closed‐form test based on considering the ratio of the true variance $\sigma_{N}^{2}$ of ${\hat{\theta }}_{N}$ to its theoretical upper bound $\sigma_{N,\max}^{2}$, as derived from the Birnbaum–Klose inequality (Birnbaum and Klose 1957). Additionally, we developed compatible, range‐preserving confidence intervals for this procedure. The performance of the proposed method was extensively evaluated through simulations by comparing it to the Brunner–Munzel test and the corresponding studentized permutation test (Neubert and Brunner, 2007; Pauly et al., 2016) across a wide range of scenarios, focusing on type‐I error rate control, statistical power, and the coverage probability of the corresponding confidence intervals.

In the simulation study, we observed that the $C^{2}$‐test demonstrates improved type‐I error rate control in small‐sample sizes compared to the Brunner–Munzel test even at significance levels as small as α=0.005. Hence, the presented methods align with recent recommendations questioning the use of α=0.05 for hypothesis testing. Furthermore, the proposed test is comparable to the studentized permutation test by Neubert and Brunner (2007) regarding type‐I error control. While the studentized permutation test slightly exceeded the nominal α‐level in cases of negatively paired variances, the proposed test is slightly liberal under highly skewed ordered categorical data conditions. In terms of power, the observed differences were mainly negligible. Regarding coverage of the confidence intervals, we observed that the proposed confidence interval maintains adequate coverage over a broader range of θ compared to the competing methods. Finally, the proposed method has a closed‐form expression rather than relying on resampling. This improves runtime efficiency, which can be particularly beneficial for simulation‐based study planning. Based on the simulation results, we believe that the proposed method could serve as a useful alternative to both the Brunner–Munzel test and the studentized permutation test in practical applications.

Despite its advantages, the proposed method also has some limitations that warrant consideration. First, it relies on the asymptotic normality of ${\hat{\theta }}_{N}$, which may be questionable for smaller sample sizes ($n_{i}<15$) per group. In those cases, we recommend computing p‐values and confidence intervals using studentized permutations, which are also appropriate for small‐sample sizes as small as $n_{i}=10$ (Pauly et al. 2016). Second, similar to the competitor methods, the proposed confidence interval tends to exhibit liberal coverage when effect sizes approach the boundaries. Hence, confidence intervals under small‐sample sizes and large effects should be interpreted cautiously. Addressing this issue in the presence of extreme effects remains an open question for future research.

Alongside investigating properties under extreme effects, the new method provides a basis for numerous further research directions. First, it distinguishes itself from established approaches by incorporating the unbiased variance estimator used by Brunner and Konietschke (2025). When combined with range‐preserving transformations, such as logit‐ or probit‐transformations, this estimator could be integrated into modified studentized permutation procedures to potentially enhance the performance of the tests and confidence intervals proposed in Pauly et al. (2016), particularly for very small‐sample sizes. However, this paper intends to focus on tests that can be expressed through explicit formulas. Additionally, incorporating the Birnbaum–Klose inequality to improve adherence to type‐I error rate control could be leveraged to refine methods in other fields of application. For example, in diagnostic testing, a common research question is to compare biomarkers with respect to their ability to distinguish between diseased and non‐diseased individuals, i.e., to test $H_{0}:\theta_{1}=\theta_{2}$, where $\theta_{1}$ and $\theta_{2}$ represent Mann–Whitney effects referring to the two biomarkers. A straightforward adaptation could replace the variance estimator in the commonly used DeLong test with that of Brunner and Konietschke (2025). In contrast, techniques similar to those used for the $C^{2}$‐test could be developed to investigate alternative methods, particularly for small‐sample sizes. Another promising research area involves generalized pairwise comparisons (GPC), as introduced by Buyse (2010), which extend standard nonparametric methods to multivariate prioritized outcomes using U‐statistics. Adapting the proposals to the GPC framework might be possible as estimators such as ${\hat{\theta }}_{N}$ and ${\hat{\sigma }}_{N}^{2}$ should remain valid when derived from U‐statistics with count functions rather than empirical distribution functions and ranks. However, establishing the theoretical underpinnings of this approach remains an open challenge. Finally, practical considerations such as sample size planning for the $C^{2}$‐test or the extension to several samples and paired data represent important areas of research.

## Author Contributions

St.S. contributed to developing the methods, wrote the manuscript, coded, and created the tables and figures. F.K. provided critical review of the manuscript. E.B. proposed the paper project and suggested to evaluate the ratio method, contributed to the development of the methods and provided supervision of the project. All authors read and approved the final manuscript.

## Funding

This work was supported by the German Research Foundation (Grants KO 4680/4‐2 and 4680/6‐1).

## Conflicts of Interest

The authors declare no conflicts of interest.

## Open Research Badges

This article has earned an Open Data badge for making publicly available the digitally‐shareable data necessary to reproduce the reported results. The data is available in the Supporting Information section.

This article has earned an open data badge “**Reproducible Research**” for making publicly available the code necessary to reproduce the reported results. The results reported in this article could fully be reproduced.

## Supporting information

Supporting information

Supporting Information

## Data Availability

Original data presented in Section 2 were analyzed. The data were taken from Lumley (1996) and also analyzed in the papers of Brunner and Munzel (2000) and Neubert and Brunner (2007). All code used to reproduce the results in this paper is publicly available on GitHub at https://github.com/SteSchueuerhuis/C2‐Test‐Code. We intend to integrate the methods into the R package rankFD upon publication.

## Appendix A

### A.1 Derivation of the Ratio Method Confidence Interval

We derive the ratio method confidence intervals $\theta_{L,U}^{r}$ in Equation (21) by solving the quadratic inequality

$$\begin{array}{c} {\left({\hat{\theta }}_{N}-\theta \right)}^{2}\geq \hat{q}c_{1-\alpha }\theta \left(1-\theta \right), \end{array}$$

where $\hat{q}=\frac{{\hat{\sigma }}_{N}^{2}}{{\hat{\theta }}_{N}\left(1-{\hat{\theta }}_{N}\right)}$, for θ:

$$\begin{array}{cc} & \;{\left({\hat{\theta }}_{N}-\theta \right)}^{2}\geq \hat{q}c_{1-\alpha }\theta \left(1-\theta \right)\\ & ⟺\;\underset{=a}{\underset{︸}{\left(1+\hat{q}c_{1-\alpha }\right)}}\theta^{2}\underset{=b}{\underset{︸}{-(2{\hat{\theta }}_{N}+\hat{q}c_{1-\alpha })}}\theta +\underset{=c}{\underset{︸}{{\hat{\theta }}_{N}^{2}}}\geq 0\\ & ⟺\;a\theta^{2}+b\theta +c\geq 0. \end{array}$$

Solving this quadratic inequality results in the solutions

$$\begin{array}{ccc} \theta_{L,U}^{r} & = & \frac{\left(2{\hat{\theta }}_{N}+\hat{q}c_{1-\alpha }\right)\mp \sqrt{{\left(2{\hat{\theta }}_{N}+\hat{q}c_{1-\alpha }\right)}^{2}-4\left(1+\hat{q}c_{1-\alpha }\right){\hat{\theta }}_{N}^{2}}}{2\left(1+\hat{q}c_{1-\alpha }\right)} \end{array}$$

By further simplification, we obtain that

$$\begin{array}{ccc} \theta_{L,U}^{r} & = & \frac{1}{2\left(1+\hat{q}c_{1-\alpha }\right)}\left(2{\hat{\theta }}_{N}+\hat{q}c_{1-\alpha }\mp \sqrt{{\hat{q}}^{2}c_{1-\alpha }^{2}+4\;\hat{q}\;{\hat{\theta }}_{N}\left(1-{\hat{\theta }}_{N}\right)c_{1-\alpha }}\right) \end{array}$$

which is the confidence interval given in Equation (21).

### A.2 Proof That the Confidence Interval $\theta_{L,U}^{r}$ in Equation (21) is Range‐Preserving

In Remark 4.2, we stated that the confidence interval $\theta_{L,U}^{r}$ is range‐preserving. We will prove this property by contradiction. Recall that the confidence interval is given by

$$\begin{array}{ccc} \theta_{L,U}^{r} & = & \frac{1}{2\left(1+\hat{q}c_{1-\alpha }\right)}\left(2{\hat{\theta }}_{N}+\hat{q}c_{1-\alpha }\mp \sqrt{{\hat{q}}^{2}c_{1-\alpha }^{2}+4\;\hat{q}\;{\hat{\theta }}_{N}\left(1-{\hat{\theta }}_{N}\right)c_{1-\alpha }}\right) \end{array}$$

  where $\hat{q}=\frac{{\hat{\sigma }}_{N}^{2}}{{\hat{\theta }}_{N}\left(1-{\hat{\theta }}_{N}\right)}$. If the confidence interval was not range preserving, it must be possible that $\theta_{U}^{r}>1$, that is

$$\begin{array}{ccc} 2{\hat{\theta }}_{N}+\sqrt{{\hat{q}}^{2}c_{1-\alpha }^{2}+4\;\hat{q}\;{\hat{\theta }}_{N}\left(1-{\hat{\theta }}_{N}\right)c_{1-\alpha }} & > & 2+\hat{q}\;c_{1-\alpha }\;, \end{array}$$

which can be rewritten to $0>{\left(1-{\hat{\theta }}_{N}\right)}^{2}+\hat{q}\;c_{1-\alpha }\;{\left(1-{\hat{\theta }}_{N}\right)}^{2}$ by straightforward computations. This, however, is a contradiction since all quantities on the right‐hand side are non‐negative. By similar arguments, it follows that $\theta_{L}^{r}\geq 0$.

### A.3 Simulation of Ordered Categorical Data

To simulate ordinal data, we partition the data into J categories $C_{1}<\cdots \;<C_{J}$, where a higher index signifies a more favorable outcome. Following the approach outlined by Brunner et al. (2021) and Nowak et al. (2022), we initiate the simulation by generating data from a Beta‐distribution. Let $Y_{ik}∼B\left(\alpha_{i},\beta_{i}\right)$, where i∈{1,2} and $k=1,\cdots ,n_{i}$, denotes a random variable following a Beta‐distribution with parameters $\alpha_{i}>0$ and $\beta_{i}>0$. These parameters are chosen such that:

$$\begin{array}{cc} E[Y_{ik}] & =\frac{\alpha_{i}}{\alpha_{i}+\beta_{i}}\\ V[Y_{ik}] & =\frac{\alpha_{i}\beta_{i}}{{\left(\alpha_{i}+\beta_{i}\right)}^{2}\left(\alpha_{i}+\beta_{i}+1\right)}. \end{array}$$

Using this, we create ordered categorical data $X_{ik},i\in \left\{1,2\right\},i=1,\cdots ,n_{i}$, according to the intervals:

$$\begin{array}{c} X_{ik}=C_{j}\;\text{if}\;Y_{ik}\in \left[\frac{\left(j-1\right)}{J},\frac{j}{J}\right) \end{array}$$

for j=1,⋯,J. This results in the probability mass function:

$$\begin{array}{c} P\left(X_{ik}=C_{j}\right)=P\left(Y_{ik}\in \left[\frac{\left(j-1\right)}{J},\frac{j}{J}\right)\right). \end{array}$$

To mimic the commonly employed 5‐point Likert scale data, we set J=5 such that

$$\begin{array}{ccc} X_{ik} & = & \left\{\begin{array}{cc} C_{1} & \;\text{if}\;Y_{ik}\in \left[0,0.2\right)\\ C_{2} & \;\text{if}\;Y_{ik}\in \left[0.2,0.4\right)\\ C_{3} & \;\text{if}\;Y_{ik}\in \left[0.4,0.6\right)\\ C_{4} & \;\text{if}\;Y_{ik}\in \left[0.6,0.8\right)\\ C_{5} & \;\text{if}\;Y_{ik}\in \left[0.8,1\right]. \end{array}\right. \end{array}$$

By adjusting $\alpha_{i}$ and $\beta_{i}$, it is possible to construct both homoscedastic and heteroscedastic data distribution settings. Furthermore, we can select $\alpha_{i}$ and $\beta_{i}$ to match a target Mann–Whitney effect $\tilde{\theta }$, i.e., to fulfill $\int F_{1}dF_{2}=\tilde{\theta }$. In our power simulations, we achieve this by fixing three of the four parameters and solving for the remaining parameter to attain the target effect. The following relation facilitates this. With J denoting the number of categories and $0=c_{0}<c_{1}<\cdots <c_{J-1}<c_{J}=1$ the cutoff‐values, the Mann–Whitney effect is given through

$$\begin{array}{cc} \theta & =P\left(X_{2i}>X_{1j}\right)+1/2P\left(X_{2i}=X_{1j}\right)\\ & =\sum_{i=2}^{J}\left\{\left(F_{\alpha_{2},\beta_{2}}\left(c_{i}\right)-F_{\alpha_{2},\beta_{2}}\left(c_{i-1}\right)\right)\left[\sum_{l=1}^{i-1}\left(F_{\alpha_{1},\beta_{1}}\left(c_{l}\right)-F_{\alpha_{1},\beta_{1}}\left(c_{l-1}\right)\right)\right]\right\}\\ & +1/2\sum_{i=1}^{J}\left[F_{\alpha_{1},\beta_{1}}\left(c_{i}\right)-F_{\alpha_{1},\beta_{1}}\left(c_{i-1}\right)\right]\left[F_{\alpha_{2},\beta_{2}}\left(c_{i}\right)-F_{\alpha_{2},\beta_{2}}\left(c_{i-1}\right)\right] \end{array}$$

### A.4 Separate Sample Handling

In case of completely separated samples $X_{1}=\left(X_{11},\cdots ,X_{1n_{1}}\right)$ and $X_{2}=\left(X_{21},\cdots ,X_{2n_{2}}\right)$ (e.g., if $\min\left\{X_{2}\right\}>\max\left\{X_{1}\right\}$ or vice versa), we have that ${\hat{\theta }}_{N}\in \left\{0,1\right\}$ and ${\hat{\sigma }}_{N}^{2}=0$ or ${\hat{v}}_{\text{DL}}^{2}=0$, leading to undefined values of the test statistics under consideration. We must treat this case separately to address this singularity in our simulation study. Here, we briefly introduce our exception‐handling approach for all methods.

#### A.4.1 $C^{2}$‐Test

For the $C^{2}$‐test, the case of separate samples has already been addressed in the main manuscript, where we proposed replacing the ratio approximation with the maximum variance estimate, $\sigma_{N,\max}^{2}=\theta \left(1-\theta \right)/m$, thereby eliminating the need for variance estimation.

#### A.4.2 Brunner–Munzel Test and Studentized Permutation Test

In the case of separate samples, the variance estimate ${\hat{v}}_{\text{DL}}^{2}$ becomes zero, leading to an undefined value of $T_{N}^{\text{BM}}$ as well as undefined confidence bounds for both the Brunner–Munzel test and the studentized permutation test. Here, we employed the “one‐step‐back” method as described in Brunner et al. (2019) (Section 3.5.3). This method adjusts ${\hat{\theta }}_{N}$ for extreme cases (${\hat{\theta }}_{N}=0$ or ${\hat{\theta }}_{N}=1$) by selecting the closest possible values of ${\hat{\theta }}_{N}$ to 0 or 1 and the smallest non‐zero variance estimator, based on minimal overlap (nearly separated samples). More precisely,

- When no ties occur, ${\hat{\theta }}_{N}=1-1/\left(n_{1}n_{2}\right)$ is the closest value to 1, and ${\hat{\theta }}_{N}=1/\left(n_{1}n_{2}\right)$ is the closest value to 0 and the smallest non‐zero variance estimator is given by ${\hat{v}}_{\text{DL}}^{2}=2N/\left(n_{1}^{2}n_{2}^{2}\right)$.
- In the presence of ties, the smallest overlap corresponds to a single tied value. In this case, ${\hat{\theta }}_{N}=1-1/\left(2n_{1}n_{2}\right)$ is closest to 1, and ${\hat{\theta }}_{N}=1/\left(2n_{1}n_{2}\right)$ is closest to 0 and the smallest non‐zero variance estimator becomes ${\hat{v}}_{\text{DL}}^{2}=N/\left(2n_{1}^{2}n_{2}^{2}\right)$.
