# Supplementary material for: A New Approach to the Nonparametric Behrens–Fisher Problem With Compatible Confidence Intervals
Source: Biom J. 2025 Nov 9;67(6):e70096. doi: 10.1002/bimj.70096 (PMC12598137; doi:10.1002/bimj.70096)
Supplement: Supplementary file 1 — Supporting information [file BIMJ-67-e70096-s002.zip › Schüürhuis_et_al_code_R2/R Code Submission/plots/Supplement/section1.1_table3-het.pdf]

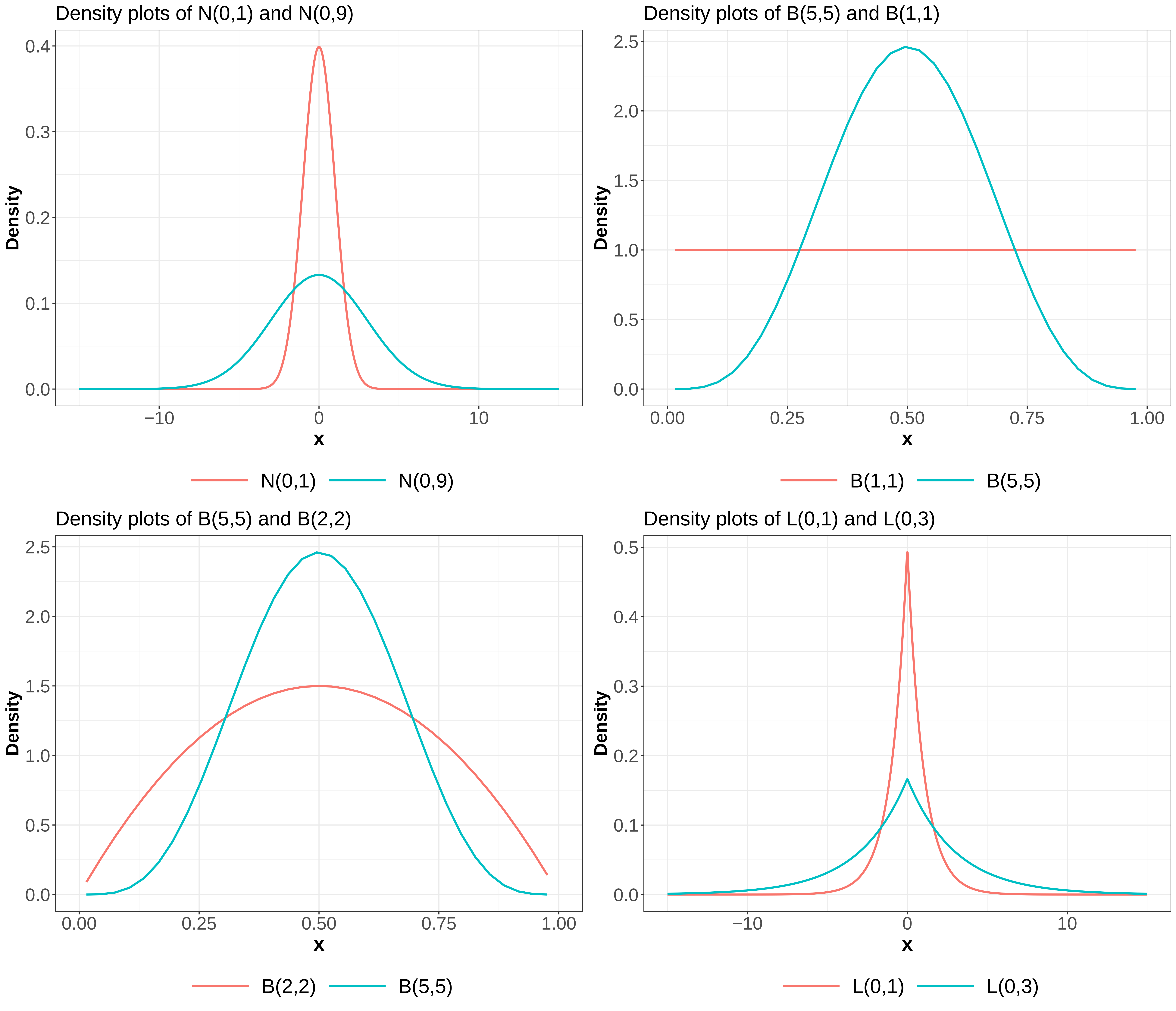

Density plots of N(0,1) and N(0,9)
Density
x
N(0,1)
N(0,9)
Density plots of B(5,5) and B(1,1)
Density
x
B(1,1)
B(5,5)
Density plots of B(5,5) and B(2,2)
Density
x
B(2,2)
B(5,5)
Density plots of L(0,1) and L(0,3)
Density
x
L(0,1)
L(0,3)
