# Supplementary material for: A New Approach to the Nonparametric Behrens–Fisher Problem With Compatible Confidence Intervals
Source: Biom J. 2025 Nov 9;67(6):e70096. doi: 10.1002/bimj.70096 (PMC12598137; doi:10.1002/bimj.70096)
Supplement: Supplementary file 1 — Supporting information [file BIMJ-67-e70096-s002.zip › Schüürhuis_et_al_code_R2/R Code Submission/plots/Supplement/section1.2_table4.pdf]

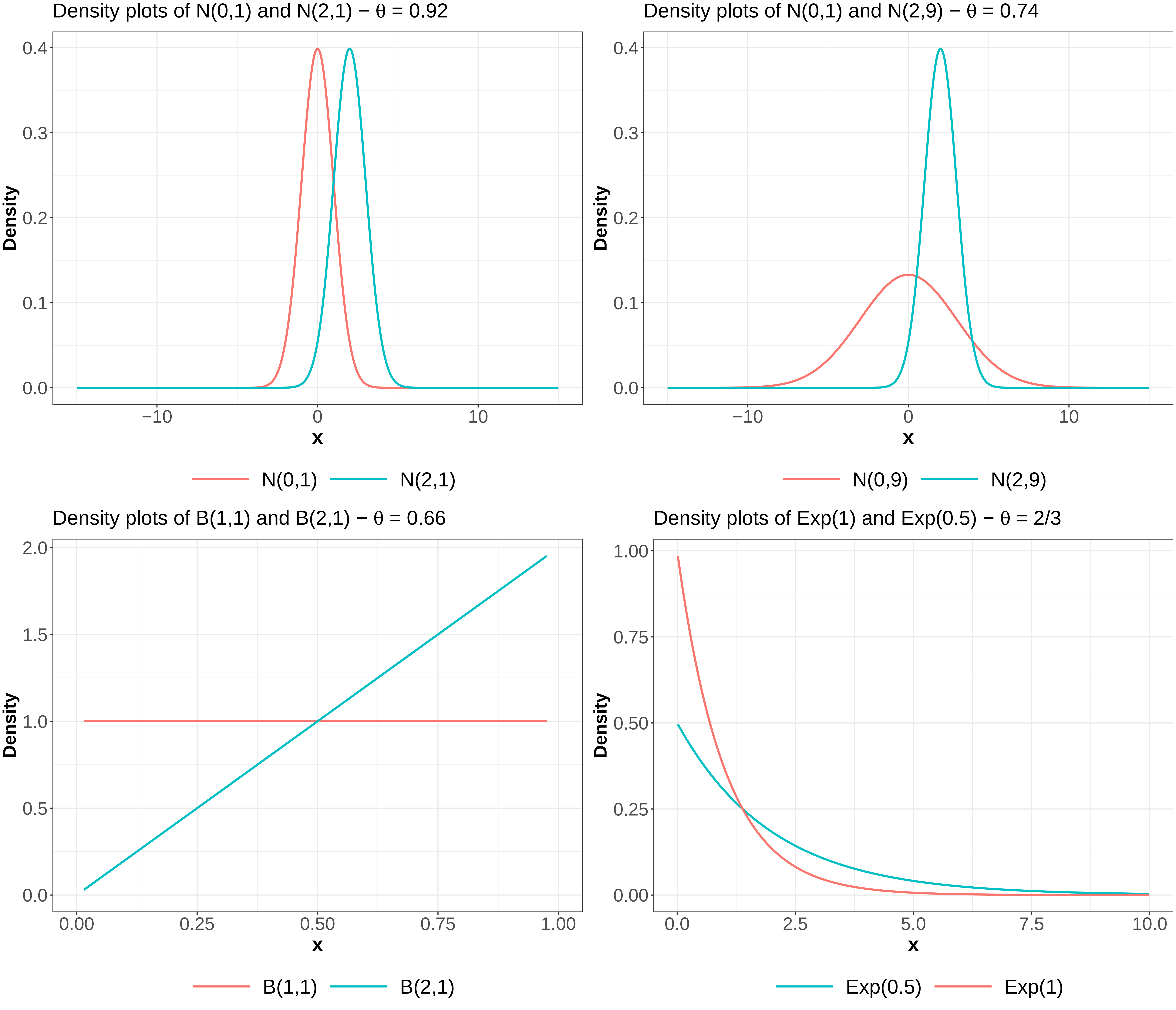
Density plots of N(0,1) and N(2,1) − θ = 0.92
Density
x
N(0,1)
N(2,1)
Density plots of N(0,1) and N(2,9) − θ = 0.74
Density
x
N(0,9)
N(2,9)
Density plots of B(1,1) and B(2,1) − θ = 0.66
Density
x
B(1,1)
B(2,1)
Density plots of Exp(1) and Exp(0.5) − θ = 2/3
Density
x
Exp(0.5)
Exp(1)
