# Supplementary material for: A New Approach to the Nonparametric Behrens–Fisher Problem With Compatible Confidence Intervals
Source: Biom J. 2025 Nov 9;67(6):e70096. doi: 10.1002/bimj.70096 (PMC12598137; doi:10.1002/bimj.70096)
Supplement: Supplementary file 1 — Supporting information [file BIMJ-67-e70096-s002.zip › Schüürhuis_et_al_code_R2/R Code Submission/README.html]

Code Documentation


## Contents

- 1 Content
- 2 File and Folder-Structure
- 3 Main functionality
  - 3.1 `getTestResults.R`
  - 3.2 `getStudentizedPermutationTest.R`
  - 3.3 `util.R`
- 4 How to reproduce the results shown in the paper
  - 4.1 All results
  - 4.2 Exemplary results
- 5 Session Info
  - 5.1 Local
  - 5.2 Cluster

# Code Documentation

 Code

- Show All Code
- Hide All Code
- ---
- View Source

A New Approach to the Nonparametric Behrens-Fisher Problem with Compatible Confidence Intervals

Author

Stephen Schueuerhuis

## 1 Content

This files contains a short documentation of the code belonging to the submitted paper ***A New Approach to the Nonparametric Behrens-Fisher Problem with Compatible Confidence Intervals*** and illustrates how the results shown in the paper may be reproduced.

To begin working with the submitted code, open the R project file `nonparTests.Rproj`. This ensures that all file paths are correctly set relative to the project root.

## 2 File and Folder-Structure

The project contains the following files and folders:

File and Folder-Structure


| Name | Description |
| --- | --- |
| `functions` | Folder containing the main function `getTestResults()`, `getStudentizedPermutationTest()` as well as helper functions in `util.R` |
| `results` | Folder containing result files `df_t1e.csv` and `df_pwr_cov.csv` |
| `plots` | Folder containing the plots shown in the paper |
| `settings.R` | `R`-script creating `tibble`s containing the simulation scenarios |
| `simulation_main.R` | `R`-script performing the simulation and creating the files `df_t1e.csv` and `df_pwr_cov.csv`. To do that, the simulation functions `simulateT1E()` and `simulatePWRCOV()` from `util.R`, which are wrapper functions around `getTestResults()` are used |
| `plots.R` | `R`-script importing `df_t1e.csv` and `df_pwr_cov.csv` and creating the plots shown in the paper |
| `examples.R` | `R`-script creating the boxplot and computing the results for the data examples (Section 2 and 6 in the paper, data from Table 2), see 4.1 in the documentation |
| `simulation_repro_example.R` | Script reproducing results for some simulation settings, see 4.2 in the documentation |

## 3 Main functionality

### 3.1 `getTestResults.R`

This function compares two samples represented by matrices `X` and `Y`. It loops over each column (i.e., sample) and applies the Brunner-Munzel test and the newly proposed \(C^2\) test. If `permutation = TRUE`, a studentized permutation version of the Brunner-Munzel test is also performed. The results for each sample are stacked vertically into a `data.table`, with the `sample` column indicating which dataset each row of results belongs to. This function constitutes the core of the simulation study and is called repeatedly across all simulation settings.

Function Inputs


| Name | Description |
| --- | --- |
| `X` | An \(n \times d\) matrix containing the first sample, where each column \(i=1,\dots,d\) represents an independent dataset. |
| `Y` | An \(n \times d\) matrix containing the second sample, structured similarly to `X`. |
| `alpha` | Significance level used for hypothesis testing. |
| `permutation` | Logical (`TRUE`/`FALSE`). If `TRUE`, a studentized permutation test is performed. |
| `nperm` | Integer specifying the number of permutations (used only if `permutation = TRUE`). |
| `seed` | Integer value used to set the random seed for reproducibility. |

Function Outputs


| Name | Description |
| --- | --- |
| `test` | A character string indicating the name of the test to be performed. |
| `lower` | Lower bound of the confidence interval. |
| `theta` | Estimated Mann–Whitney effect size. |
| `upper` | Upper bound of the confidence interval. |
| `variance` | Estimated variance of `theta`. |
| `ties` | Estimated probability of ties between groups. |
| `statistic` | Test statistic value for the selected test. |
| `pval` | P-value associated with the test. |
| `rejH0` | Logical; `TRUE` if the null hypothesis is rejected (i.e., `pval < alpha`). |
| `separation` | Logical; `TRUE` if the samples are separated. |
| `sample` | Identifier for the dataset (column) the results correspond to. |

#### 3.1.1 **Example function call:**

Code

```
set.seed(28072025)
X <- matrix(rnorm(15*2), ncol = 2, nrow = 15)
Y <- matrix(rnorm(15*2, mean = 1), ncol = 2, nrow = 15)
getTestResults(X = X, Y = Y, alpha = 0.05, permutation = TRUE, nperm = 1e4, seed = 9072925)
```

```
                           test     lower     theta     upper  variance ties
1:                      BM-Test 0.6928175 0.8488889 1.0049602 0.1738836    0
2:                      BM-Test 0.5511224 0.7466667 0.9422110 0.2671746    0
3:                      C2-Test 0.6549900 0.8488889 0.9432555 0.1666697    0
4:                      C2-Test 0.5386136 0.7466667 0.8815366 0.2573061    0
5: Studentized Permutation test 0.6916653 0.8488889 1.0099338        NA   NA
6: Studentized Permutation test 0.5517728 0.7466667 0.9440772        NA   NA
   statistic         pval rejH0 separation   sample
1:  4.582669 8.983773e-05  TRUE      FALSE Sample 1
2:  2.613807 1.573948e-02  TRUE      FALSE Sample 2
3: 11.242066 7.996408e-04  TRUE      FALSE Sample 1
4:  5.367489 2.051549e-02  TRUE      FALSE Sample 2
5:  4.582669 2.000000e-04  TRUE      FALSE Sample 1
6:  2.613807 1.740000e-02  TRUE      FALSE Sample 2
```

### 3.2 `getStudentizedPermutationTest.R`

This function is called by the `getTestResults()` function and performs the studentized permutation test proposed by Neubert and Brunner (2007), using `nperm` permutations. The results from this test are then combined with the results of other tests returned by `getTestResults()`. The seed parameter ensures reproducibility, which is required due to the randomness introduced by the permutation procedure. Some parameters are used as input and written into the output to ensure consistent formatting with the other tests from `getTestResults()`.

Function Inputs


| Argument | Description |
| --- | --- |
| `x` | An n × 1 vector containing the first sample. |
| `y` | An n × 1 vector containing the second sample. |
| `nperm` | Number of permutations (used only if `permutation = TRUE`). |
| `alpha` | Significance level for hypothesis testing. |
| `seed` | Random seed for reproducibility. |

Function Outputs


| Result | Description |
| --- | --- |
| `Tn_BM` | Test statistic from the Brunner-Munzel test. |
| `theta` | Estimated Mann–Whitney effect size. |
| `se` | Estimated standard error (DeLong variance estimator). |
| `test` | Name of the test performed. |
| `lower` | Lower bound of the confidence interval. |
| `upper` | Upper bound of the confidence interval. |
| `variance` | Estimated variance of `theta`. |
| `ties` | Estimated probability of ties between groups. |
| `statistic` | Test statistic value for the selected test. |
| `pval` | P-value of the test. |
| `rejH0` | `TRUE` if null hypothesis is rejected (`pval < alpha`). |
| `separation` | `TRUE` if the samples are separated. |

### 3.3 `util.R`

The script `util.R` contains several helper functions, including functions for simulating data matrices according to a target distribution, conducting the simulations presented in the paper, and plotting the results. For details on these functions, we refer the reader to the comments within the corresponding `R`-script

## 4 How to reproduce the results shown in the paper

### 4.1 All results

To reproduce the results from the simulation study, start by opening the project file `nonparTests.Rproj`, followed by the script `simulation_main.R`. At the top of this script, necessary functions are sourced from `util.R`, `getStudentizedPermutationTest.R`, and `getTestResults.R`. It also runs `settings.R`, which generates two data frames—`df_t1e` and `df_pwr_cov`—containing the 840 and 153 parameter settings used in the simulation study for type-1 error and power/coverage, respectively. The script then calls the functions `simulateT1E.R` and `simulatePWRCOV.R` (defined in `util.R`) to iterate over `df_t1e` and `df_pwr_cov` in a for-loop, conducting the simulations. Once all settings have been processed, the results are saved in the `results` folder as `df_t1e.csv` and `df_pwr_cov.csv`. The files are already located in the corresponding folders. To generate the corresponding plots, open and run the script `plots.R`. This will produce all figures illustrating the simulation results and save them in the `plots/main paper` and `plots/supplement` directories. The files will be named according to their appearance in the paper in `.eps` format. `.pdf`-Versions of the plots are also located in the corresponding folder. Thus, running `simulation_main.R` followed by `plots.R` will reproduce all the results and figures presented in the paper. For full reproducibility, each of the data frames containing the simulation settings includes a column specifying the random seeds used in the study. These seeds were randomly generated but are fixed, ensuring that re-running the code will yield identical results.

To reproduce the results of the example in **Section 2** and **Section 6** of the paper as well as the results for the data in **Table 2**, run the file `examples.R`. Running the code will produce the boxplot next to Table 1 (page 4) and the results depicted in Table 5 (page 19-20). Moreover, the results for the Table 2 cited on page 8 (lines 196-199) and page 11 (Remark 4.2, point 1) are computed.

### 4.2 Exemplary results

Due to the extensive runtime required for 100,000 simulation runs, particularly for the computationally intensive studentized permutation test, the simulation study was conducted on a high-performance computing cluster. In correspondence with the *Checklist for Code and Data Supplements*, we provide the intermediate results of our simulation study for type-1 error rate, power and coverage in the `results` folder as `.csv`-files. Running the `plots.R` scripts will produces the figures shown in the main paper and the supplement. Moreover, to avoid rerunning the entire simulation study, we created a script named `simulation_repro_example.R`. This script should replicate the simulation for some specific settings and compares the results to those stored in the corresponding `.csv` files for these settings.

## 5 Session Info

### 5.1 Local

The example results presented in this paper that were run on a local computer. Specifically the example boxplot on page 4 and the results in Table 5 (page 19) as well as the results for the data in Table 2, were generated using the following R packages and their corresponding versions:

Code

```
sessionInfo()
```

```
R version 4.1.2 (2021-11-01)
Platform: x86_64-w64-mingw32/x64 (64-bit)
Running under: Windows 10 x64 (build 19045)

Matrix products: default

locale:
[1] LC_COLLATE=German_Germany.1252  LC_CTYPE=German_Germany.1252   
[3] LC_MONETARY=German_Germany.1252 LC_NUMERIC=C                   
[5] LC_TIME=German_Germany.1252    

attached base packages:
[1] splines   stats4    stats     graphics  grDevices utils     datasets 
[8] methods   base     

other attached packages:
 [1] ggpubr_0.4.0       data.table_1.14.2  VGAM_1.1-8         MASS_7.3-57       
 [5] matrixStats_0.62.0 forcats_1.0.0      stringr_1.4.0      dplyr_1.1.0       
 [9] purrr_0.3.4        readr_2.1.2        tidyr_1.2.0        tibble_3.1.7      
[13] ggplot2_3.4.1      tidyverse_1.3.2   

loaded via a namespace (and not attached):
 [1] lubridate_1.8.0     assertthat_0.2.1    digest_0.6.31      
 [4] utf8_1.2.2          R6_2.5.1            cellranger_1.1.0   
 [7] backports_1.4.1     reprex_2.0.1        evaluate_0.22      
[10] httr_1.4.3          pillar_1.9.0        rlang_1.1.3        
[13] googlesheets4_1.0.0 readxl_1.4.0        rstudioapi_0.13    
[16] car_3.0-13          rmarkdown_2.14      googledrive_2.0.0  
[19] htmlwidgets_1.6.2   munsell_0.5.0       broom_1.0.3        
[22] compiler_4.1.2      modelr_0.1.8        xfun_0.39          
[25] pkgconfig_2.0.3     htmltools_0.5.5     tidyselect_1.2.0   
[28] fansi_1.0.3         crayon_1.5.1        tzdb_0.3.0         
[31] dbplyr_2.1.1        withr_2.5.0         grid_4.1.2         
[34] jsonlite_1.8.4      gtable_0.3.0        lifecycle_1.0.3    
[37] DBI_1.1.2           magrittr_2.0.3      scales_1.2.1       
[40] carData_3.0-5       cli_3.3.0           stringi_1.7.6      
[43] ggsignif_0.6.3      fs_1.6.3            xml2_1.3.3         
[46] ellipsis_0.3.2      generics_0.1.2      vctrs_0.6.1        
[49] tools_4.1.2         glue_1.6.2          hms_1.1.1          
[52] abind_1.4-5         fastmap_1.1.0       yaml_2.3.5         
[55] colorspace_2.0-3    gargle_1.2.0        rstatix_0.7.0      
[58] rvest_1.0.2         knitr_1.39          haven_2.5.0
```

### 5.2 Cluster

The simulation study was conducted on a high-performance computing cluster to mitigate the extensive runtime. The specifications of the R packages used in the code and installed on the cluster are as follows:

- R-Version: r-base=4.4.2=h64c9cd0\_0
- Tidyverse: r-tidyverse=2.0.0=r44h785f33e\_2
- matrixStats: r-matrixstats=1.4.1=r44h2b5f3a1\_0
- MASS: r-mass=7.3\_60.0.1=r44hb1dbf0f\_1
- VGAM: r-vgam=1.1\_12=r44hb67ce94\_0
- data.table: r-data.table=1.15.4=r44h5f06984\_1
- ggpubr: r-ggpubr=0.6.0=r44hc72bb7e\_2

The complete cluster specification is provided in the attached file `environment.yml` .

To avoid rerunning the entire simulation study, we created a script named `simulation_repro_example.R`. Running this script with the R Version and packages above should replicate the results for the simulation study in those specific settings.


##### Source Code

```
---
title: "Code Documentation"
subtitle: "A New Approach to the Nonparametric Behrens-Fisher Problem with Compatible Confidence Intervals"
author: 
  - name: "Stephen Schueuerhuis"
format:
  html:
    toc: true
    toc-depth: 3
    toc-location: right
    toc-title: "Contents"
    number-sections: true
    smooth-scroll: true
    theme: cosmo  # You can change to flatly, journal, simplex, etc.
    code-fold: true
    code-tools: true
    highlight-style: github
editor: visual
---

```{r message=FALSE, warning=FALSE}
#| echo: false
#| results: false
source("functions/util.R")
source("functions/getTestResults.R")
source("functions/getStudentizedPermutationTest.R")
source("settings.R")

```

## Content

This files contains a short documentation of the code belonging to the submitted paper ***A New Approach to the Nonparametric Behrens-Fisher Problem with Compatible Confidence Intervals*** and illustrates how the results shown in the paper may be reproduced.

To begin working with the submitted code, open the R project file `nonparTests.Rproj`. This ensures that all file paths are correctly set relative to the project root.

## File and Folder-Structure

The project contains the following files and folders:

| Name                         | Description                                                                                                                                                                                                                                              |
|-------------------|-----------------------------------------------------|
| `functions`                  | Folder containing the main function `getTestResults()`, `getStudentizedPermutationTest()` as well as helper functions in `util.R`                                                                                                                        |
| `results`                    | Folder containing result files `df_t1e.csv` and `df_pwr_cov.csv`                                                                                                                                                                                         |
| `plots`                      | Folder containing the plots shown in the paper                                                                                                                                                                                                           |
| `settings.R`                 | `R`-script creating `tibble`s containing the simulation scenarios                                                                                                                                                                                        |
| `simulation_main.R`          | `R`-script performing the simulation and creating the files `df_t1e.csv` and `df_pwr_cov.csv`. To do that, the simulation functions `simulateT1E()` and `simulatePWRCOV()` from `util.R`, which are wrapper functions around `getTestResults()` are used |
| `plots.R`                    | `R`-script importing `df_t1e.csv` and `df_pwr_cov.csv` and creating the plots shown in the paper                                                                                                                                                         |
| `examples.R`                 | `R`-script creating the boxplot and computing the results for the data examples (Section 2 and 6 in the paper, data from Table 2), see 4.1 in the documentation                                                                                          |
| `simulation_repro_example.R` | Script reproducing results for some simulation settings, see 4.2 in the documentation                                                                                                                                                                    |

: File and Folder-Structure {tbl-colwidths="\[20,80\]"}

## Main functionality

### `getTestResults.R`

This function compares two samples represented by matrices `X` and `Y`. It loops over each column (i.e., sample) and applies the Brunner-Munzel test and the newly proposed $C^2$ test. If `permutation = TRUE`, a studentized permutation version of the Brunner-Munzel test is also performed. The results for each sample are stacked vertically into a `data.table`, with the `sample` column indicating which dataset each row of results belongs to. This function constitutes the core of the simulation study and is called repeatedly across all simulation settings.

| Name          | Description                                                                                                            |
|-------------------|-----------------------------------------------------|
| `X`           | An $n \times d$ matrix containing the first sample, where each column $i=1,\dots,d$ represents an independent dataset. |
| `Y`           | An $n \times d$ matrix containing the second sample, structured similarly to `X`.                                      |
| `alpha`       | Significance level used for hypothesis testing.                                                                        |
| `permutation` | Logical (`TRUE`/`FALSE`). If `TRUE`, a studentized permutation test is performed.                                      |
| `nperm`       | Integer specifying the number of permutations (used only if `permutation = TRUE`).                                     |
| `seed`        | Integer value used to set the random seed for reproducibility.                                                         |

: Function Inputs {tbl-colwidths="\[25,75\]"}

| Name         | Description                                                                |
|-------------------|-----------------------------------------------------|
| `test`       | A character string indicating the name of the test to be performed.        |
| `lower`      | Lower bound of the confidence interval.                                    |
| `theta`      | Estimated Mann--Whitney effect size.                                       |
| `upper`      | Upper bound of the confidence interval.                                    |
| `variance`   | Estimated variance of `theta`.                                             |
| `ties`       | Estimated probability of ties between groups.                              |
| `statistic`  | Test statistic value for the selected test.                                |
| `pval`       | P-value associated with the test.                                          |
| `rejH0`      | Logical; `TRUE` if the null hypothesis is rejected (i.e., `pval < alpha`). |
| `separation` | Logical; `TRUE` if the samples are separated.                              |
| `sample`     | Identifier for the dataset (column) the results correspond to.             |

: Function Outputs {tbl-colwidths="\[25,75\]"}

#### **Example function call:**

```{r}
set.seed(28072025)
X <- matrix(rnorm(15*2), ncol = 2, nrow = 15)
Y <- matrix(rnorm(15*2, mean = 1), ncol = 2, nrow = 15)
getTestResults(X = X, Y = Y, alpha = 0.05, permutation = TRUE, nperm = 1e4, seed = 9072925) 

```

### `getStudentizedPermutationTest.R`

This function is called by the `getTestResults()` function and performs the studentized permutation test proposed by Neubert and Brunner (2007), using `nperm` permutations. The results from this test are then combined with the results of other tests returned by `getTestResults()`. The seed parameter ensures reproducibility, which is required due to the randomness introduced by the permutation procedure. Some parameters are used as input and written into the output to ensure consistent formatting with the other tests from `getTestResults()`.

| Argument | Description                                                 |
|----------|-------------------------------------------------------------|
| `x`      | An n × 1 vector containing the first sample.                |
| `y`      | An n × 1 vector containing the second sample.               |
| `nperm`  | Number of permutations (used only if `permutation = TRUE`). |
| `alpha`  | Significance level for hypothesis testing.                  |
| `seed`   | Random seed for reproducibility.                            |

: Function Inputs {tbl-colwidths="\[25,75\]"}

| Result       | Description                                             |
|--------------|---------------------------------------------------------|
| `Tn_BM`      | Test statistic from the Brunner-Munzel test.            |
| `theta`      | Estimated Mann--Whitney effect size.                    |
| `se`         | Estimated standard error (DeLong variance estimator).   |
| `test`       | Name of the test performed.                             |
| `lower`      | Lower bound of the confidence interval.                 |
| `upper`      | Upper bound of the confidence interval.                 |
| `variance`   | Estimated variance of `theta`.                          |
| `ties`       | Estimated probability of ties between groups.           |
| `statistic`  | Test statistic value for the selected test.             |
| `pval`       | P-value of the test.                                    |
| `rejH0`      | `TRUE` if null hypothesis is rejected (`pval < alpha`). |
| `separation` | `TRUE` if the samples are separated.                    |

: Function Outputs {tbl-colwidths="\[25,75\]"}

### `util.R`

The script `util.R` contains several helper functions, including functions for simulating data matrices according to a target distribution, conducting the simulations presented in the paper, and plotting the results. For details on these functions, we refer the reader to the comments within the corresponding `R`-script

## How to reproduce the results shown in the paper

### All results

To reproduce the results from the simulation study, start by opening the project file `nonparTests.Rproj`, followed by the script `simulation_main.R`. At the top of this script, necessary functions are sourced from `util.R`, `getStudentizedPermutationTest.R`, and `getTestResults.R`. It also runs `settings.R`, which generates two data frames---`df_t1e` and `df_pwr_cov`---containing the 840 and 153 parameter settings used in the simulation study for type-1 error and power/coverage, respectively. The script then calls the functions `simulateT1E.R` and `simulatePWRCOV.R` (defined in `util.R`) to iterate over `df_t1e` and `df_pwr_cov` in a for-loop, conducting the simulations. Once all settings have been processed, the results are saved in the `results` folder as `df_t1e.csv` and `df_pwr_cov.csv`. The files are already located in the corresponding folders. To generate the corresponding plots, open and run the script `plots.R`. This will produce all figures illustrating the simulation results and save them in the `plots/main paper` and `plots/supplement` directories. The files will be named according to their appearance in the paper in `.eps` format. `.pdf`-Versions of the plots are also located in the corresponding folder. Thus, running `simulation_main.R` followed by `plots.R` will reproduce all the results and figures presented in the paper. For full reproducibility, each of the data frames containing the simulation settings includes a column specifying the random seeds used in the study. These seeds were randomly generated but are fixed, ensuring that re-running the code will yield identical results.

To reproduce the results of the example in **Section 2** and **Section 6** of the paper as well as the results for the data in **Table 2**, run the file `examples.R`. Running the code will produce the boxplot next to Table 1 (page 4) and the results depicted in Table 5 (page 19-20). Moreover, the results for the Table 2 cited on page 8 (lines 196-199) and page 11 (Remark 4.2, point 1) are computed.

### Exemplary results

Due to the extensive runtime required for 100,000 simulation runs, particularly for the computationally intensive studentized permutation test, the simulation study was conducted on a high-performance computing cluster. In correspondence with the *Checklist for Code and Data Supplements*, we provide the intermediate results of our simulation study for type-1 error rate, power and coverage in the `results` folder as `.csv`-files. Running the `plots.R` scripts will produces the figures shown in the main paper and the supplement. Moreover, to avoid rerunning the entire simulation study, we created a script named `simulation_repro_example.R`. This script should replicate the simulation for some specific settings and compares the results to those stored in the corresponding `.csv` files for these settings.

## Session Info

### Local

The example results presented in this paper that were run on a local computer. Specifically the example boxplot on page 4 and the results in Table 5 (page 19) as well as the results for the data in Table 2, were generated using the following R packages and their corresponding versions:

```{r}
sessionInfo()
```

### Cluster

The simulation study was conducted on a high-performance computing cluster to mitigate the extensive runtime. The specifications of the R packages used in the code and installed on the cluster are as follows:

-   R-Version: r-base=4.4.2=h64c9cd0_0

-   Tidyverse: r-tidyverse=2.0.0=r44h785f33e_2

-   matrixStats: r-matrixstats=1.4.1=r44h2b5f3a1_0

-   MASS: r-mass=7.3_60.0.1=r44hb1dbf0f_1

-   VGAM: r-vgam=1.1_12=r44hb67ce94_0

-   data.table: r-data.table=1.15.4=r44h5f06984_1

-   ggpubr: r-ggpubr=0.6.0=r44hc72bb7e_2

The complete cluster specification is provided in the attached file `environment.yml` .

To avoid rerunning the entire simulation study, we created a script named `simulation_repro_example.R`. Running this script with the R Version and packages above should replicate the results for the simulation study in those specific settings.
```
