# Supplementary figures and images for: A New Approach to the Nonparametric Behrens–Fisher Problem With Compatible Confidence Intervals
Source: Biom J. 2025 Nov 9;67(6):e70096. doi: 10.1002/bimj.70096 (PMC12598137; doi:10.1002/bimj.70096)

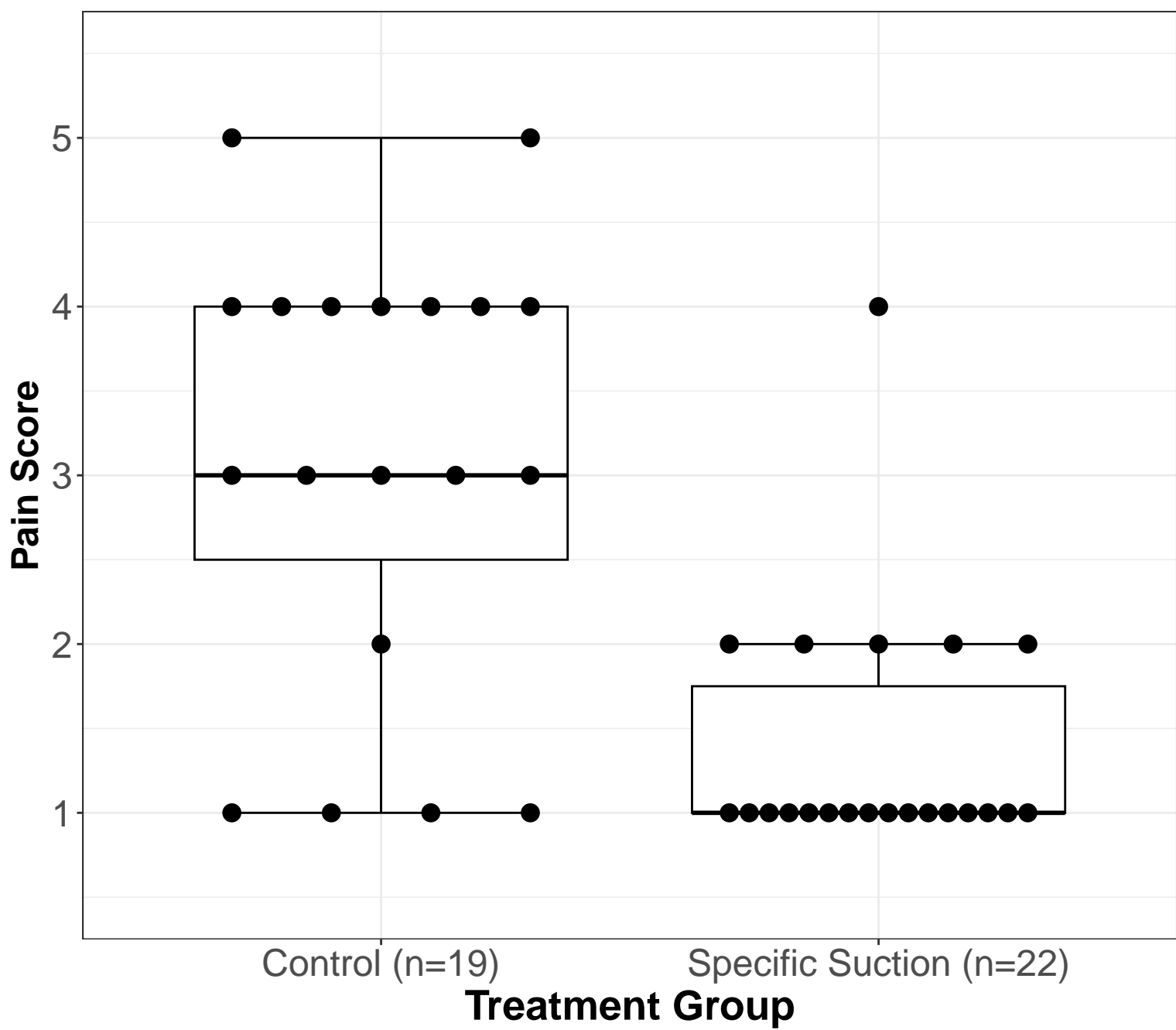

Supplement: Supplementary file 1 — Supporting information [file BIMJ-67-e70096-s002.zip › Schüürhuis_et_al_code_R2/R Code Submission/plots/Main Paper/Data_Table1.pdf]

$\alpha = 0.05$

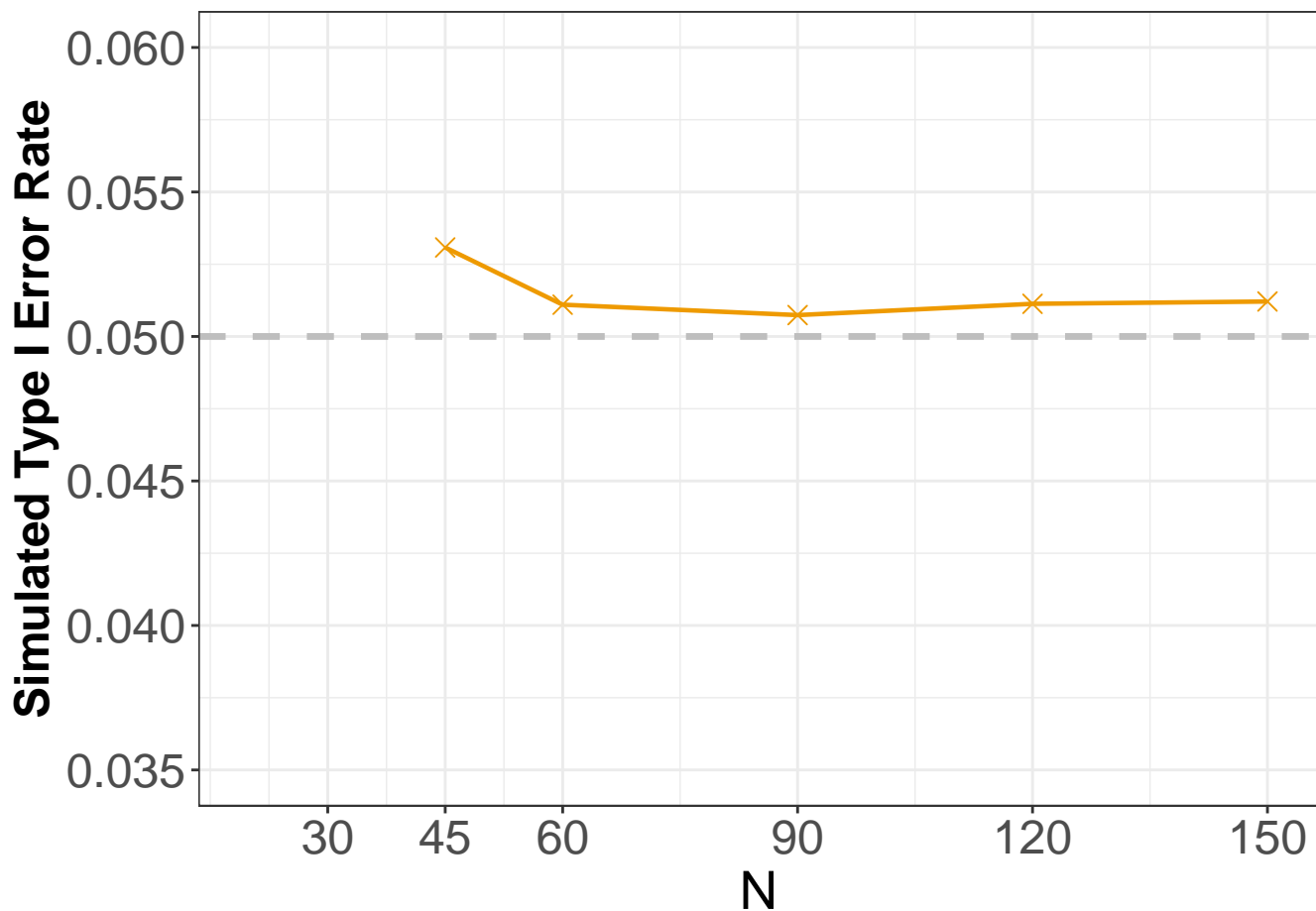

—x—  $T_N^{\text{BM}}$

$\alpha = 0.005$

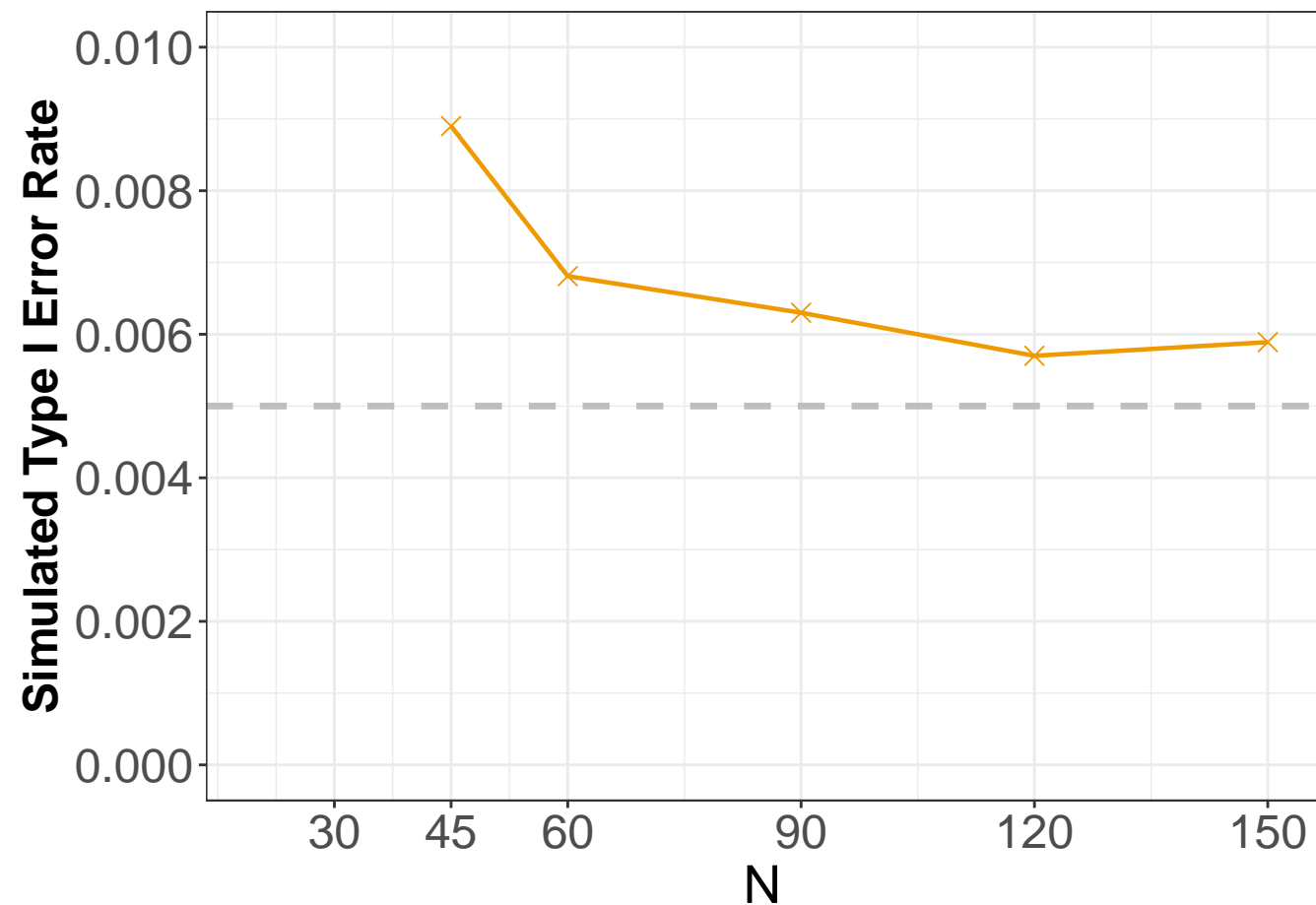

—x—  $T_N^{\text{BM}}$

Supplement: Supplementary file 1 — Supporting information [file BIMJ-67-e70096-s002.zip › Schüürhuis_et_al_code_R2/R Code Submission/plots/Main Paper/Figure1.pdf]

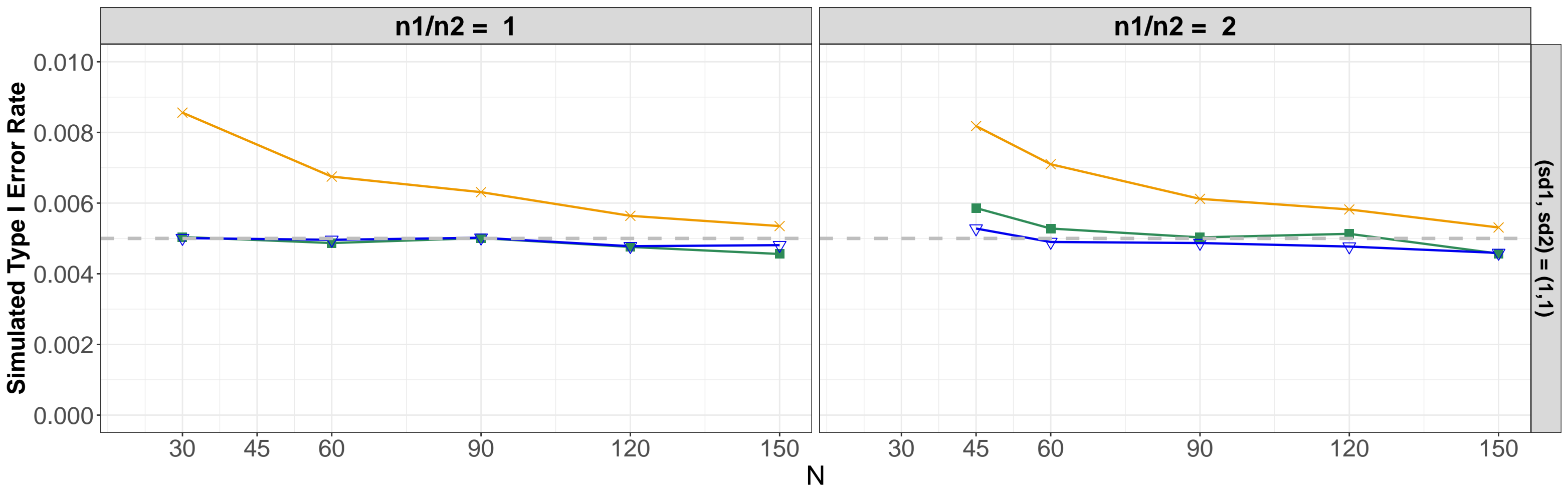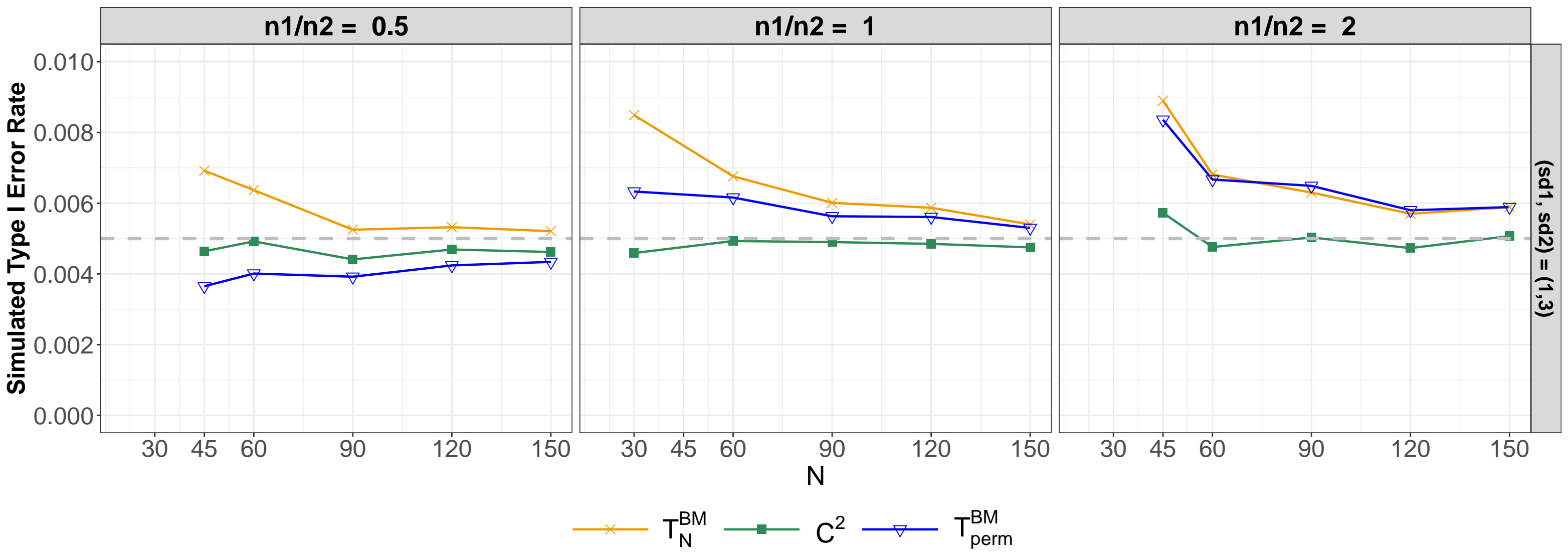

Supplement: Supplementary file 1 — Supporting information [file BIMJ-67-e70096-s002.zip › Schüürhuis_et_al_code_R2/R Code Submission/plots/Main Paper/Figure2.pdf]

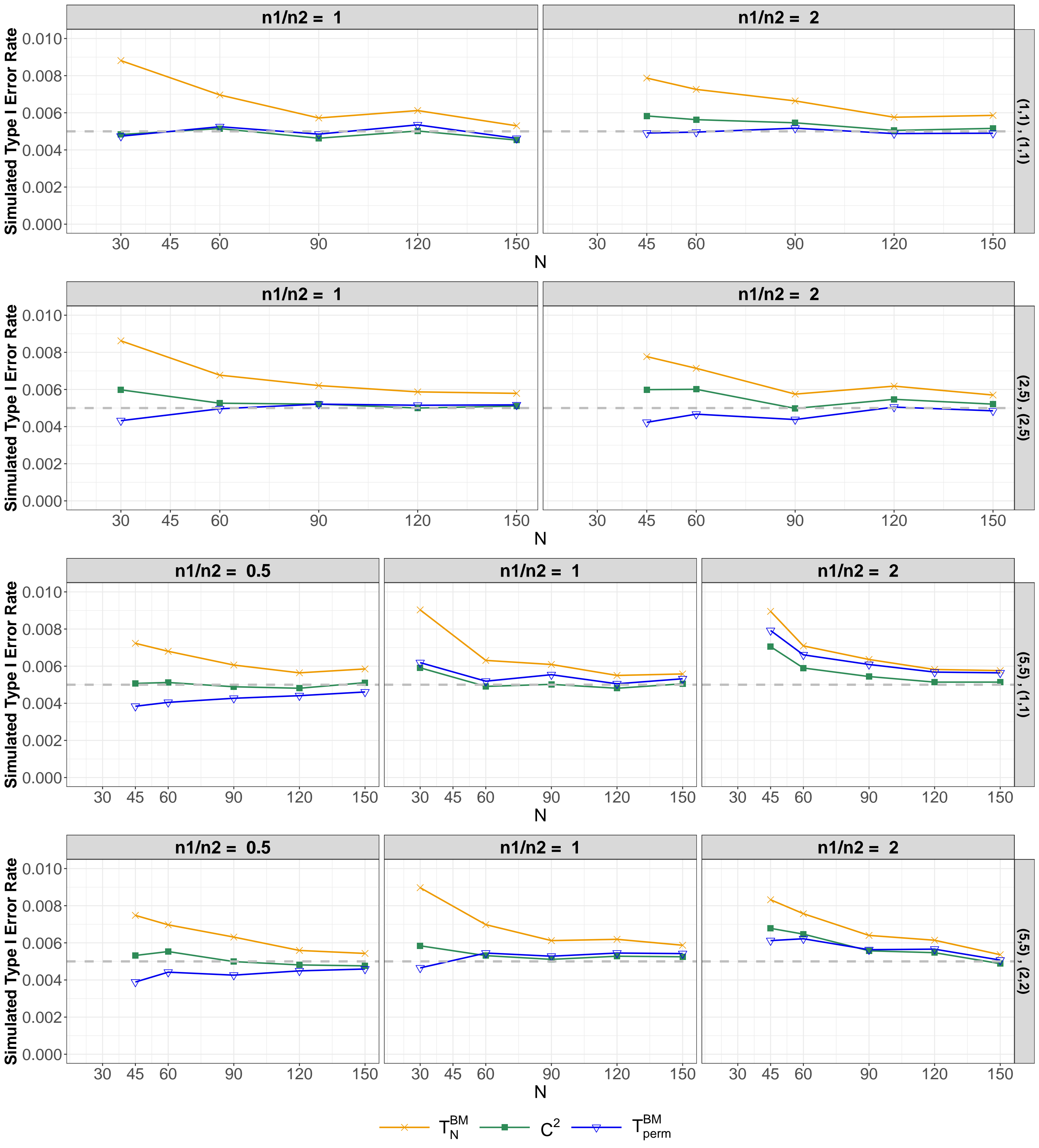

Supplement: Supplementary file 1 — Supporting information [file BIMJ-67-e70096-s002.zip › Schüürhuis_et_al_code_R2/R Code Submission/plots/Main Paper/Figure3.pdf]

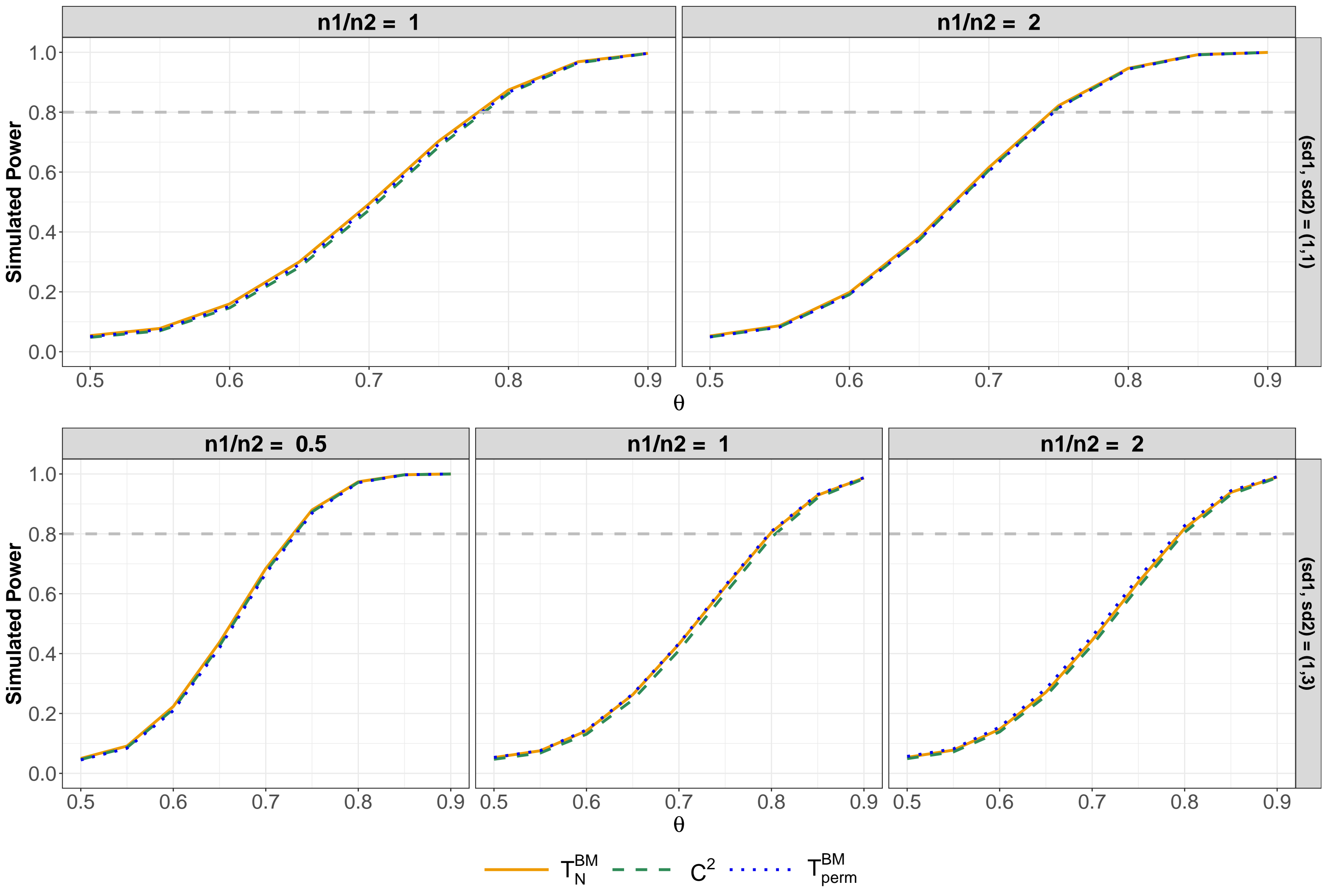

Supplement: Supplementary file 1 — Supporting information [file BIMJ-67-e70096-s002.zip › Schüürhuis_et_al_code_R2/R Code Submission/plots/Main Paper/Figure4.pdf]

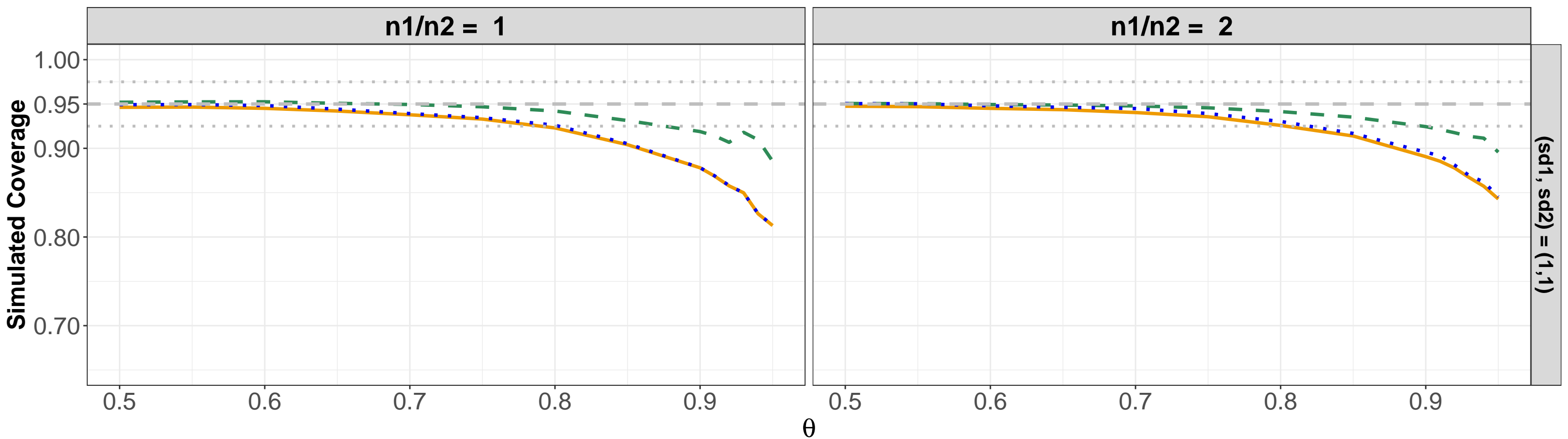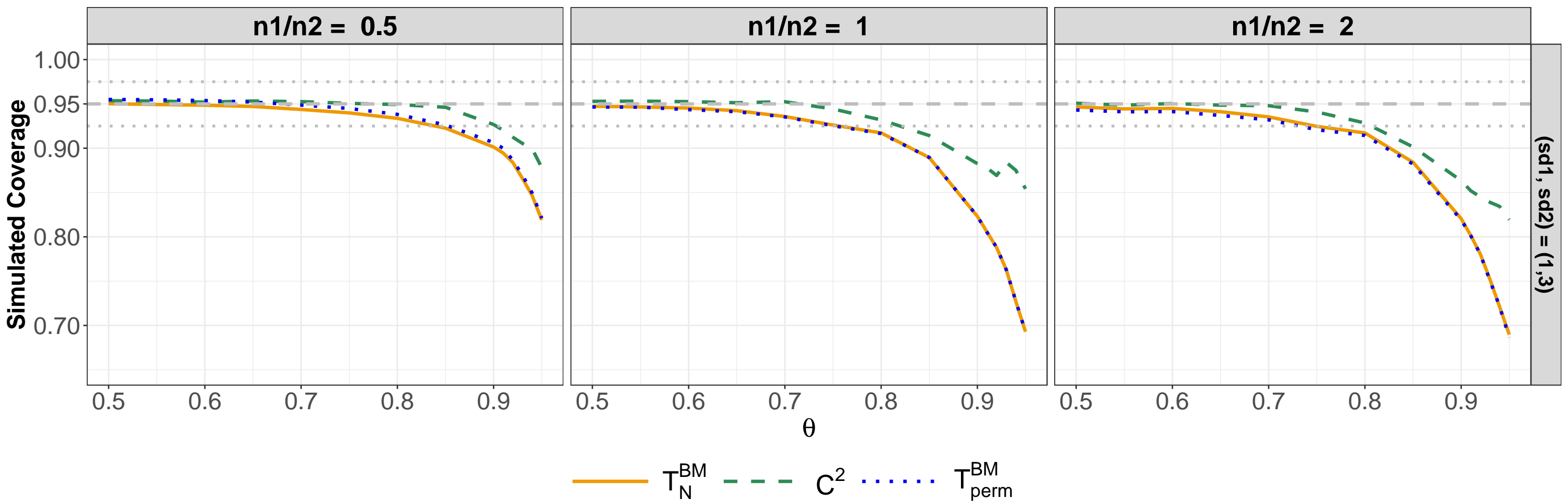

Supplement: Supplementary file 1 — Supporting information [file BIMJ-67-e70096-s002.zip › Schüürhuis_et_al_code_R2/R Code Submission/plots/Main Paper/Figure5.pdf]

Density plots of B(2,5)

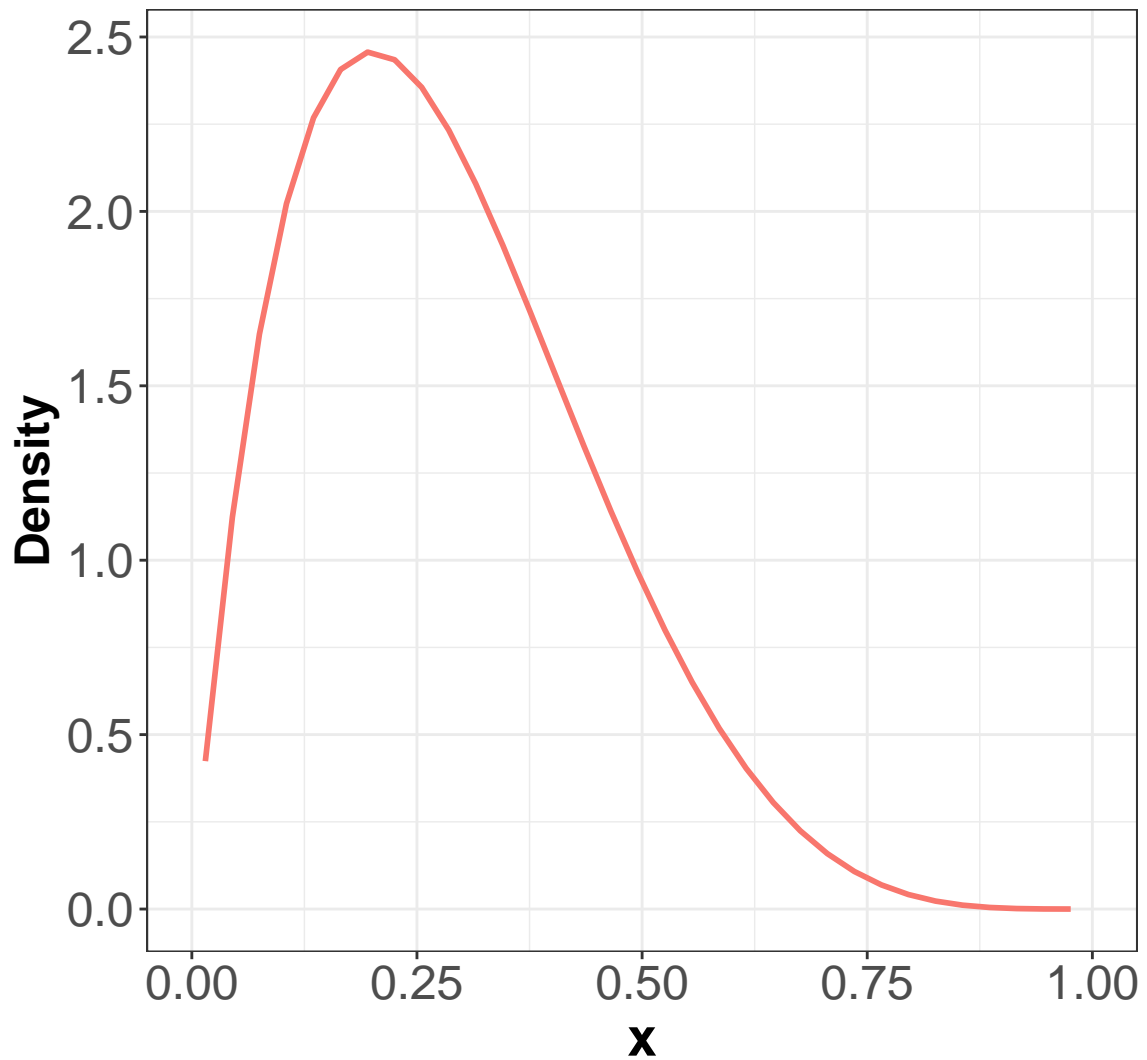

Density plots of Exp(1)

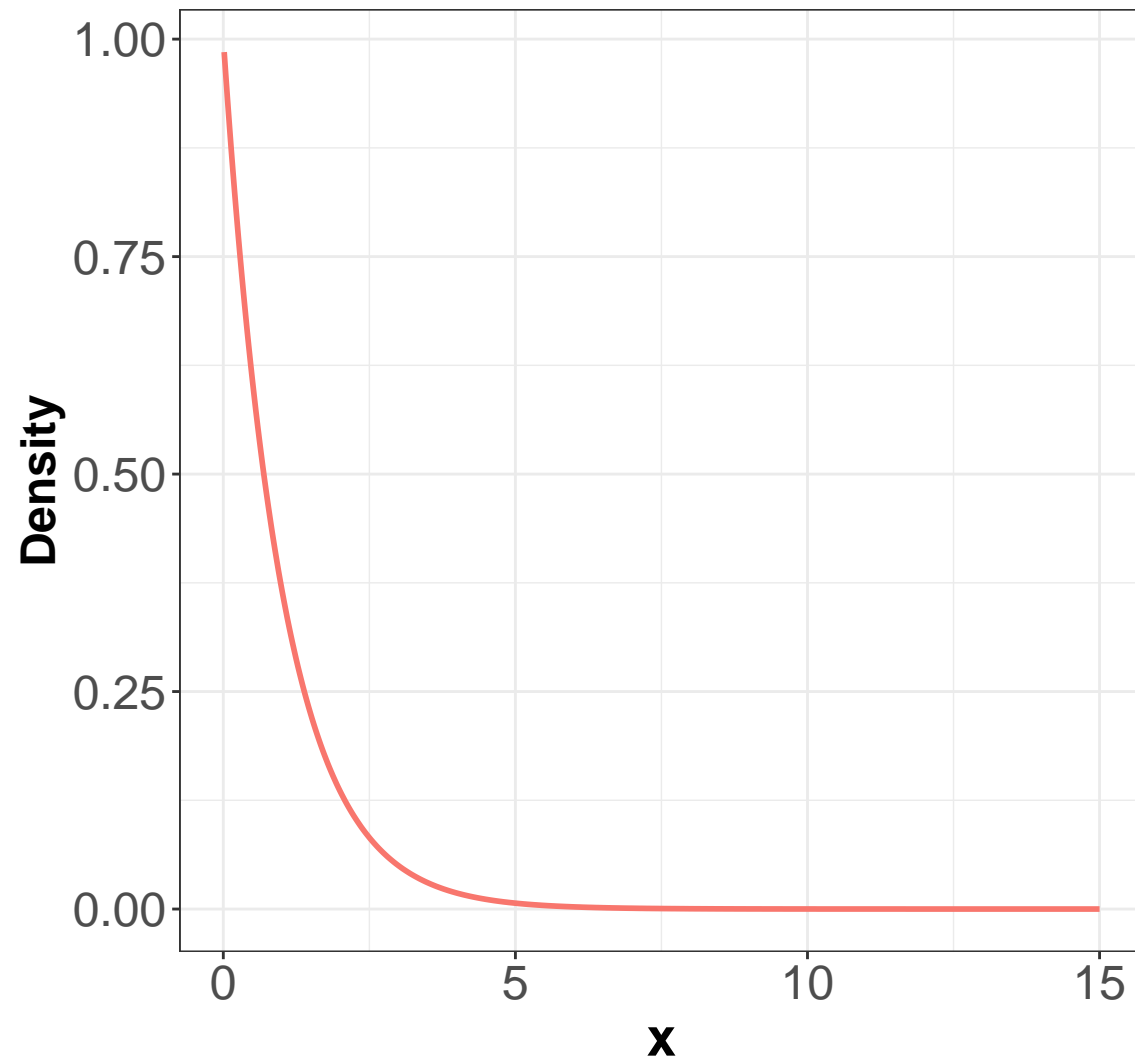

Density plots of Pois(1)

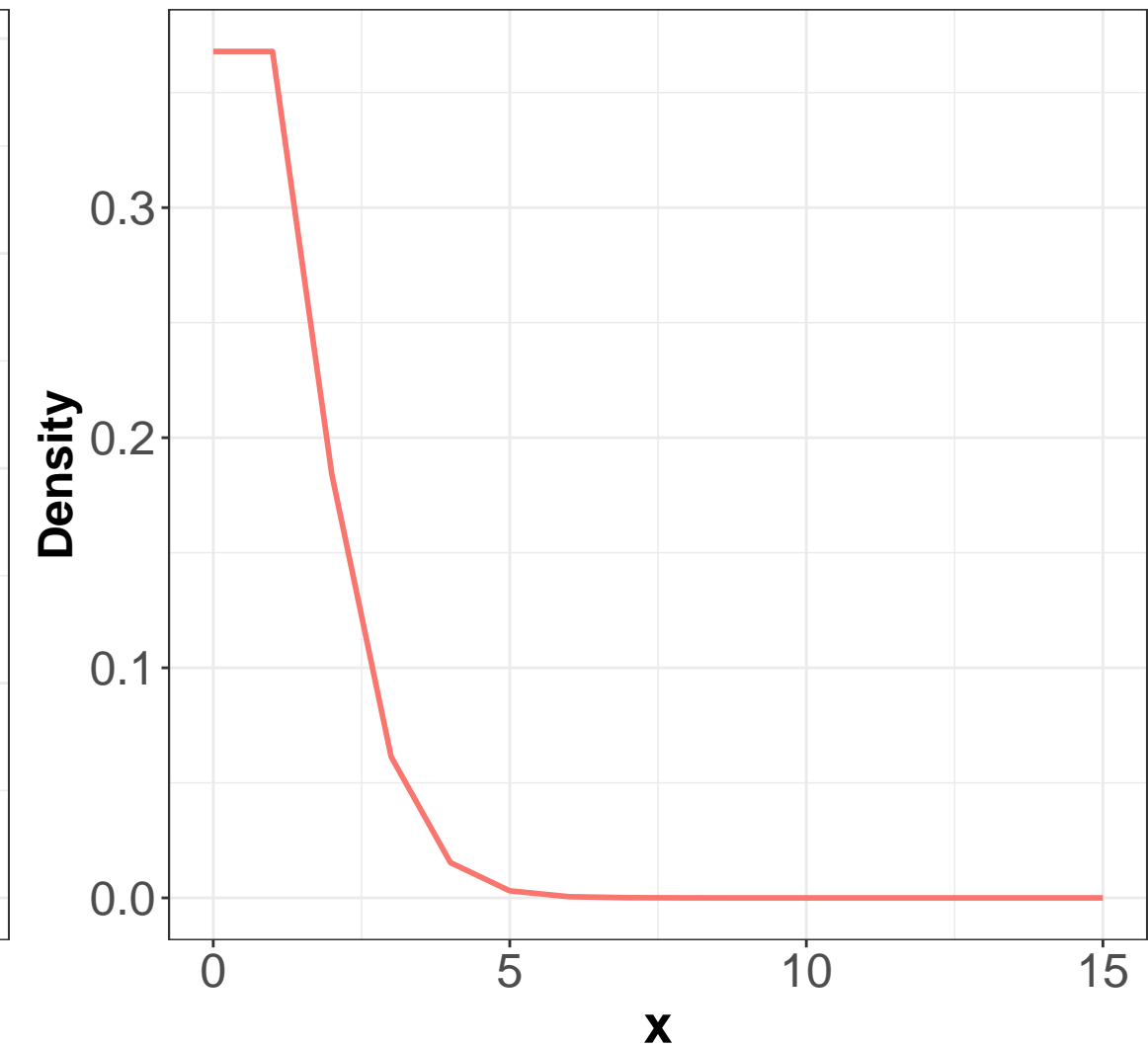

Supplement: Supplementary file 1 — Supporting information [file BIMJ-67-e70096-s002.zip › Schüürhuis_et_al_code_R2/R Code Submission/plots/Supplement/section1.1_table3-hom.pdf]

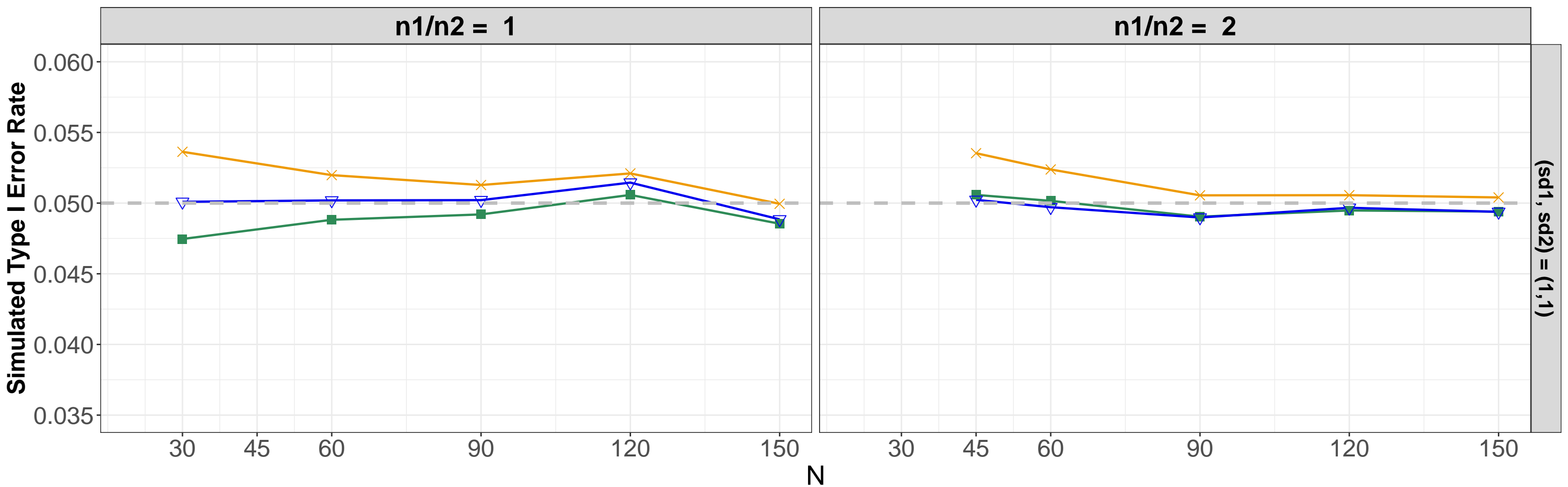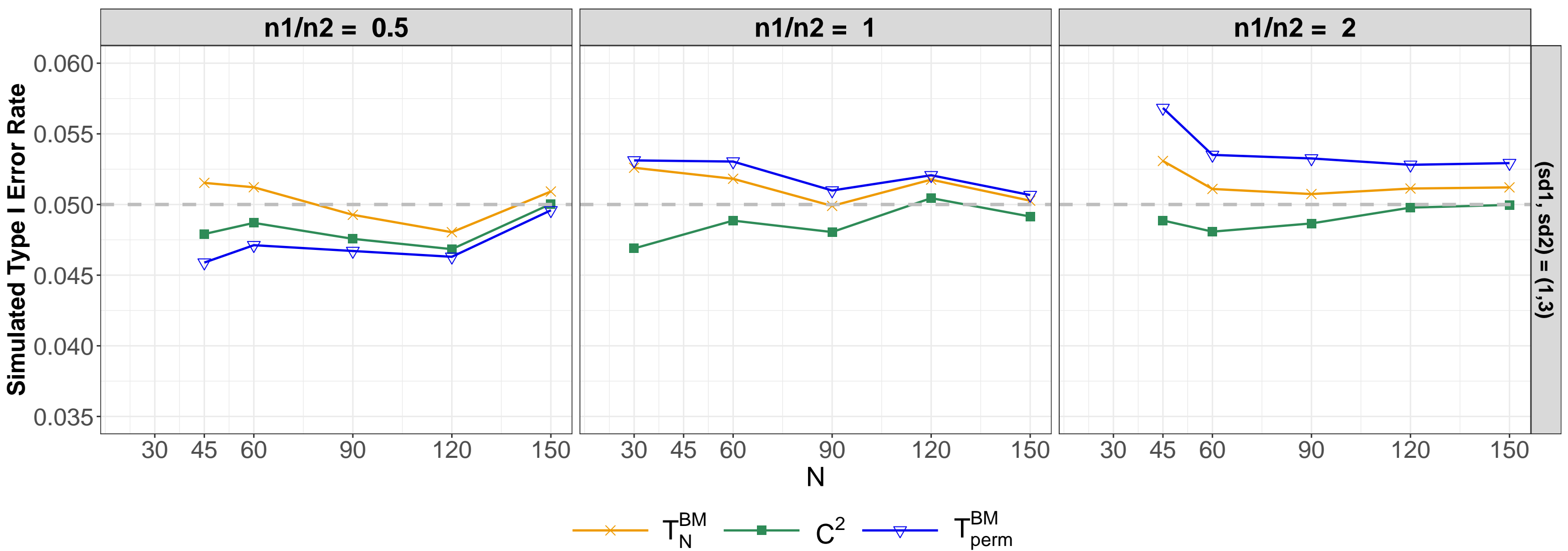

$T_N^{BM}$ 
 $C^2$ 
 $T_{perm}^{BM}$

Supplement: Supplementary file 1 — Supporting information [file BIMJ-67-e70096-s002.zip › Schüürhuis_et_al_code_R2/R Code Submission/plots/Supplement/section2.1.1_normal_t1e.pdf]

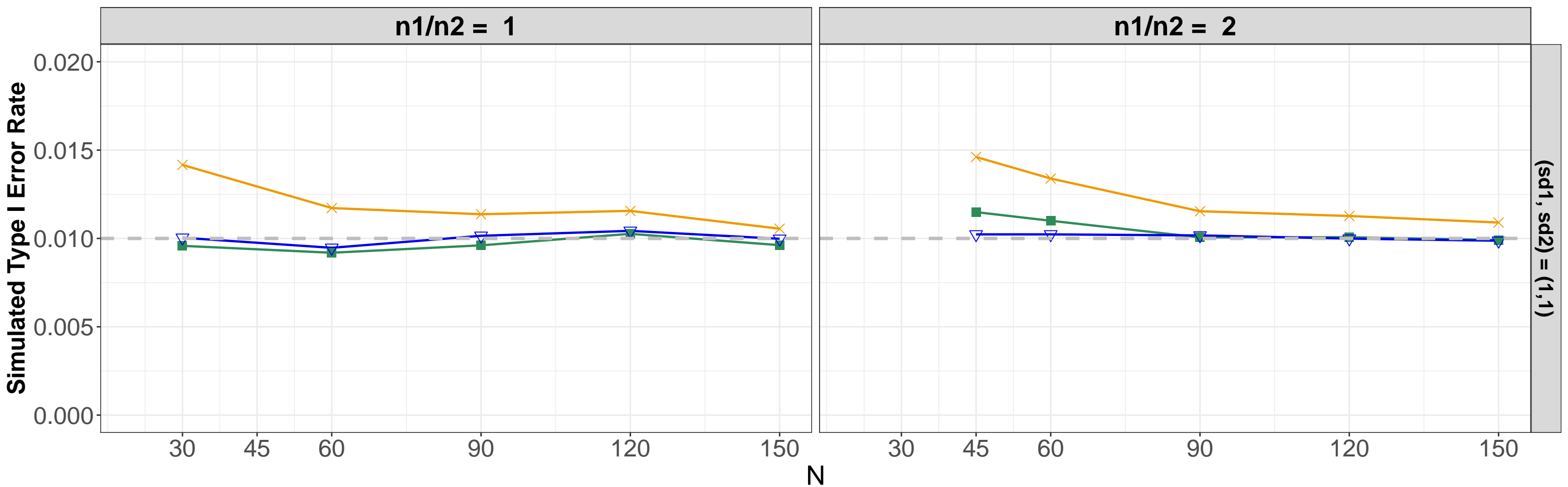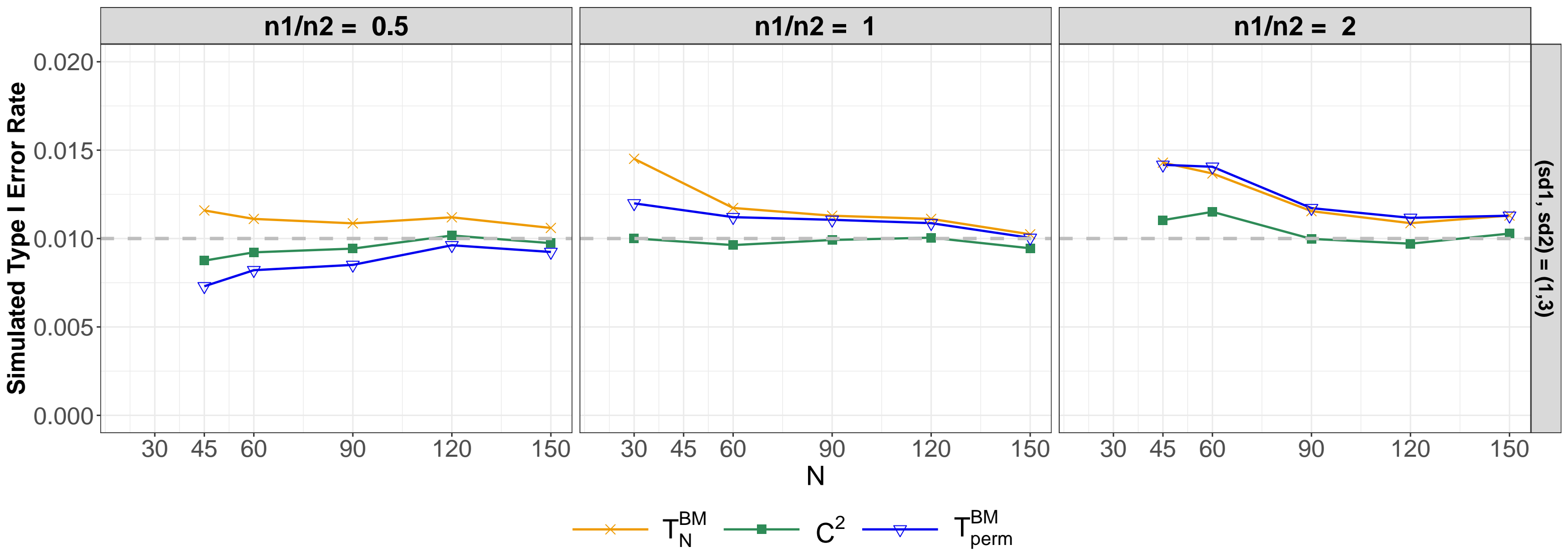

Supplement: Supplementary file 1 — Supporting information [file BIMJ-67-e70096-s002.zip › Schüürhuis_et_al_code_R2/R Code Submission/plots/Supplement/section2.1.2_normal_t1e.pdf]

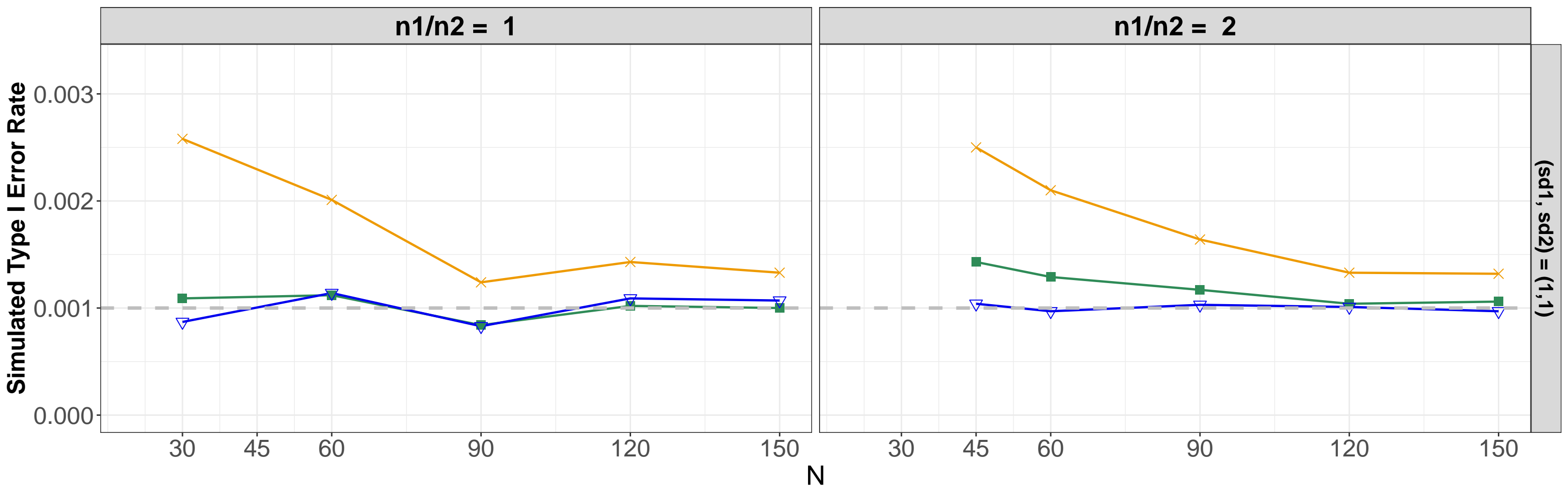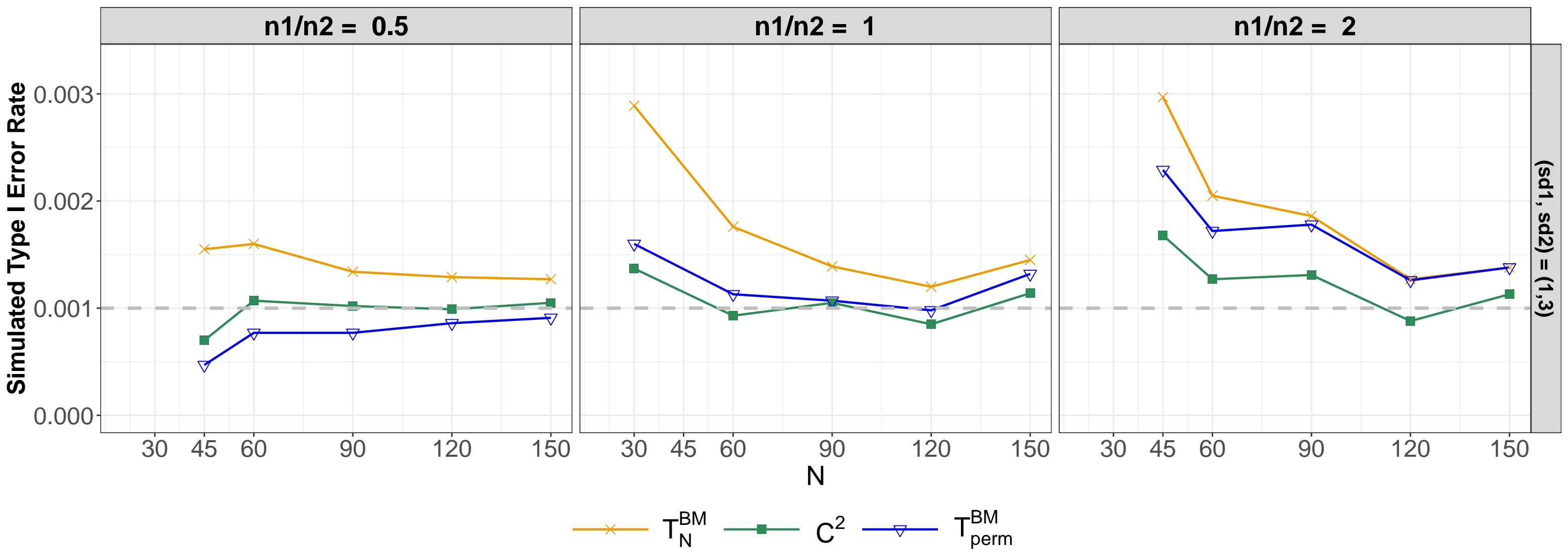

Supplement: Supplementary file 1 — Supporting information [file BIMJ-67-e70096-s002.zip › Schüürhuis_et_al_code_R2/R Code Submission/plots/Supplement/section2.1.4_normal_t1e.pdf]

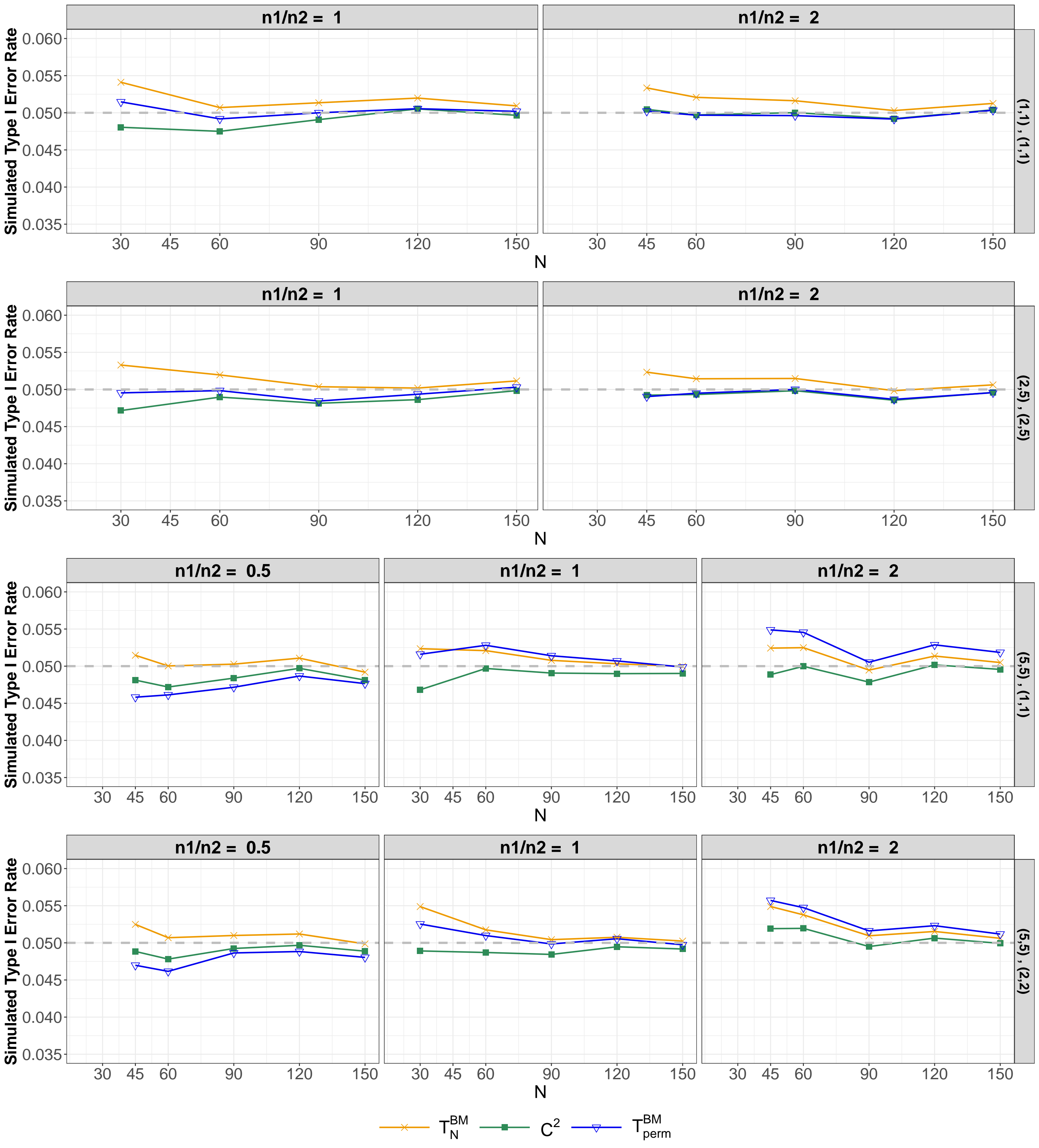

Supplement: Supplementary file 1 — Supporting information [file BIMJ-67-e70096-s002.zip › Schüürhuis_et_al_code_R2/R Code Submission/plots/Supplement/section2.2.1_beta_t1e.pdf]

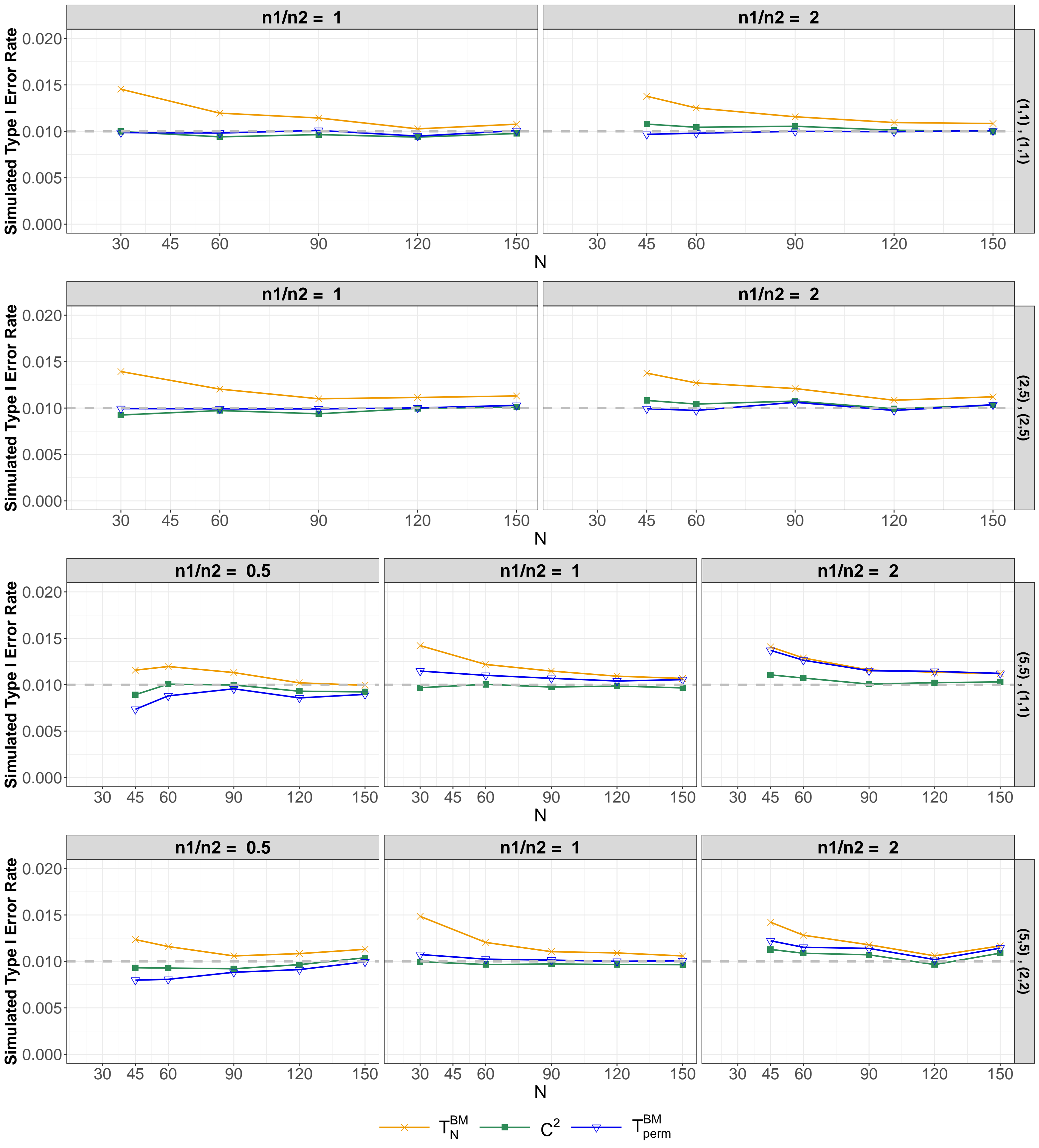

Supplement: Supplementary file 1 — Supporting information [file BIMJ-67-e70096-s002.zip › Schüürhuis_et_al_code_R2/R Code Submission/plots/Supplement/section2.2.2_beta_t1e.pdf]

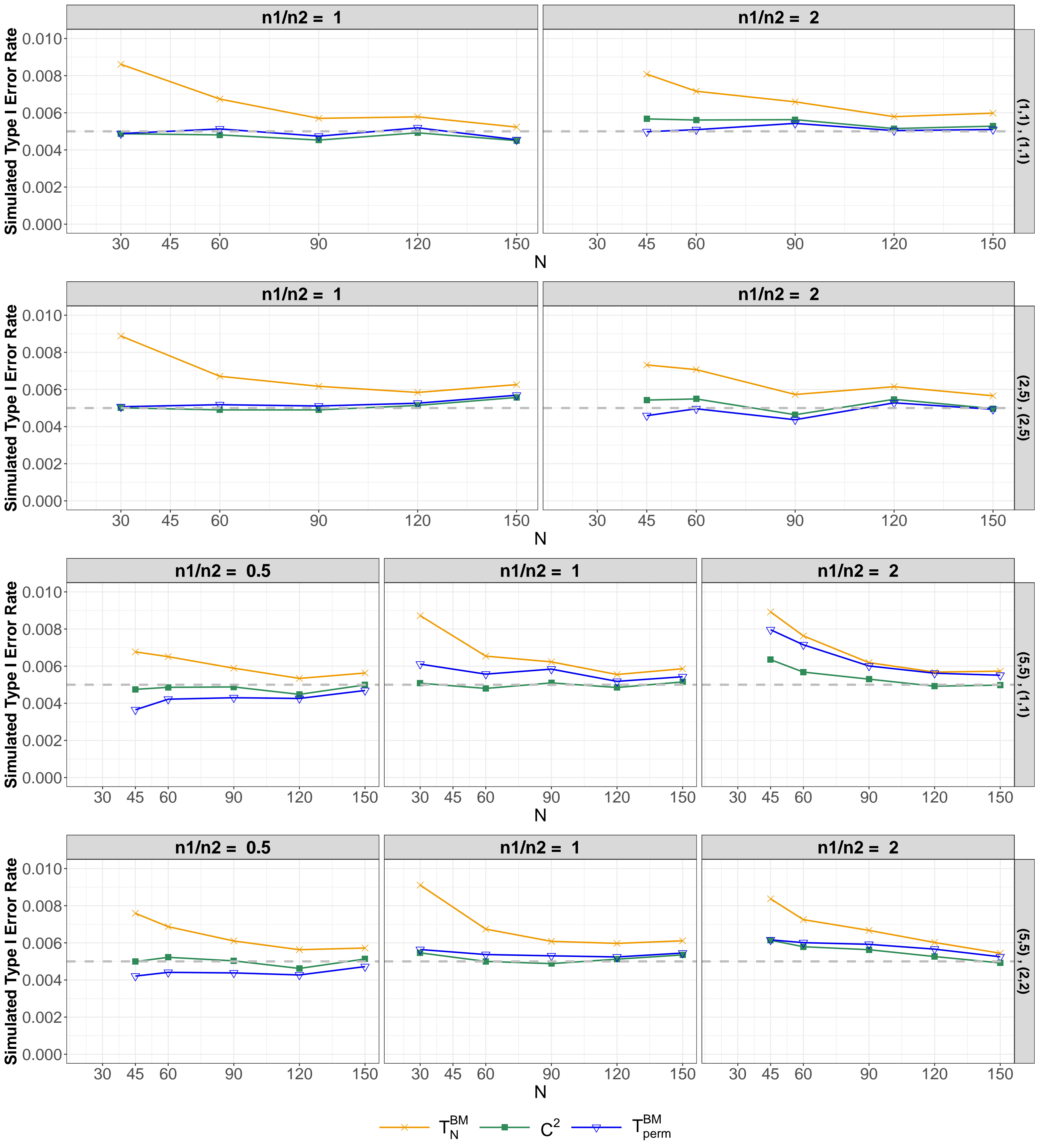

Supplement: Supplementary file 1 — Supporting information [file BIMJ-67-e70096-s002.zip › Schüürhuis_et_al_code_R2/R Code Submission/plots/Supplement/section2.2.3_beta_t1e.pdf]

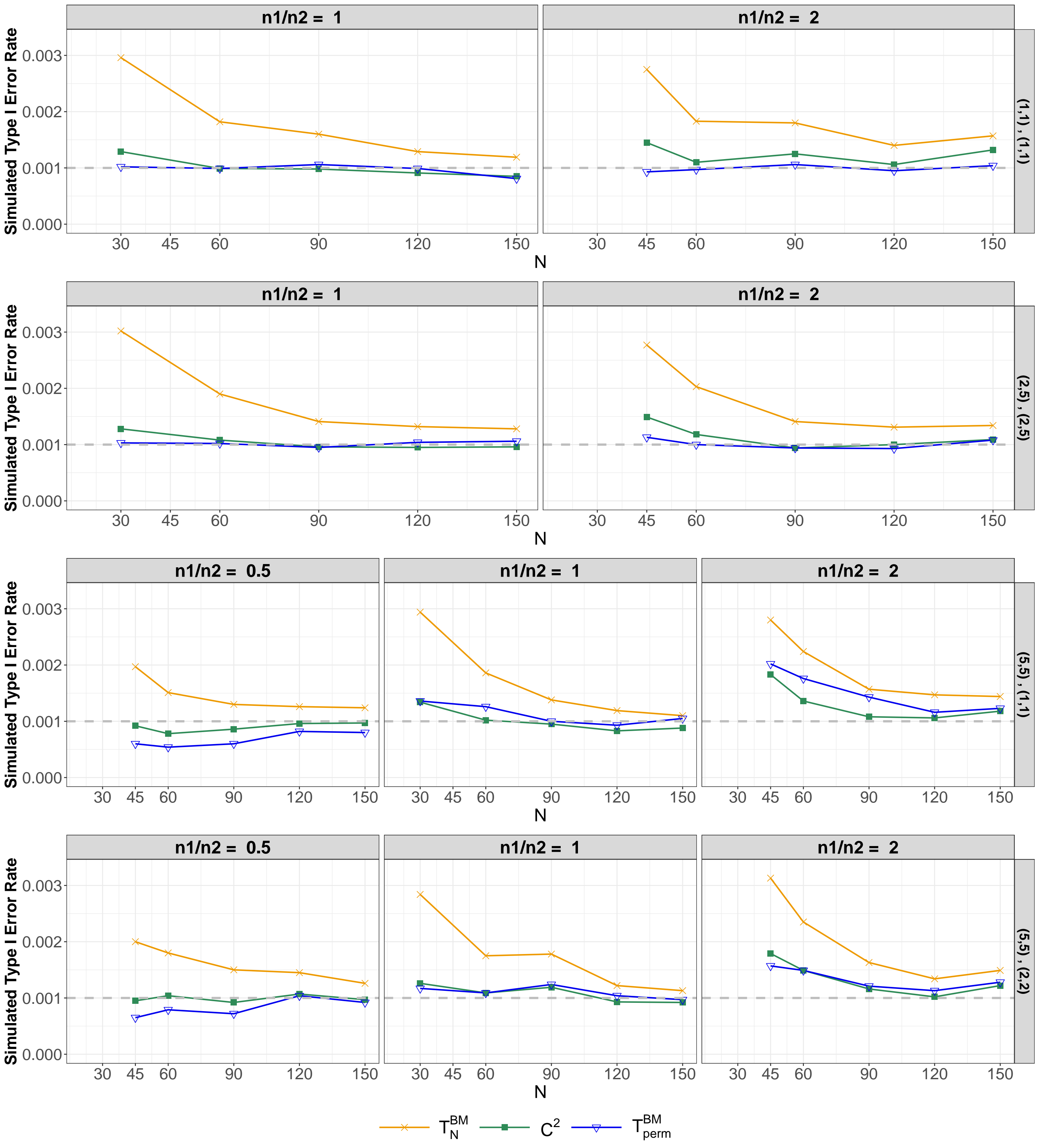

Supplement: Supplementary file 1 — Supporting information [file BIMJ-67-e70096-s002.zip › Schüürhuis_et_al_code_R2/R Code Submission/plots/Supplement/section2.2.4_beta_t1e.pdf]

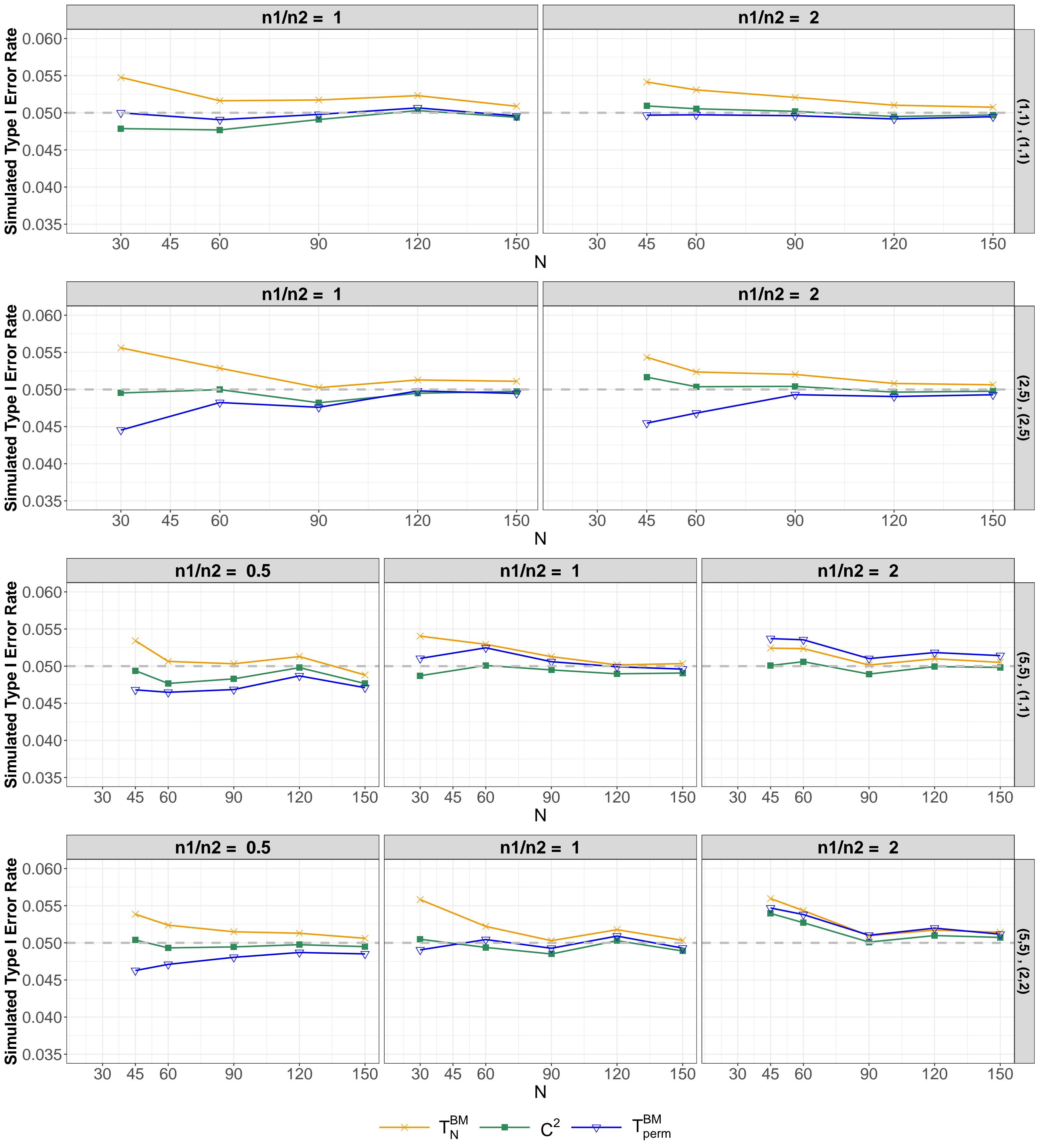

Supplement: Supplementary file 1 — Supporting information [file BIMJ-67-e70096-s002.zip › Schüürhuis_et_al_code_R2/R Code Submission/plots/Supplement/section2.3.1_ordinal_t1e.pdf]

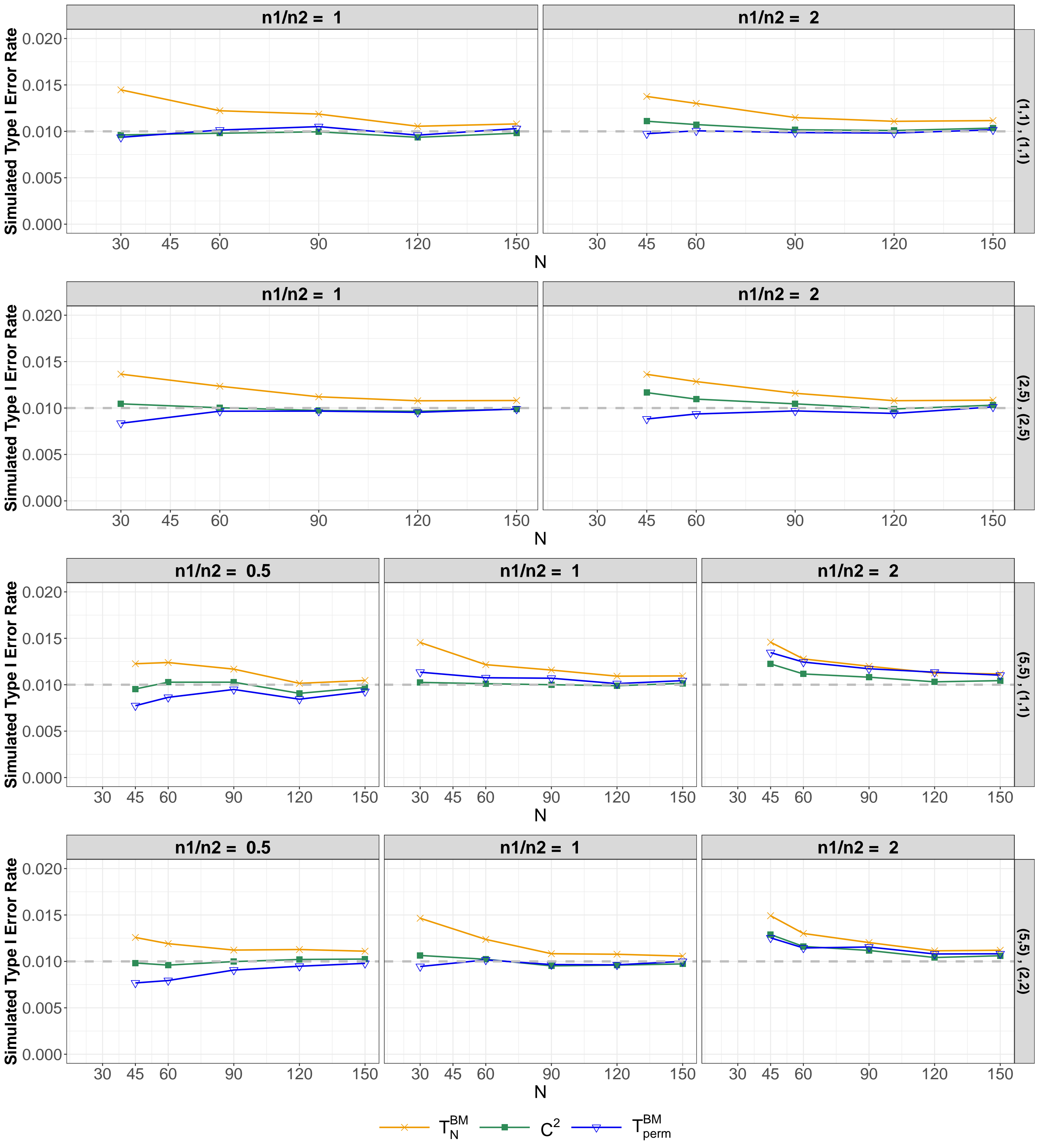

Supplement: Supplementary file 1 — Supporting information [file BIMJ-67-e70096-s002.zip › Schüürhuis_et_al_code_R2/R Code Submission/plots/Supplement/section2.3.2_ordinal_t1e.pdf]

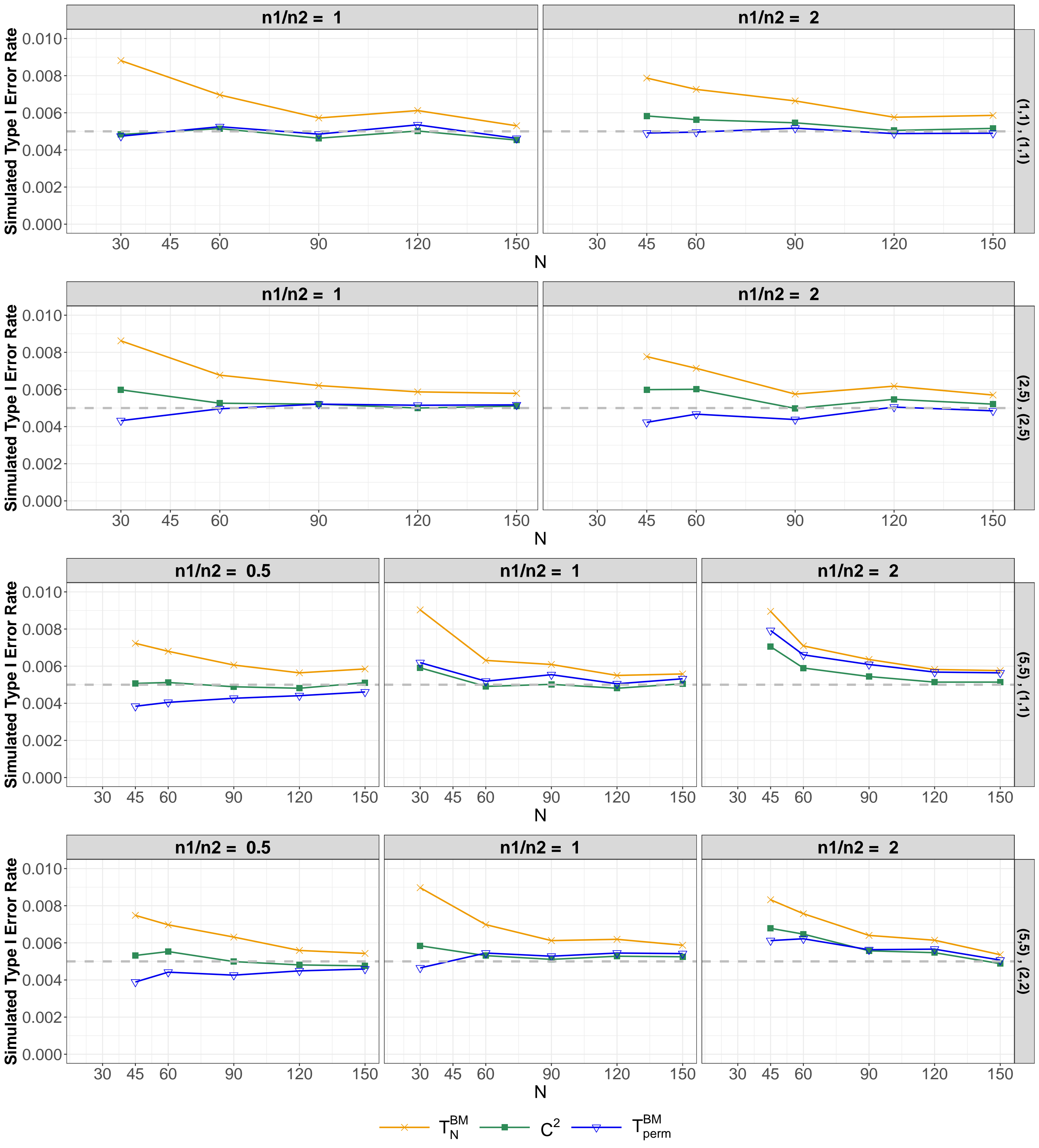

Supplement: Supplementary file 1 — Supporting information [file BIMJ-67-e70096-s002.zip › Schüürhuis_et_al_code_R2/R Code Submission/plots/Supplement/section2.3.3_ordinal_t1e.pdf]

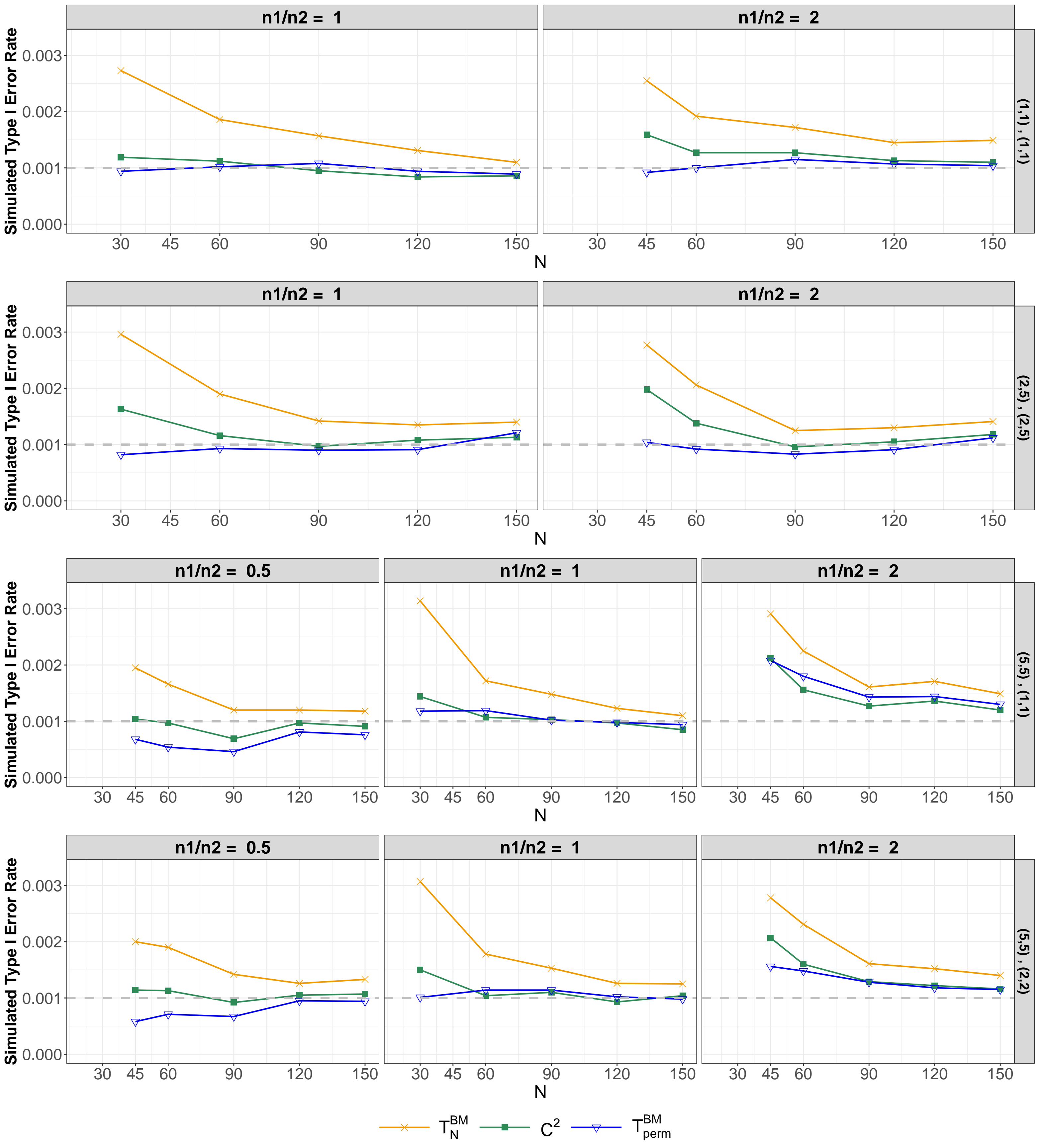

Supplement: Supplementary file 1 — Supporting information [file BIMJ-67-e70096-s002.zip › Schüürhuis_et_al_code_R2/R Code Submission/plots/Supplement/section2.3.4_ordinal_t1e.pdf]

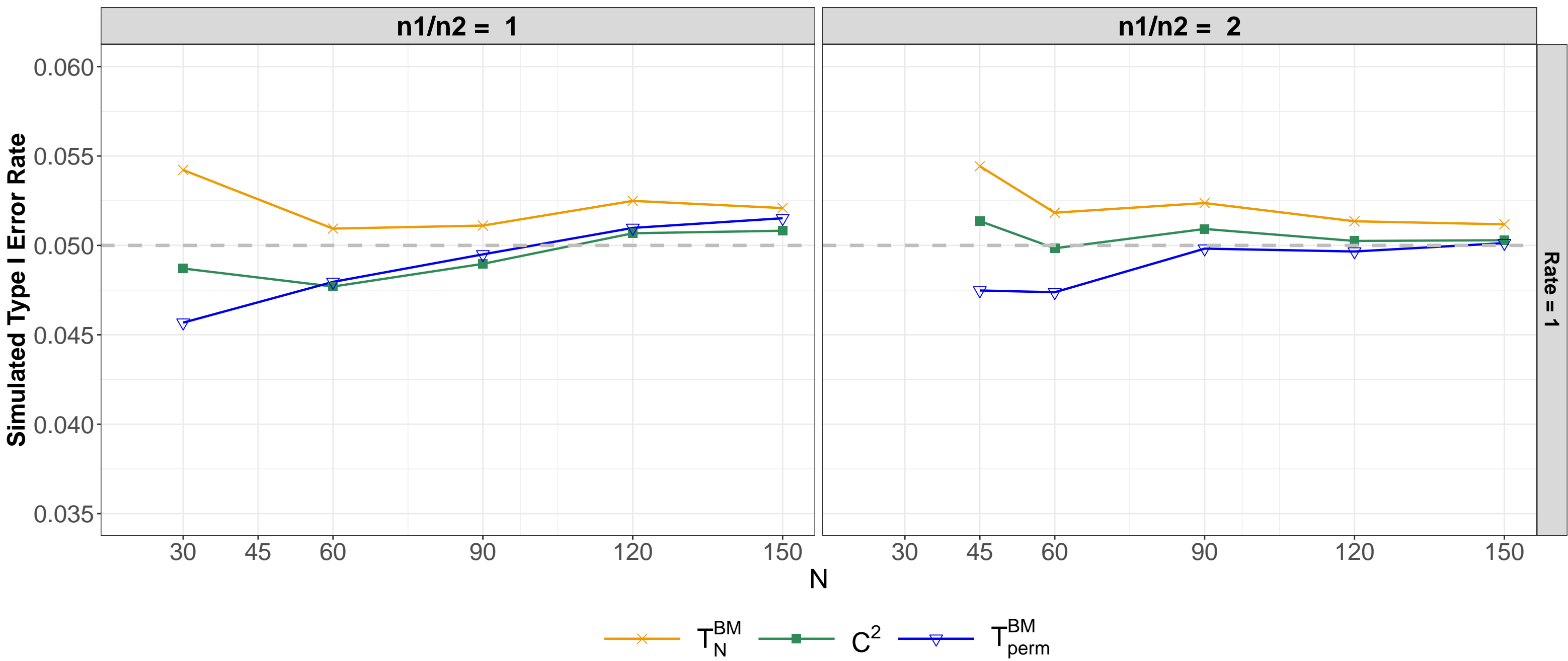

Supplement: Supplementary file 1 — Supporting information [file BIMJ-67-e70096-s002.zip › Schüürhuis_et_al_code_R2/R Code Submission/plots/Supplement/section2.4.1_poisson_t1e.pdf]

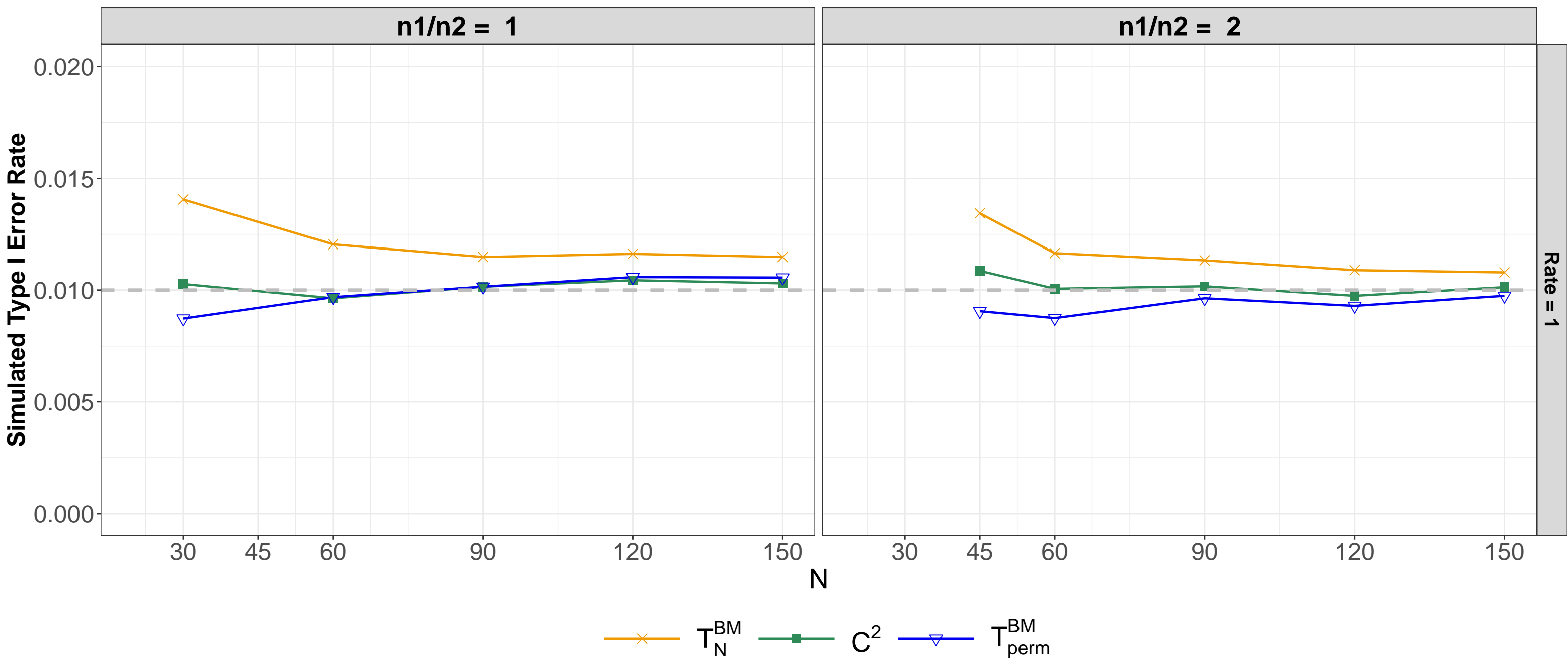

Supplement: Supplementary file 1 — Supporting information [file BIMJ-67-e70096-s002.zip › Schüürhuis_et_al_code_R2/R Code Submission/plots/Supplement/section2.4.2_poisson_t1e.pdf]

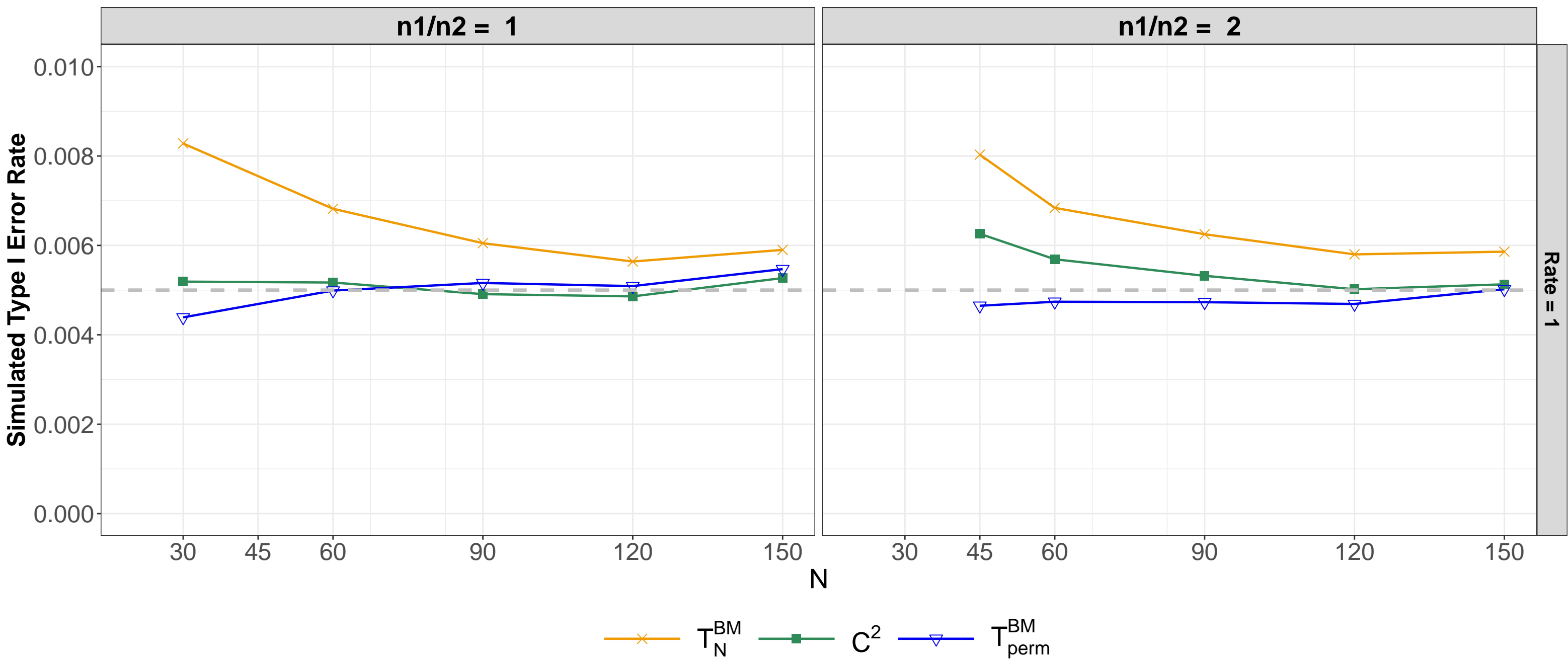

Supplement: Supplementary file 1 — Supporting information [file BIMJ-67-e70096-s002.zip › Schüürhuis_et_al_code_R2/R Code Submission/plots/Supplement/section2.4.3_poisson_t1e.pdf]

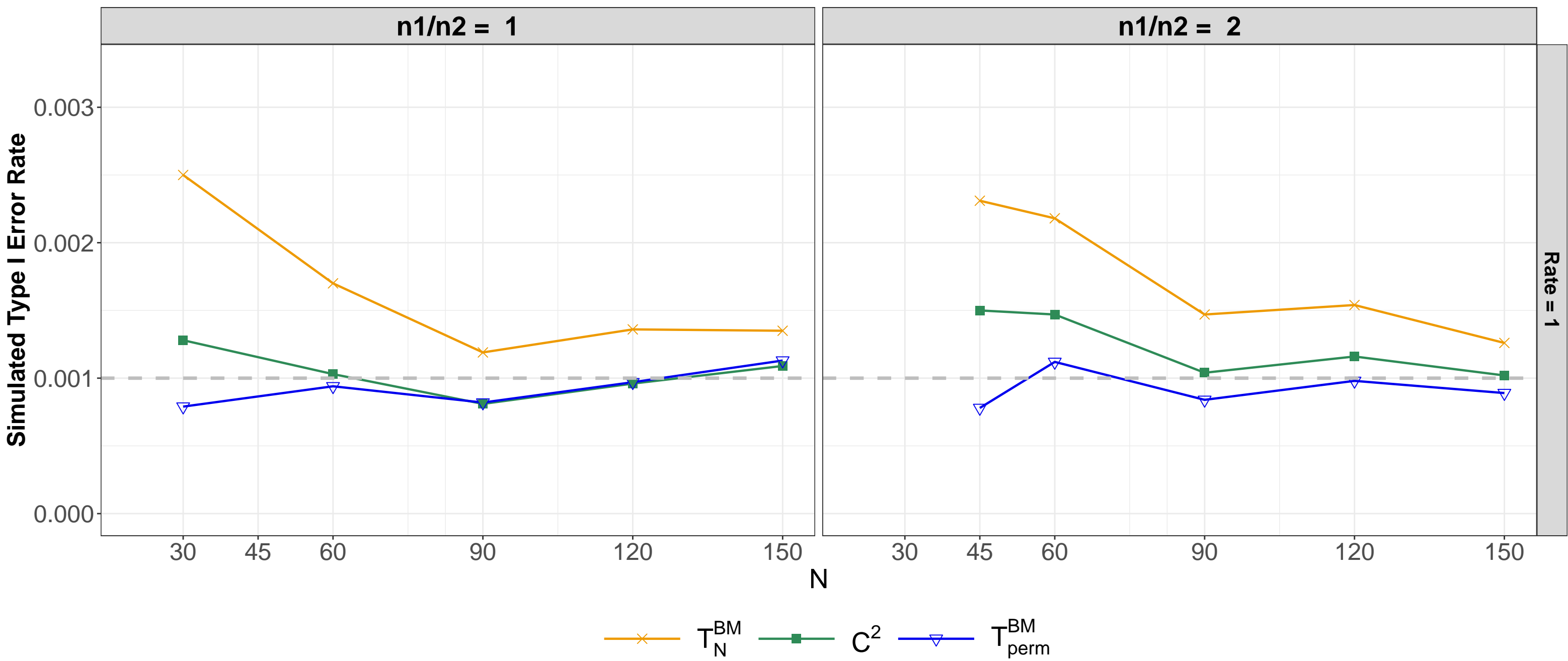

Supplement: Supplementary file 1 — Supporting information [file BIMJ-67-e70096-s002.zip › Schüürhuis_et_al_code_R2/R Code Submission/plots/Supplement/section2.4.4_poisson_t1e.pdf]

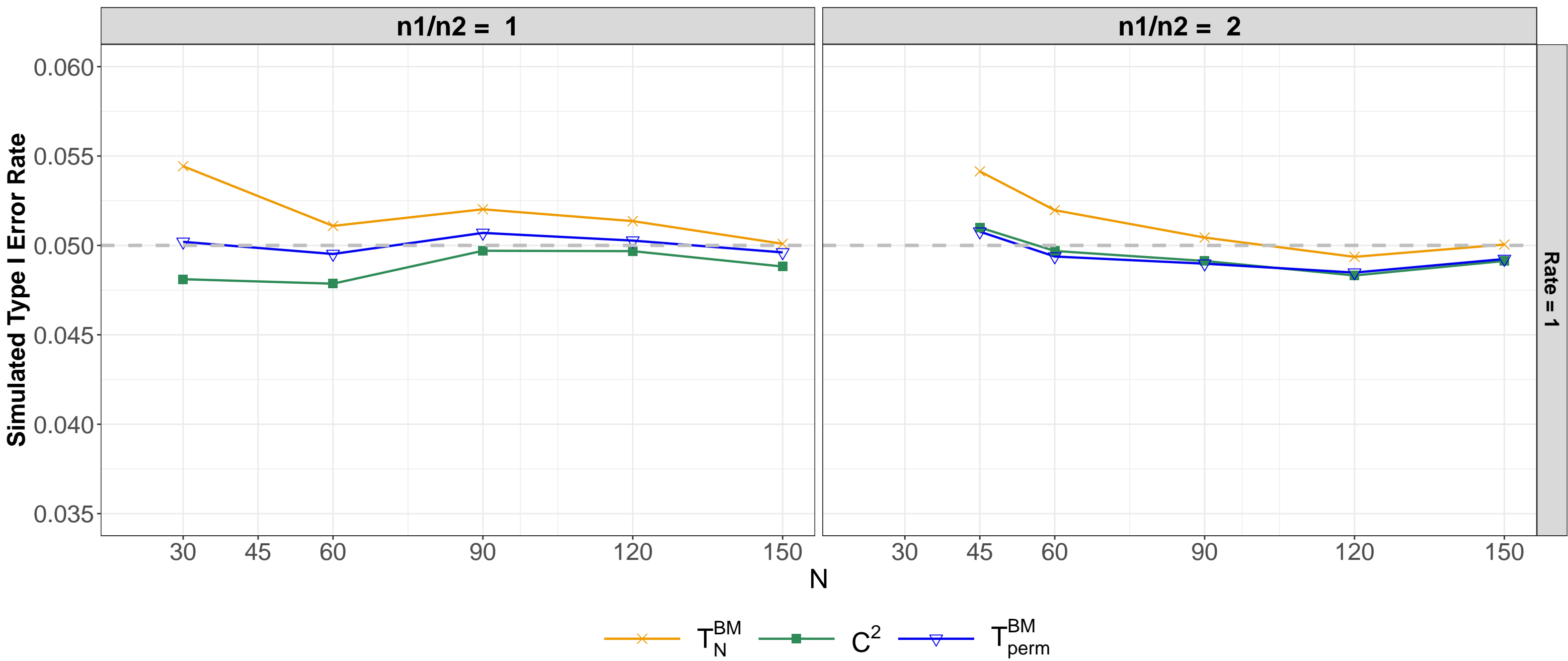

Supplement: Supplementary file 1 — Supporting information [file BIMJ-67-e70096-s002.zip › Schüürhuis_et_al_code_R2/R Code Submission/plots/Supplement/section2.5.1_exponential_t1e.pdf]

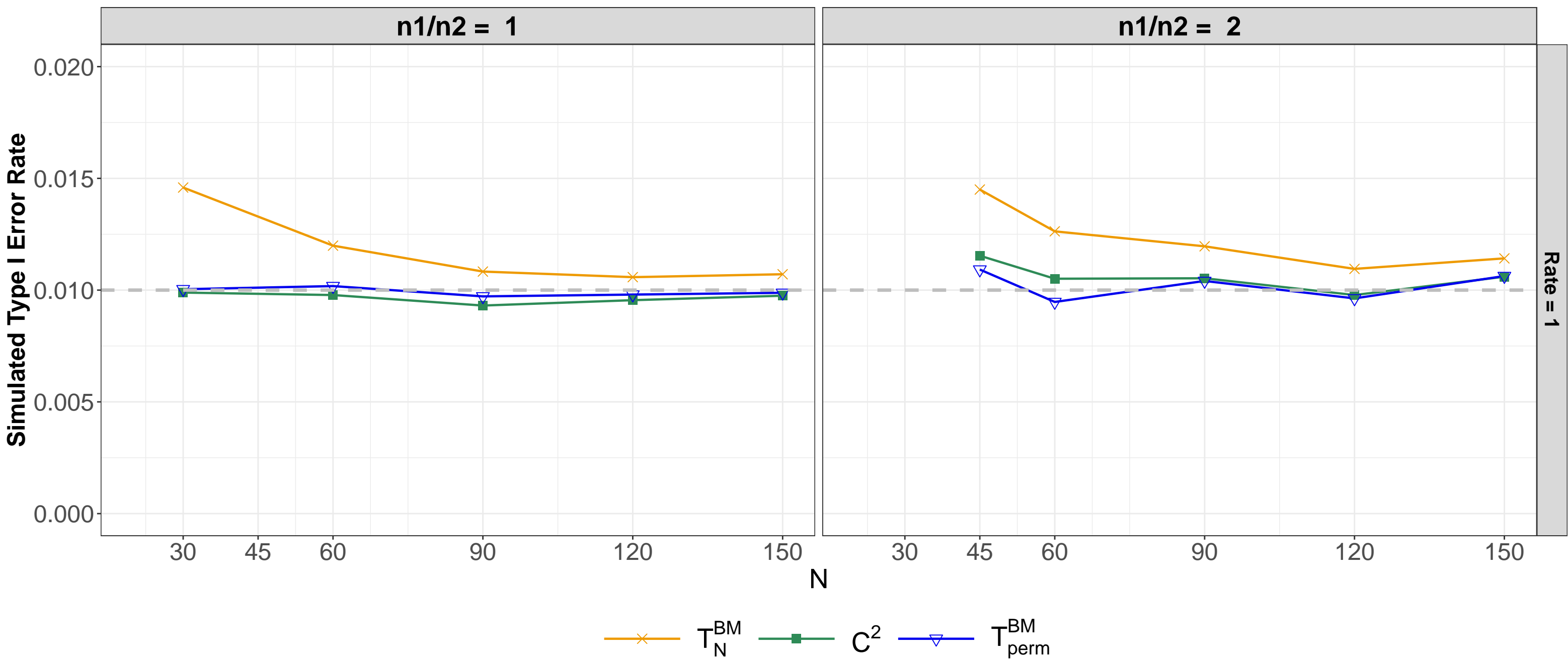

Supplement: Supplementary file 1 — Supporting information [file BIMJ-67-e70096-s002.zip › Schüürhuis_et_al_code_R2/R Code Submission/plots/Supplement/section2.5.2_exponential_t1e.pdf]

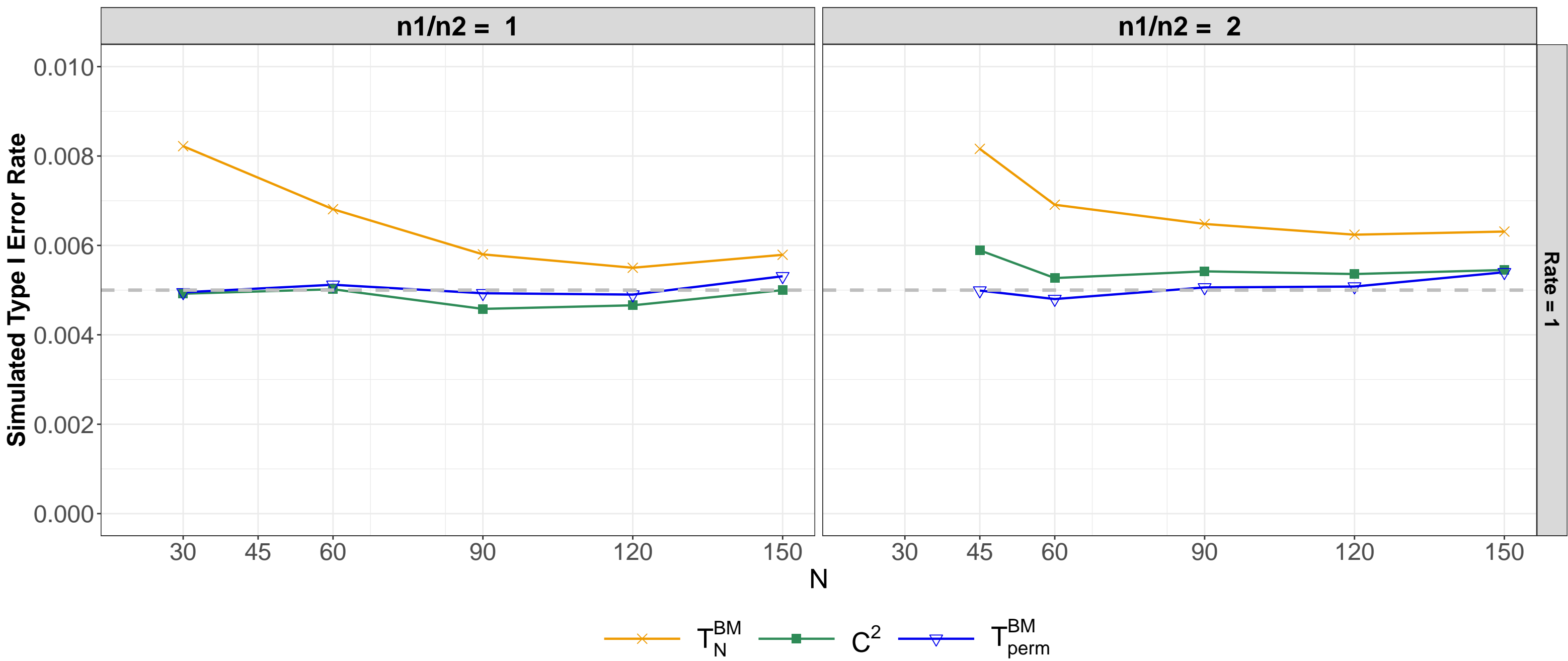

Supplement: Supplementary file 1 — Supporting information [file BIMJ-67-e70096-s002.zip › Schüürhuis_et_al_code_R2/R Code Submission/plots/Supplement/section2.5.3_exponential_t1e.pdf]

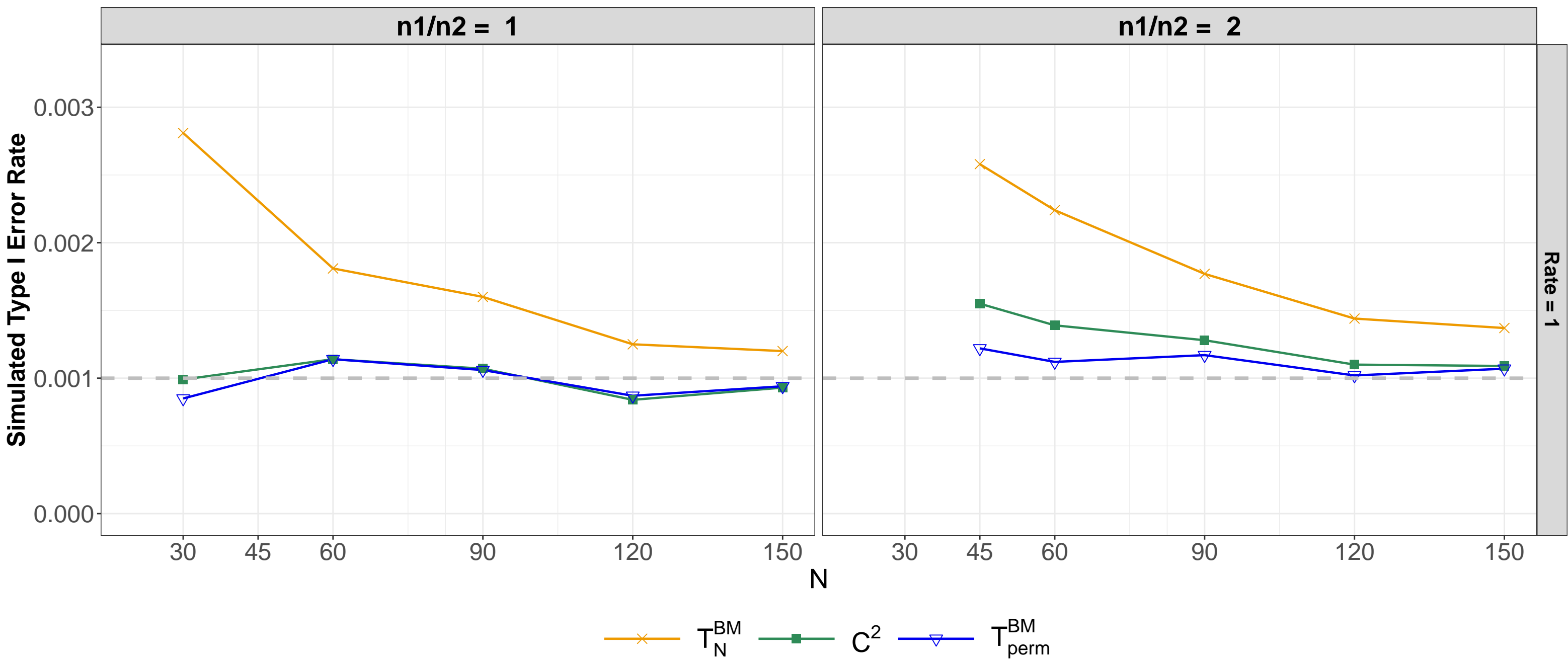

Supplement: Supplementary file 1 — Supporting information [file BIMJ-67-e70096-s002.zip › Schüürhuis_et_al_code_R2/R Code Submission/plots/Supplement/section2.5.4_exponential_t1e.pdf]

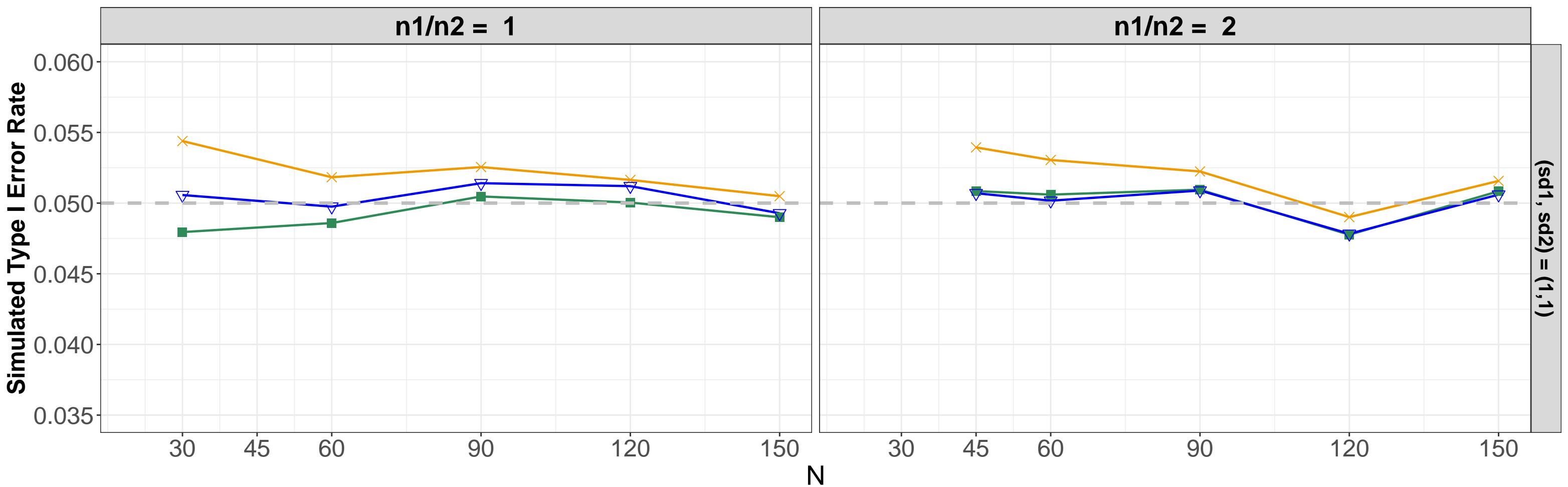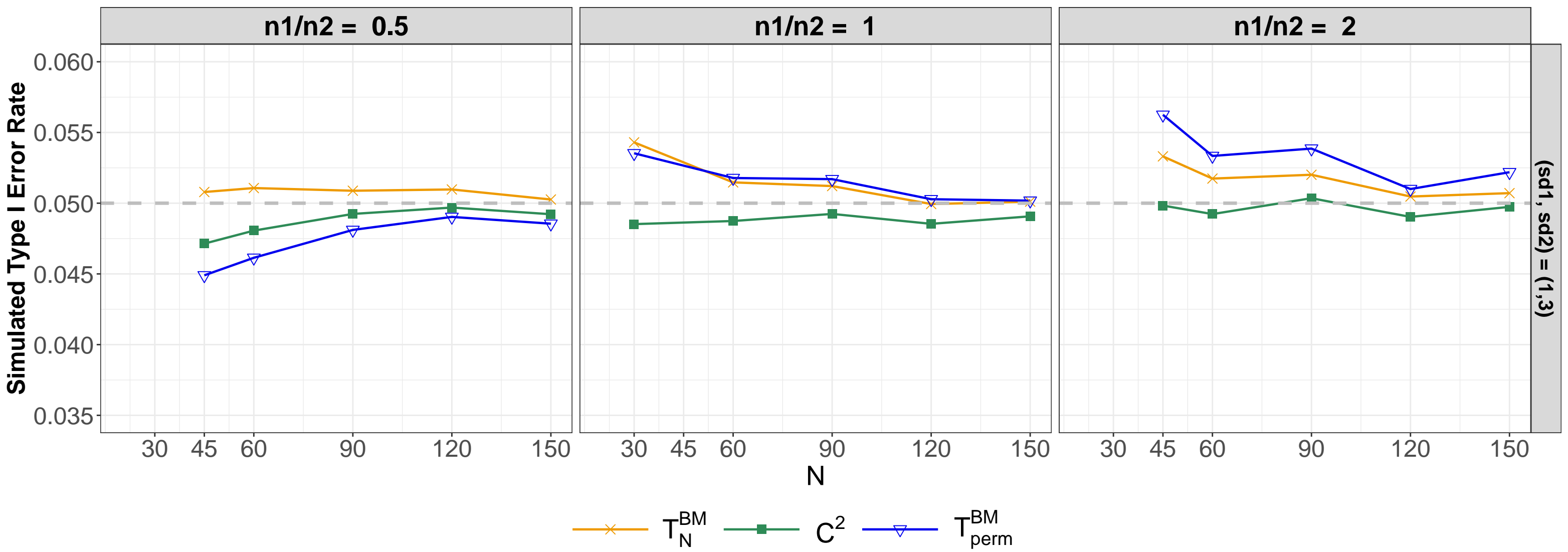

Supplement: Supplementary file 1 — Supporting information [file BIMJ-67-e70096-s002.zip › Schüürhuis_et_al_code_R2/R Code Submission/plots/Supplement/section2.6.1_laplace_t1e.pdf]

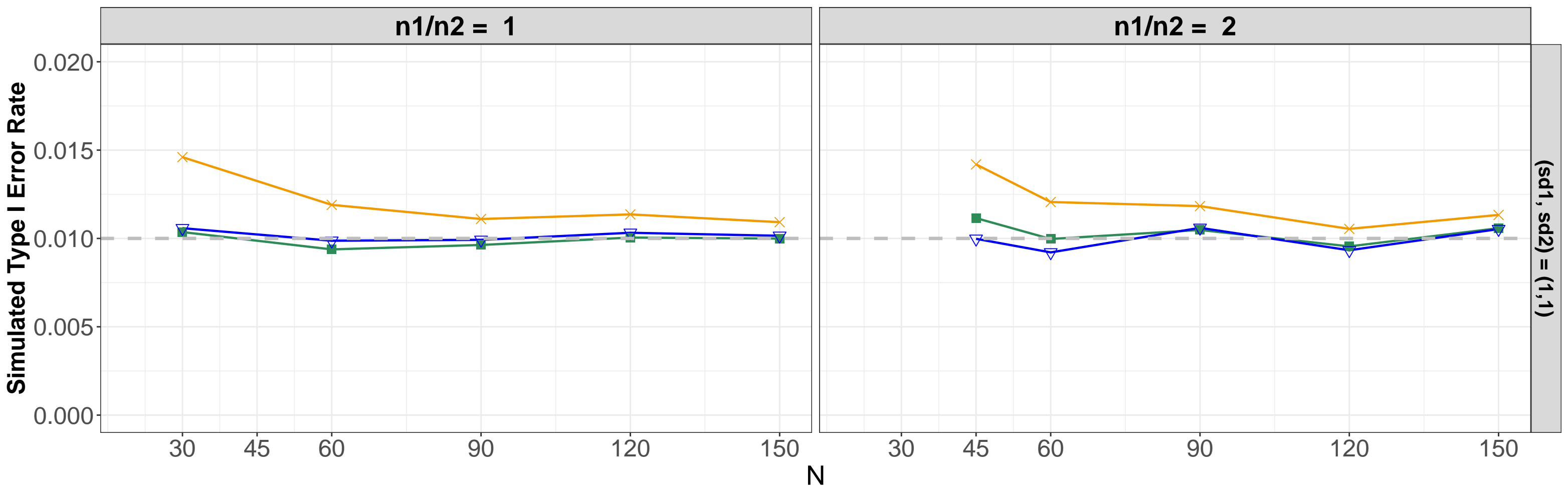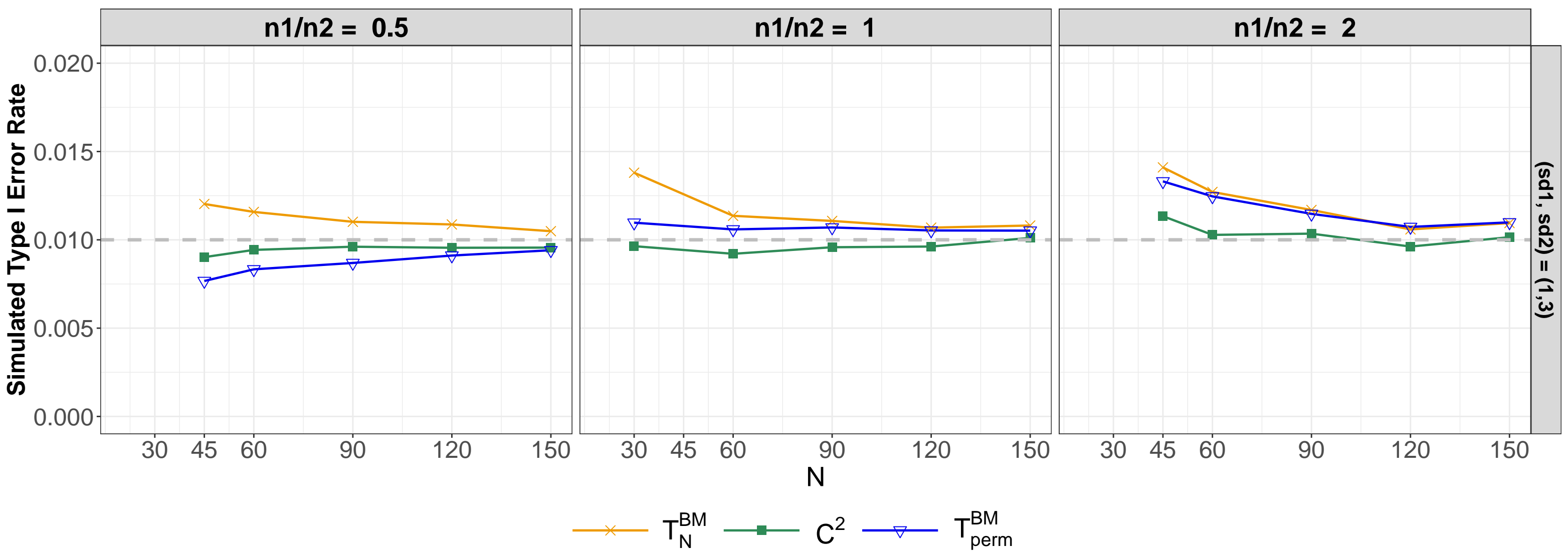

Supplement: Supplementary file 1 — Supporting information [file BIMJ-67-e70096-s002.zip › Schüürhuis_et_al_code_R2/R Code Submission/plots/Supplement/section2.6.2_laplace_t1e.pdf]

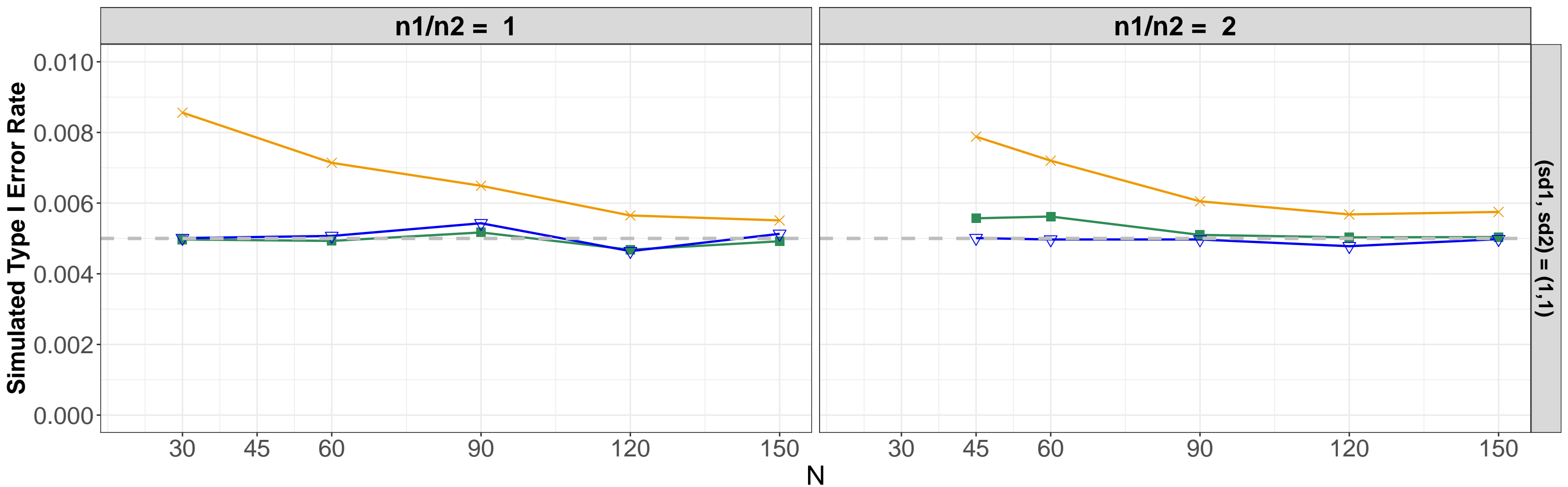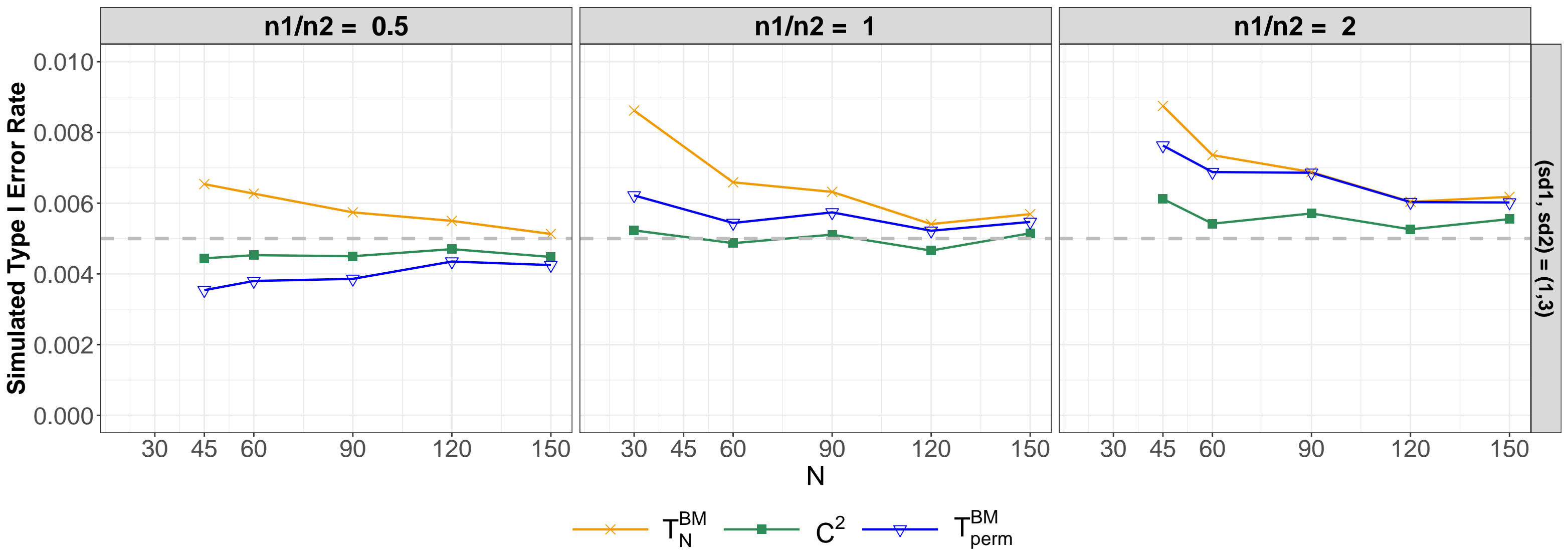

Supplement: Supplementary file 1 — Supporting information [file BIMJ-67-e70096-s002.zip › Schüürhuis_et_al_code_R2/R Code Submission/plots/Supplement/section2.6.3_laplace_t1e.pdf]

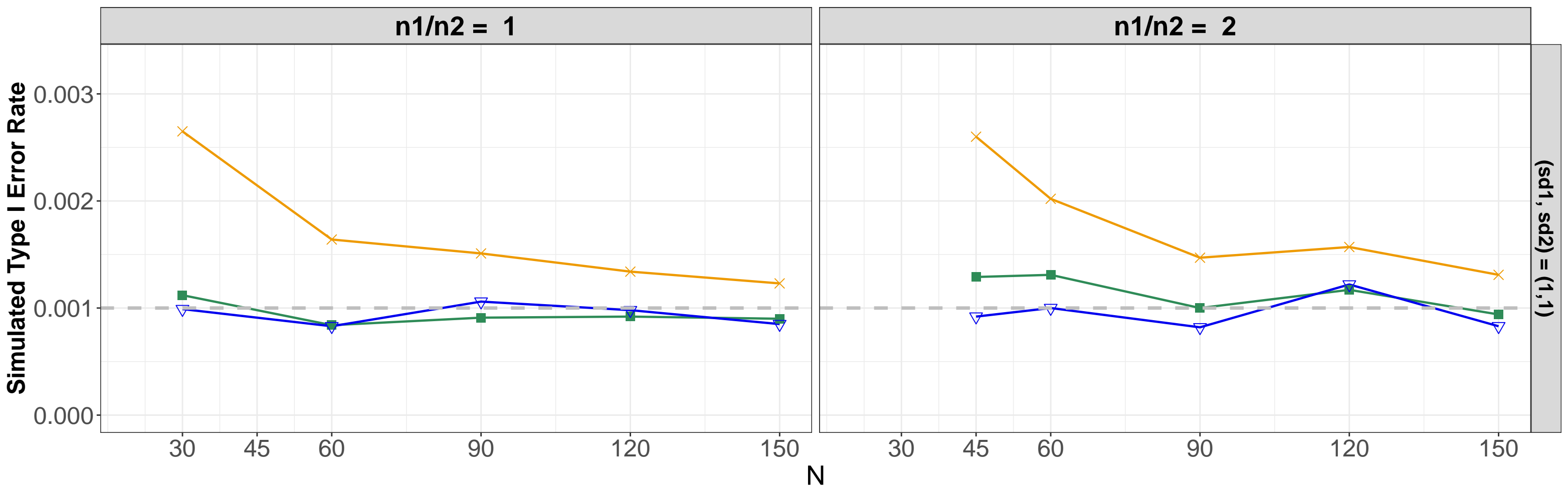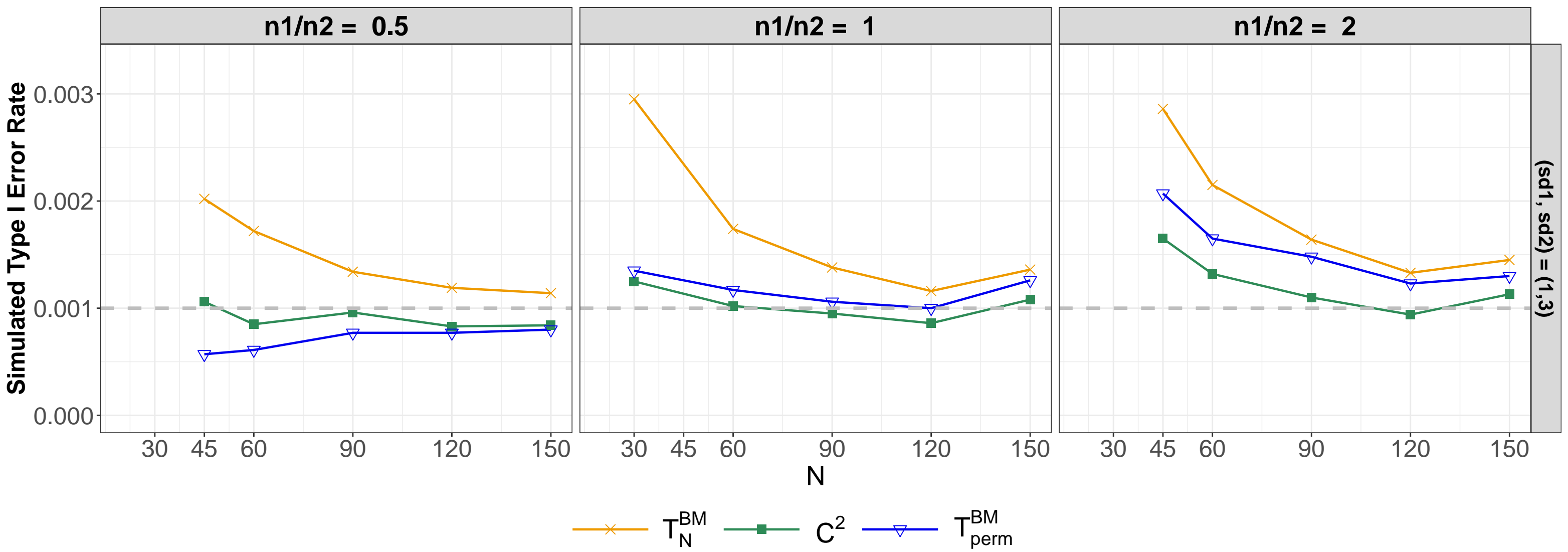

Supplement: Supplementary file 1 — Supporting information [file BIMJ-67-e70096-s002.zip › Schüürhuis_et_al_code_R2/R Code Submission/plots/Supplement/section2.6.4_laplace_t1e.pdf]

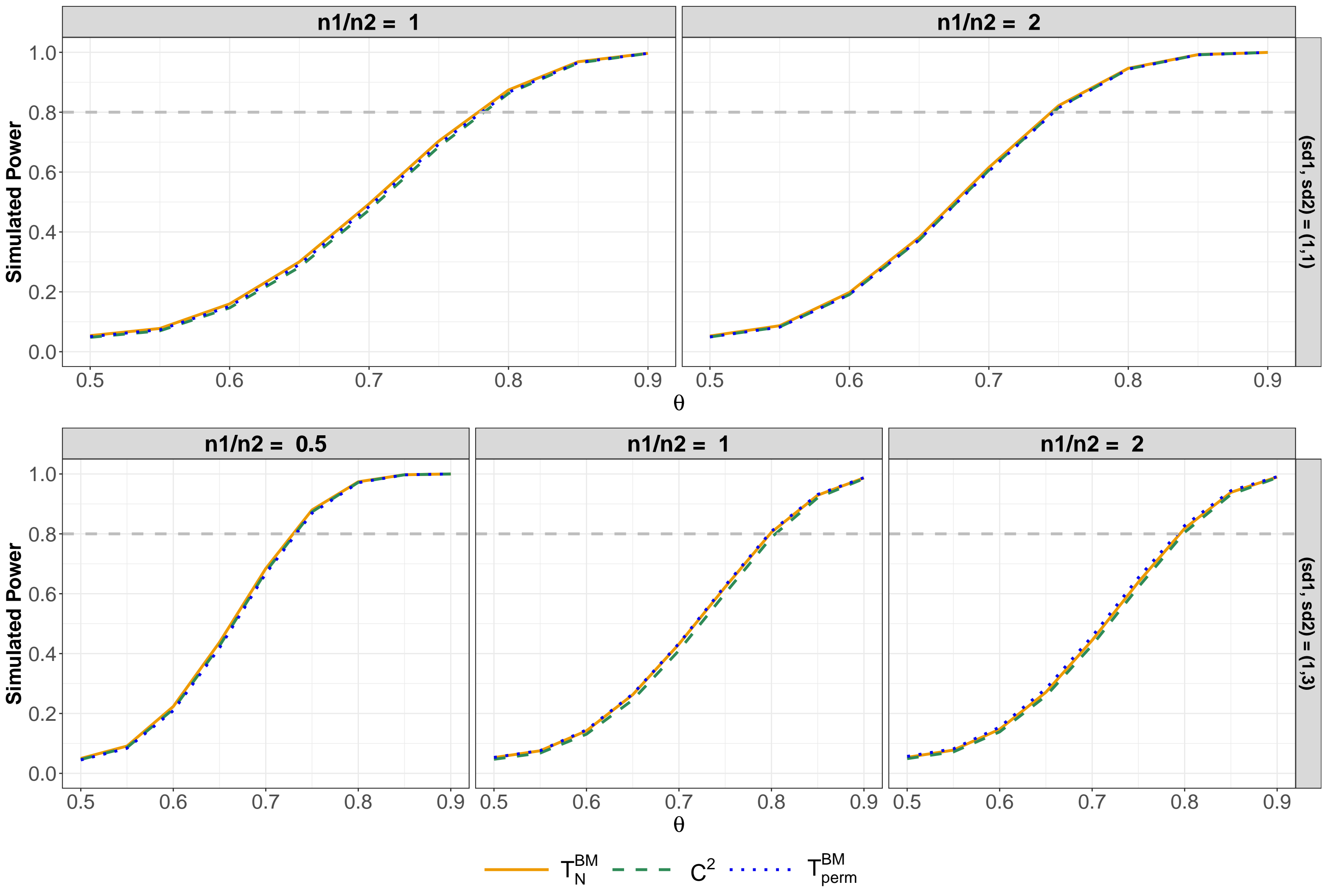

Supplement: Supplementary file 1 — Supporting information [file BIMJ-67-e70096-s002.zip › Schüürhuis_et_al_code_R2/R Code Submission/plots/Supplement/section3.1_normal_pwr.pdf]

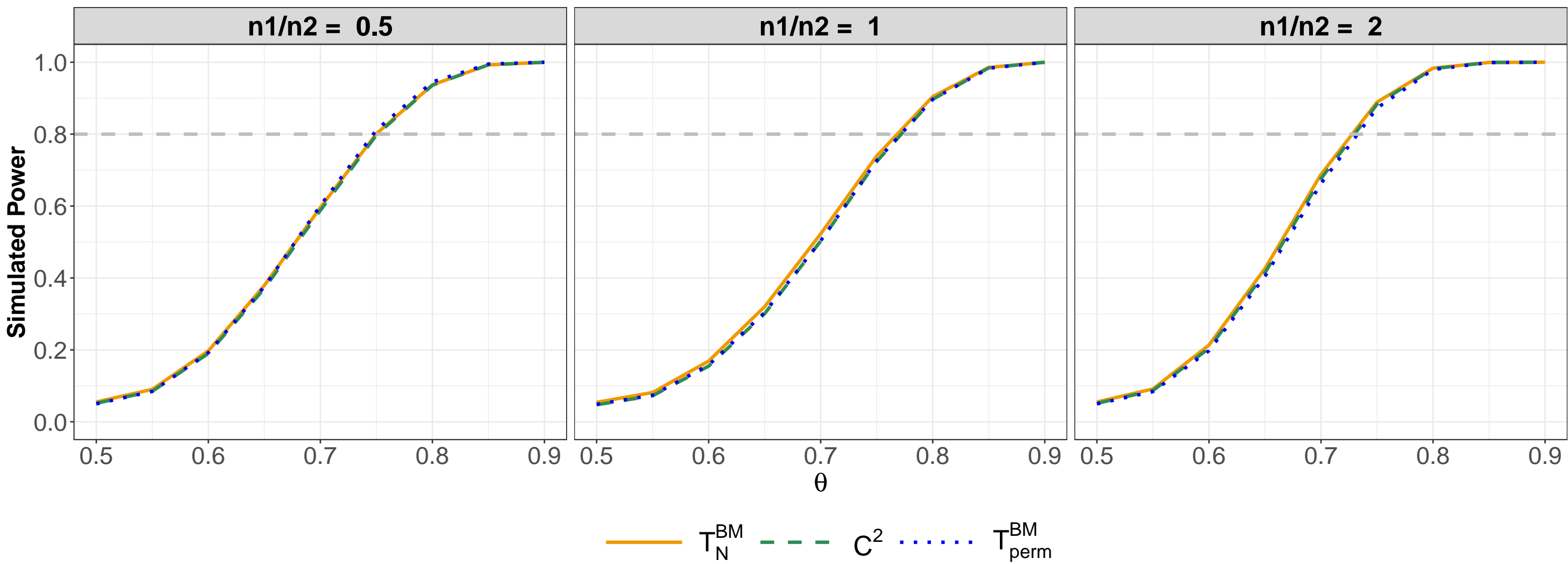

Supplement: Supplementary file 1 — Supporting information [file BIMJ-67-e70096-s002.zip › Schüürhuis_et_al_code_R2/R Code Submission/plots/Supplement/section3.2_ordinal_pwr.pdf]

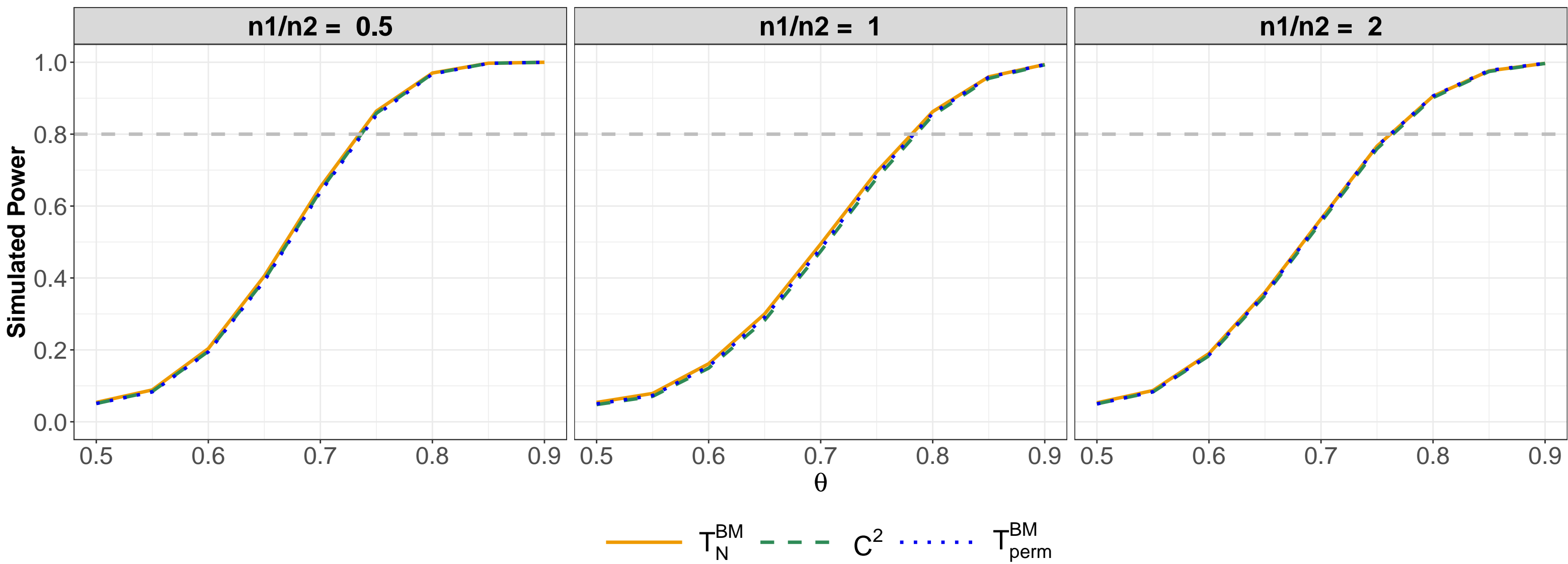

Supplement: Supplementary file 1 — Supporting information [file BIMJ-67-e70096-s002.zip › Schüürhuis_et_al_code_R2/R Code Submission/plots/Supplement/section3.3_exponential_pwr.pdf]
