# Supplementary material for: A New Approach to the Nonparametric Behrens–Fisher Problem With Compatible Confidence Intervals
Source: Biom J. 2025 Nov 9;67(6):e70096. doi: 10.1002/bimj.70096 (PMC12598137; doi:10.1002/bimj.70096)
Supplement: Supplementary file 2 — Supporting Information [file BIMJ-67-e70096-s001.pdf]

Biometrical Journal **XX** (YYYY) ZZ, zzz–zzz / DOI: 10.1002/bimj.20.00100000

Supplementary Material for the Article: A New Approach to the Nonparametric Behrens-Fisher Problem with Compatible Confidence Intervals

Stephen Schüürhuis[*,1], **Frank Konietschke** [1], and **Edgar Brunner** [2]

[1] Charité - Universitätsmedizin Berlin, Corporate member of Freie Universität Berlin and Humboldt-Universität zu Berlin, Institute of Biometry and Clinical Epidemiology, Berlin, Germany

[2] University of Göttingen, Department of Medical Statistics, Göttingen, Germany

*Corresponding author: e-mail: stephen.schueuerhuis@charite.de

© YYYY WILEY-VCH Verlag GmbH & Co. KGaA, Weinheim

Table of Contents

2

1 **Some Distributions considered in the Simulation study** 4

1.1 Type-I Error Rate in Table 3 ($\mathcal{H}_0$) 4

1.2 Power/Coverage in Table 4 ($\mathcal{H}_1$) 5

2 **Type-I Error Rate Results** 6

2.1 Normal Distribution 6

2.1.1 $\alpha = 0.05$ 6

2.1.2 $\alpha = 0.01$ 7

2.1.3 $\alpha = 0.005$ 8

2.1.4 $\alpha = 0.001$ 9

2.2 Beta Distribution 10

2.2.1 $\alpha = 0.05$ 10

2.2.2 $\alpha = 0.01$ 11

2.2.3 $\alpha = 0.005$ 12

2.2.4 $\alpha = 0.001$ 13

2.3 5-Point Ordered Categorical Data 14

2.3.1 $\alpha = 0.05$ 14

2.3.2 $\alpha = 0.01$ 15

2.3.3 $\alpha = 0.005$ 16

2.3.4 $\alpha = 0.001$ 17

2.4 Poisson Distribution 18

2.4.1 $\alpha = 0.05$ 18

2.4.2 $\alpha = 0.01$ 18

2.4.3 $\alpha = 0.005$ 19

2.4.4 $\alpha = 0.001$ 19

2.5 Exponential Distribution 20

2.5.1 $\alpha = 0.05$ 20

2.5.2 $\alpha = 0.01$ 20

2.5.3 $\alpha = 0.005$ 21

2.5.4 $\alpha = 0.001$ 21

2.6 Laplace Distribution 22

2.6.1 $\alpha = 0.05$ 22

2.6.2 $\alpha = 0.01$ 23

2.6.3 $\alpha = 0.005$ 24

2.6.4 $\alpha = 0.001$ 25

3 **Power Results** 26

3.1 Normal Distribution 26

3.2 5-Point Ordered Categorical Data 26

3.3 Exponential Distribution 27

4 Coverage Probability Results 28

4.1 Normal Distribution 28

© YYYY WILEY-VCH Verlag GmbH & Co. KGaA, Weinheim

1 Some Distributions considered in the Simulation study

1.1 Type-I Error Rate in Table 3 ($\mathcal{H}_0$)

The figures below illustrate the heteroscedastic scenarios used in the type-I error rate simulation study, as detailed in Table 3 of the main manuscript. The Beta distributions were analyzed both in their continuous form and after discretization into ordered categorical data.

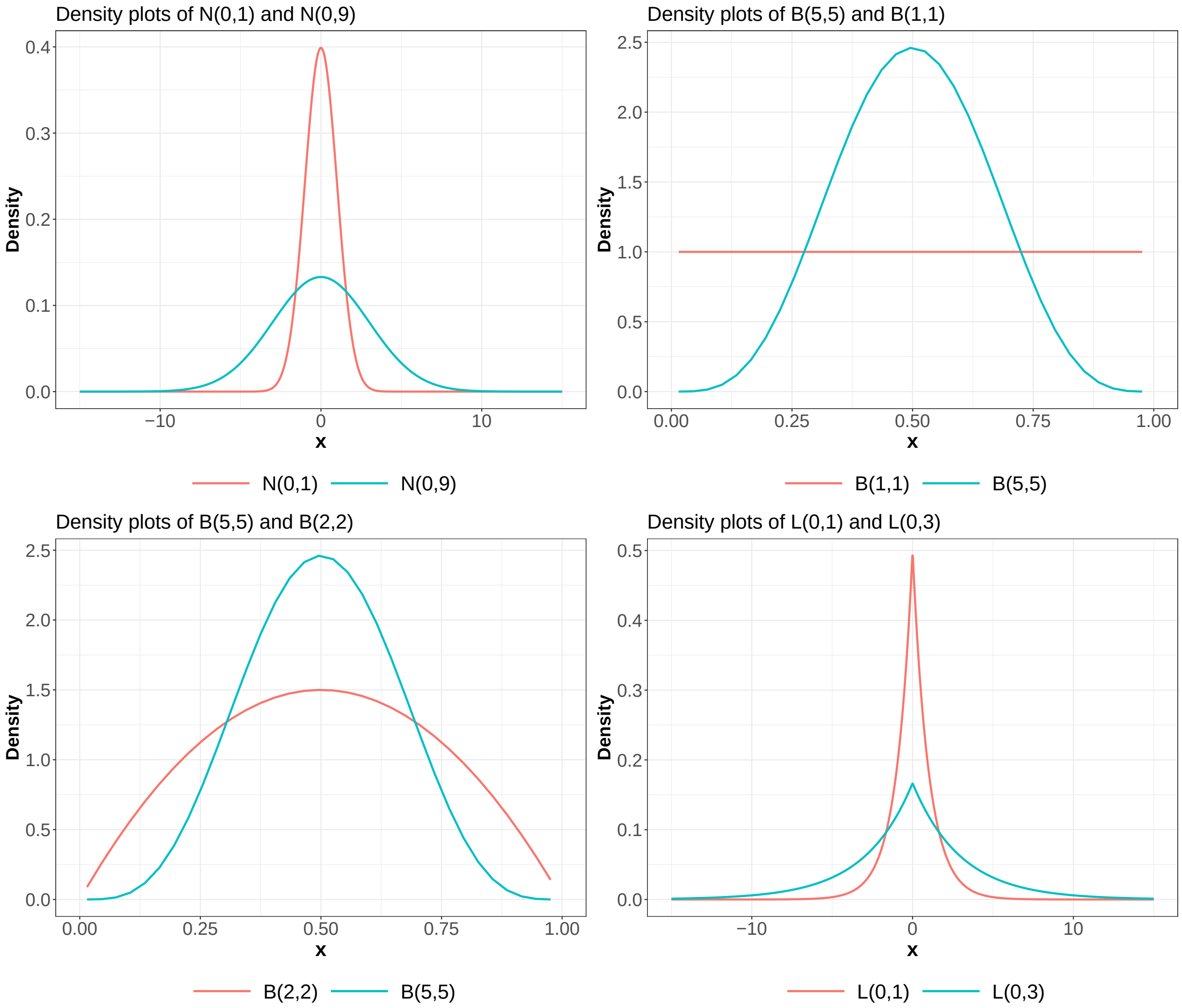

© YYYY WILEY-VCH Verlag GmbH & Co. KGaA, Weinheim

1.2 Power/Coverage in Table 4 ($\mathcal{H}_1$)

The figures below illustrate exemplary scenarios for selected cases where $\theta > 0.5$, along with the corresponding distributional parameters used in the power simulation study, as detailed in Table 4 of the main manuscript. Moreover, the normal distribution is also considered in the coverage simulation study in Section 5.3. The Beta distributions were analyzed after discretization into ordered categorical data; therefore, the value $\theta = 0.74$ refers to the effect on the discretized scale.

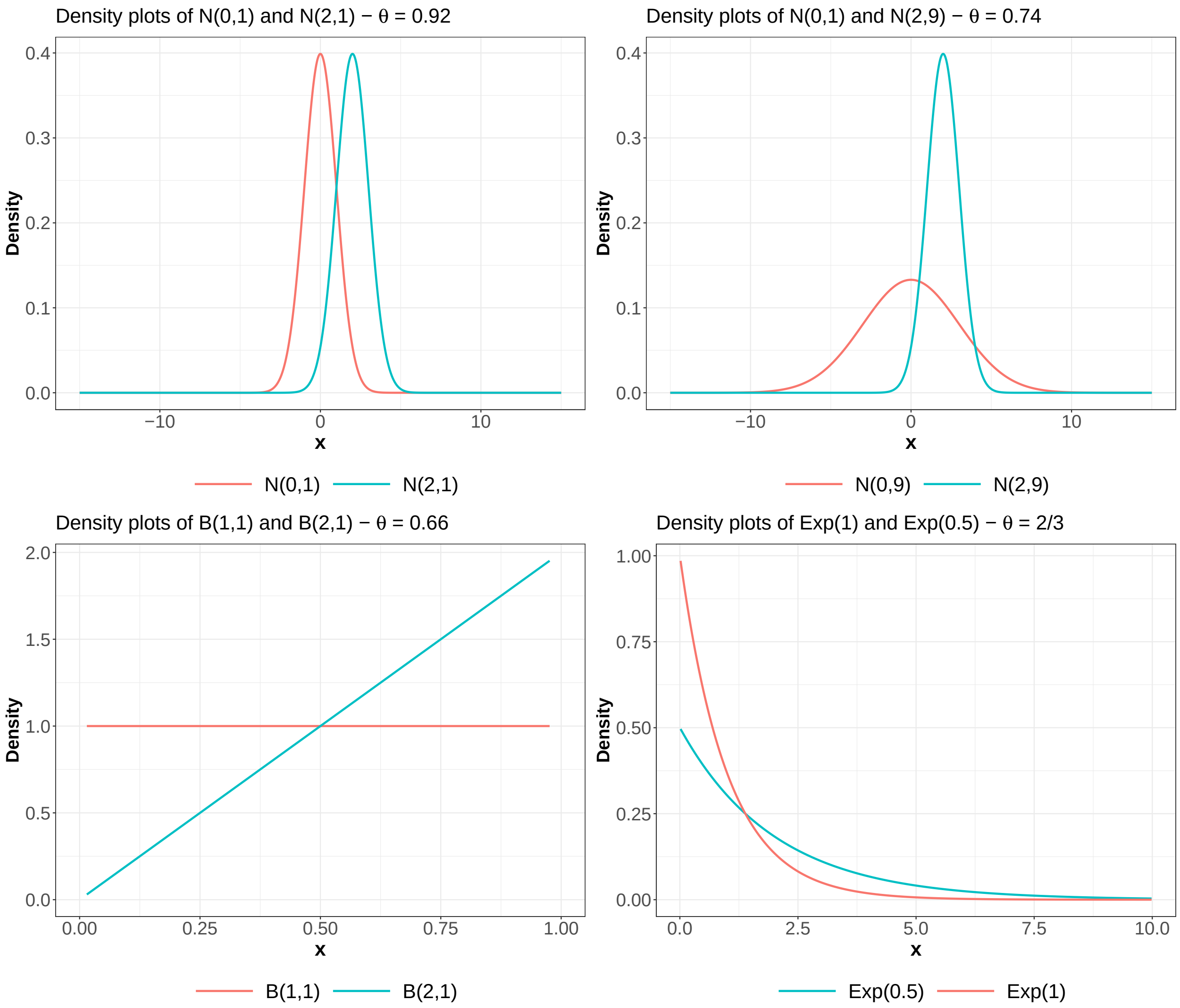

© YYYY WILEY-VCH Verlag GmbH & Co. KGaA, Weinheim

2 Type-I Error Rate Results

2.1 Normal Distribution

2.1.1 $\alpha = 0.05$

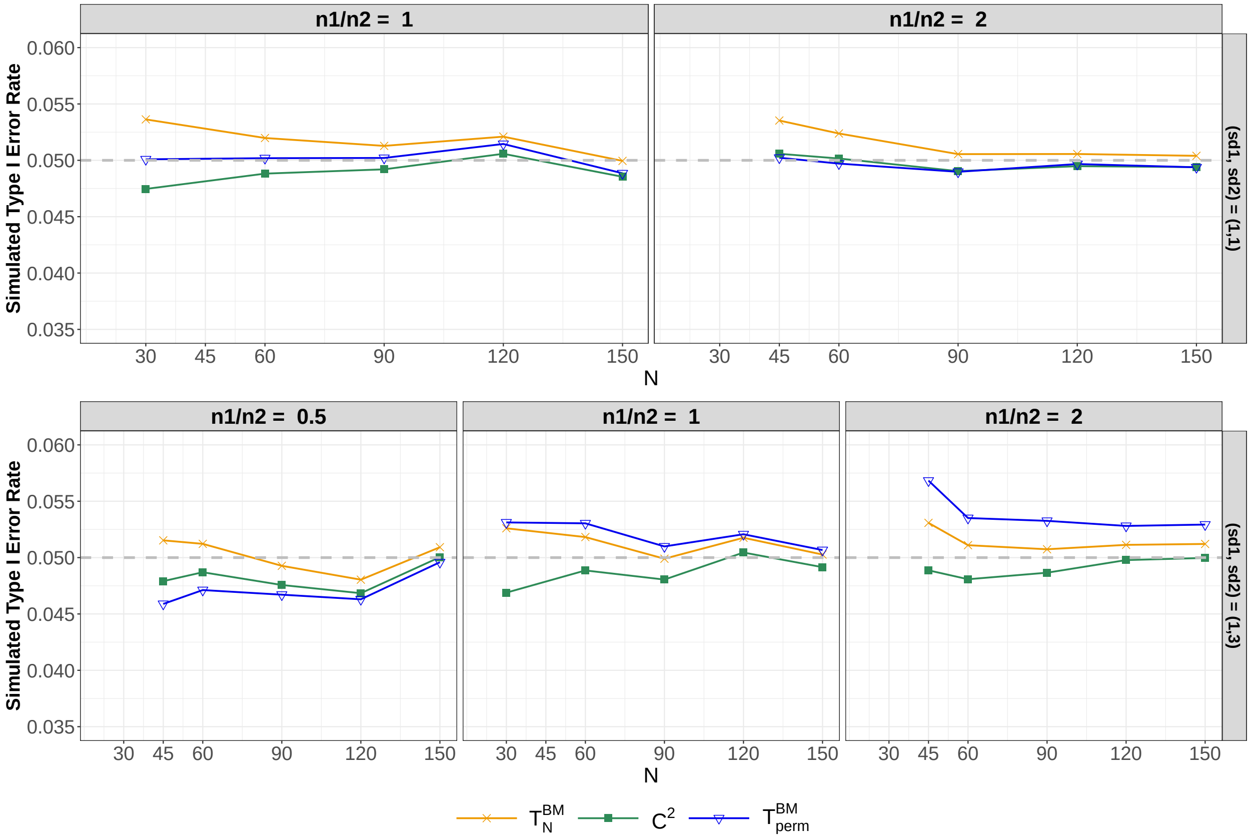

2.1.2 $\alpha = 0.01$

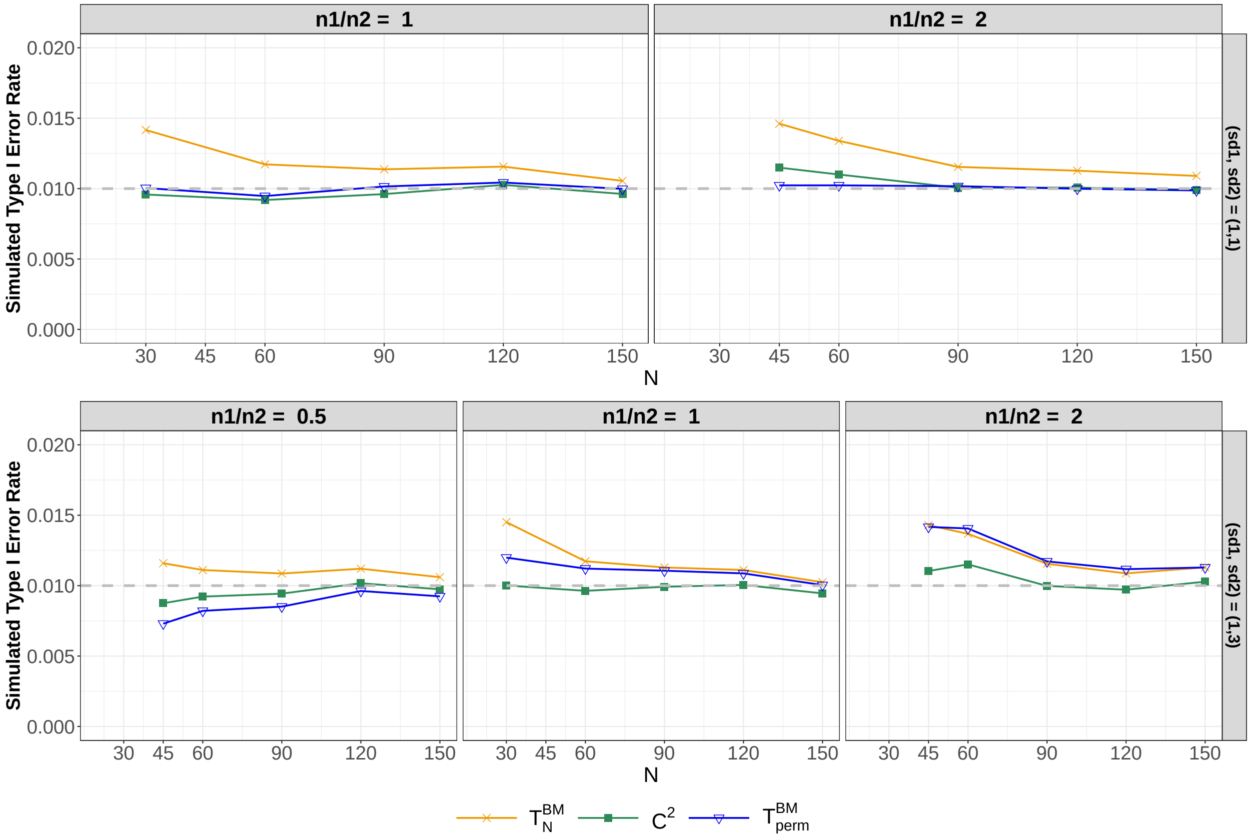

© YYYY WILEY-VCH Verlag GmbH & Co. KGaA, Weinheim

2.1.3 $\alpha = 0.005$

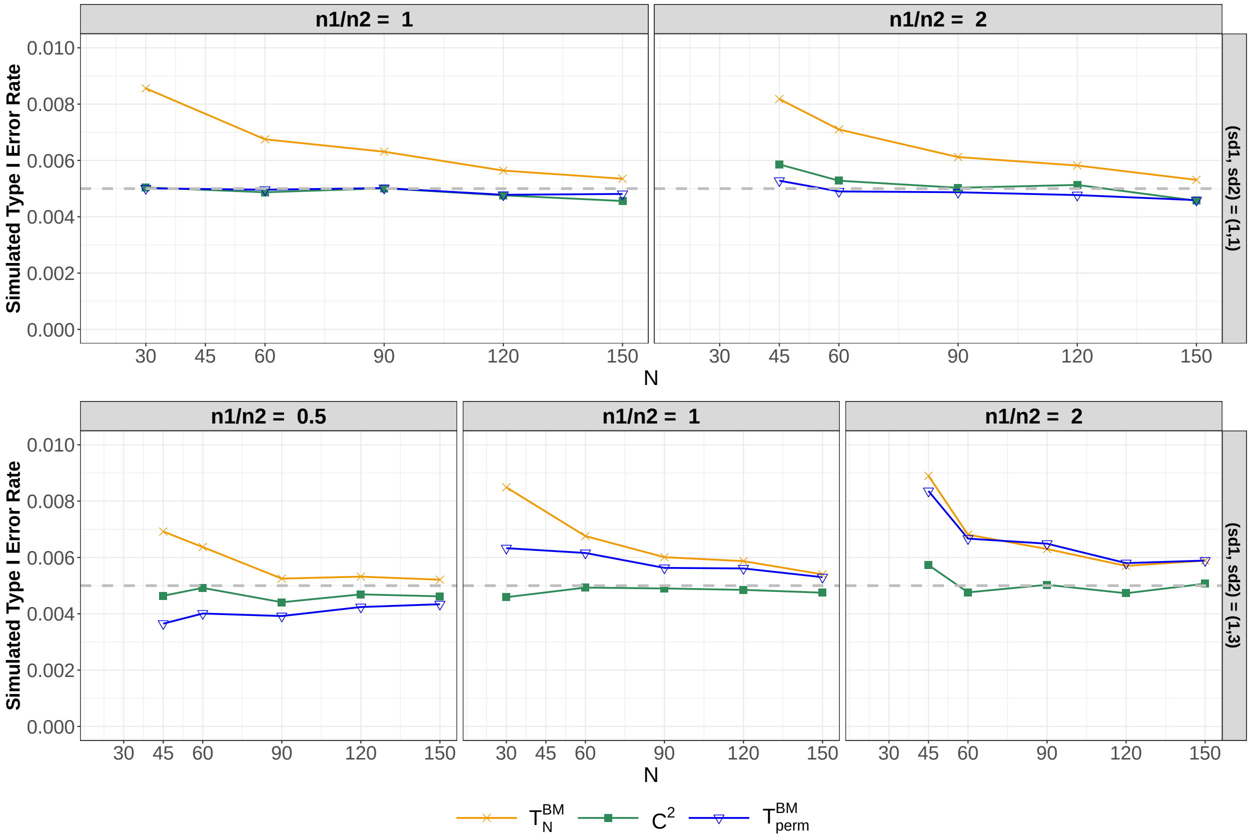

2.1.4 $\alpha = 0.001$

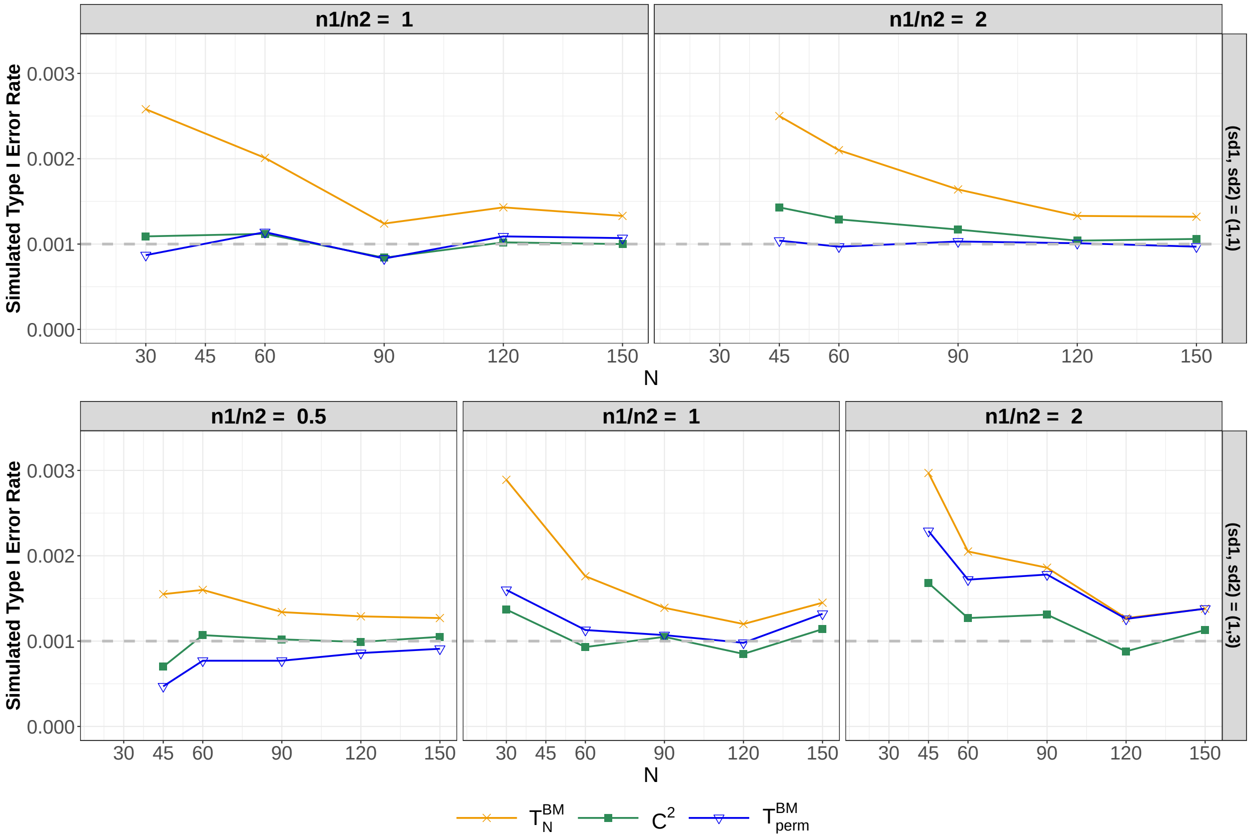

© YYYY WILEY-VCH Verlag GmbH & Co. KGaA, Weinheim

2.2 Beta Distribution

2.2.1 $\alpha = 0.05$

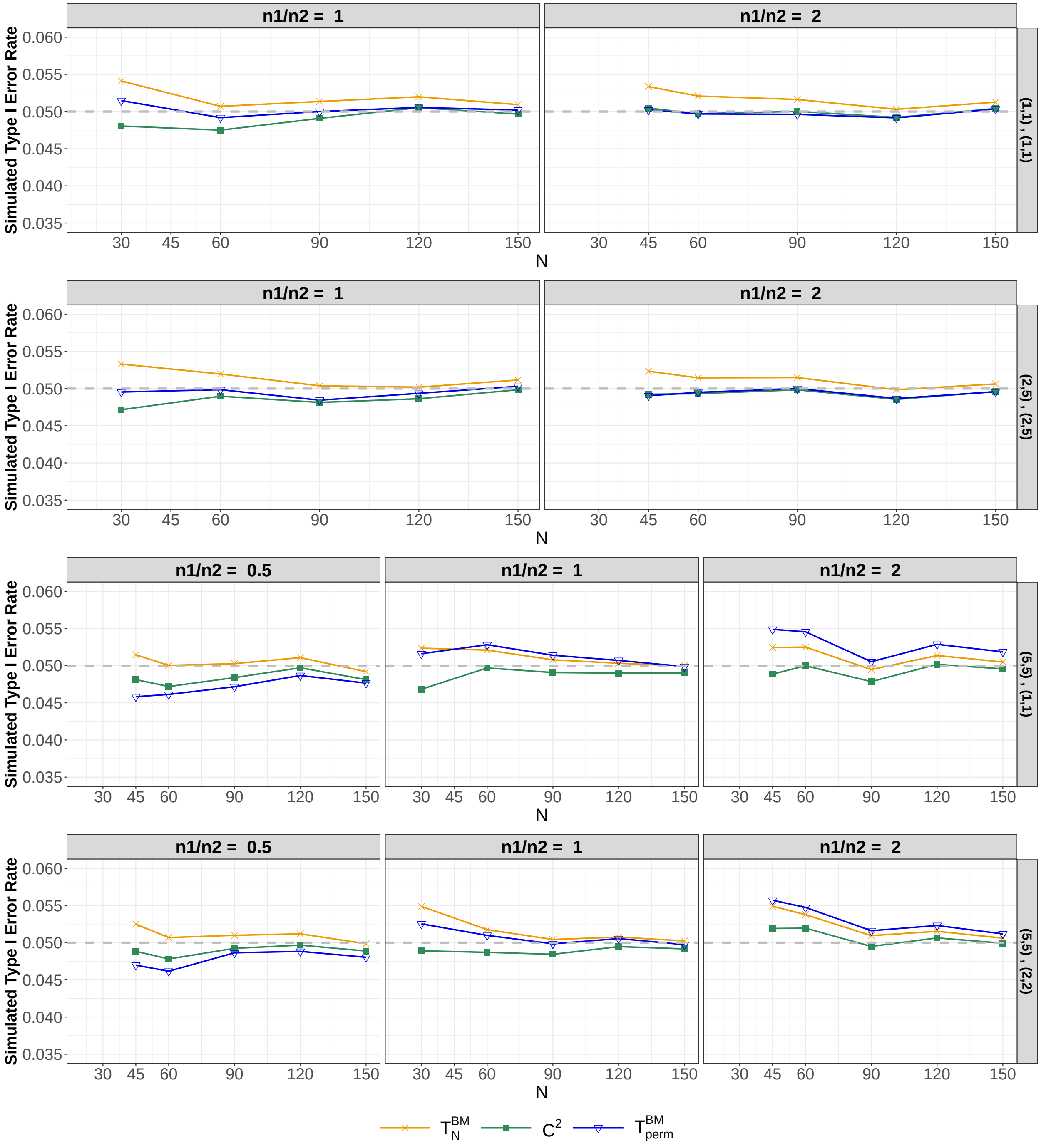

2.2.2 $\alpha = 0.01$

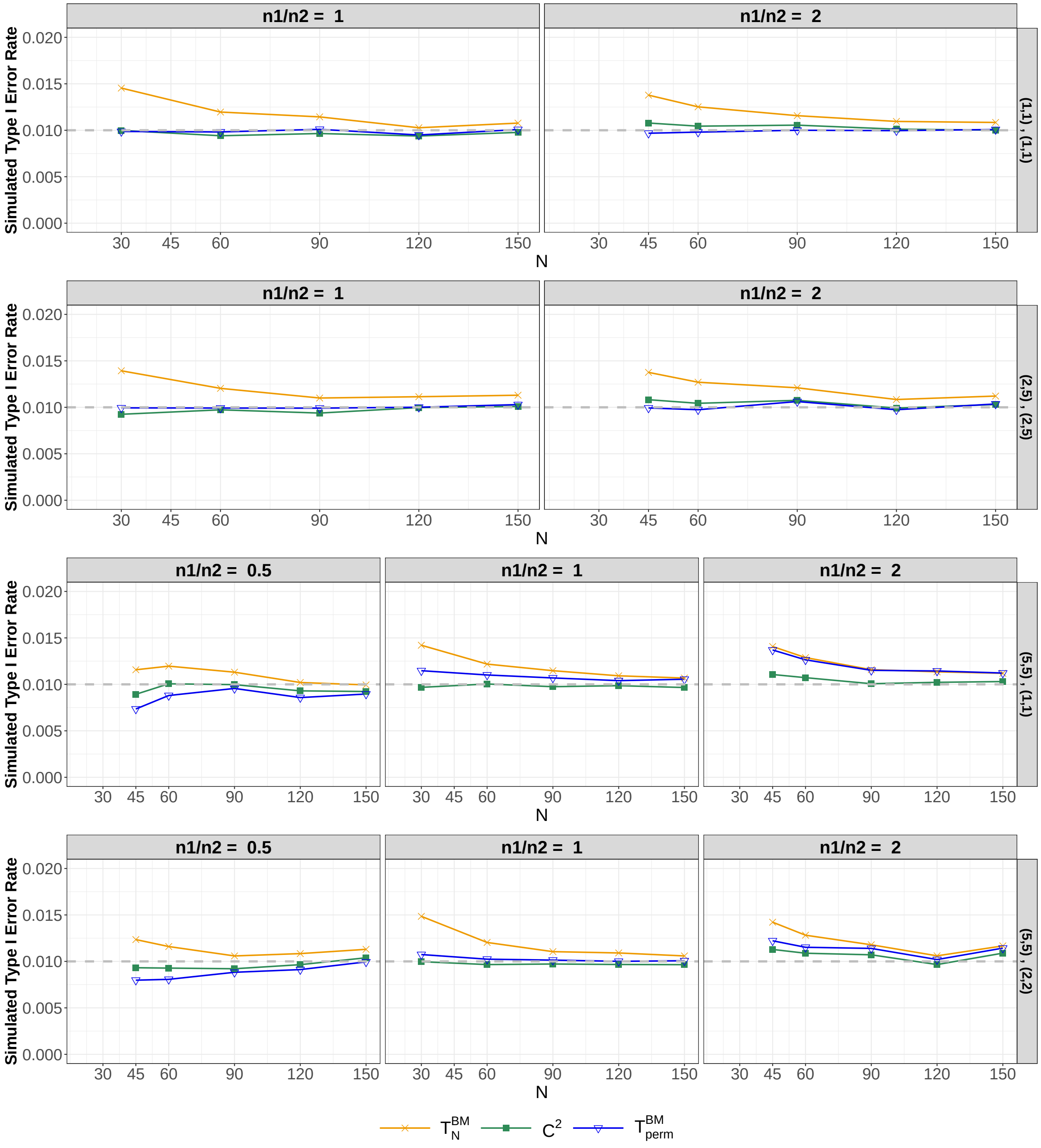

© YYYY WILEY-VCH Verlag GmbH & Co. KGaA, Weinheim

2.2.3 $\alpha = 0.005$

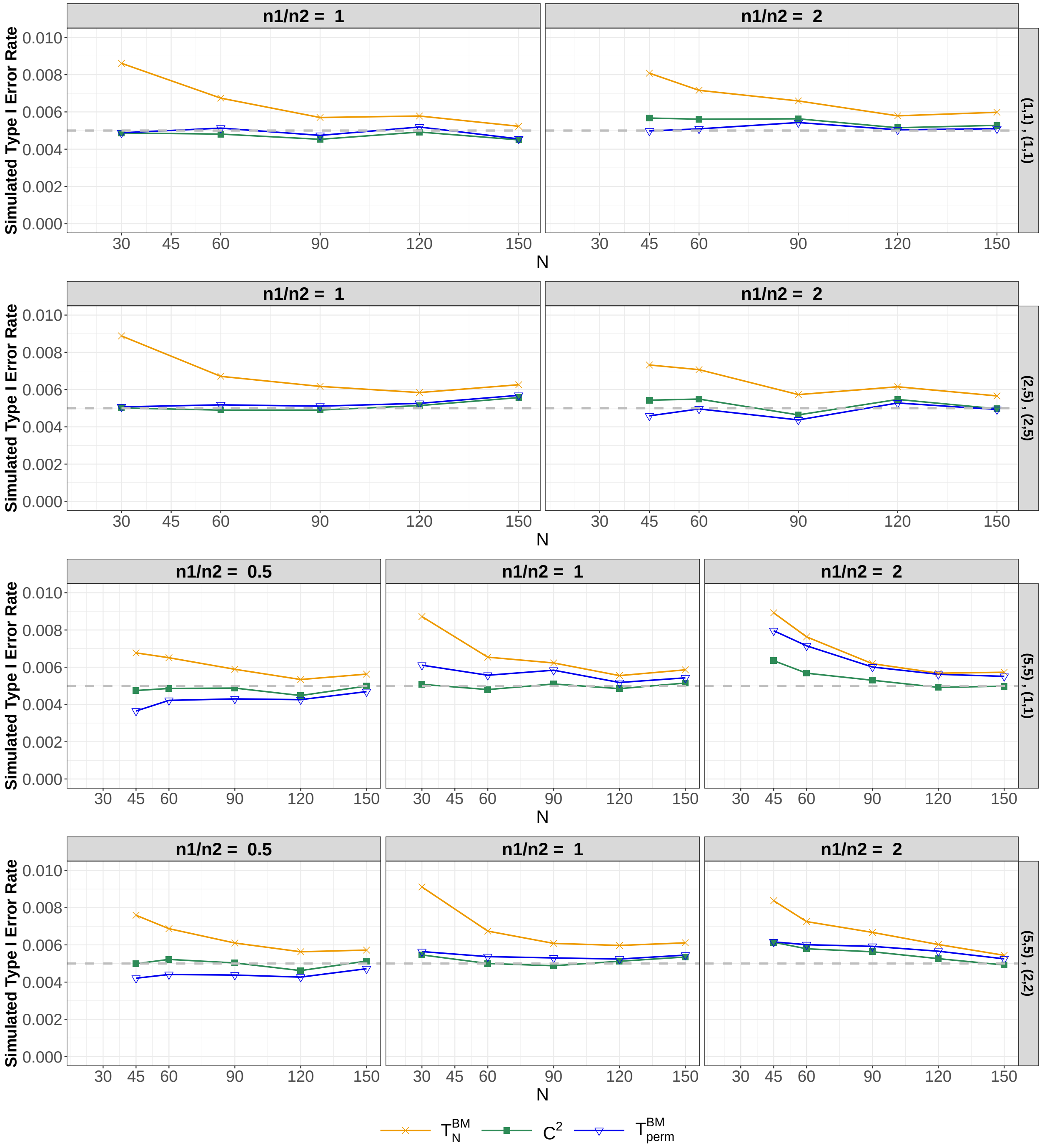

2.2.4 $\alpha = 0.001$

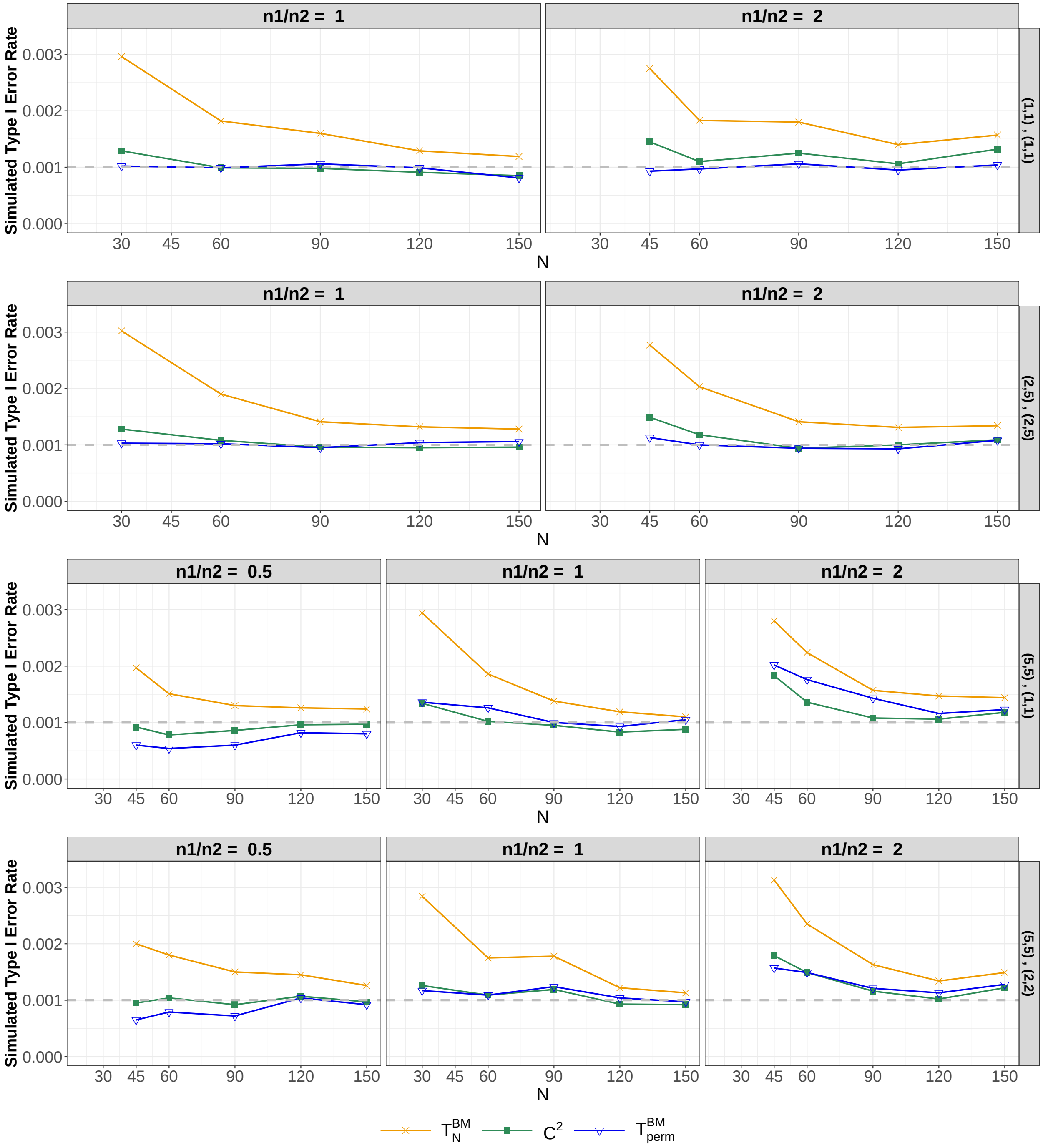

© YYYY WILEY-VCH Verlag GmbH & Co. KGaA, Weinheim

2.3 5-Point Ordered Categorical Data

2.3.1 $\alpha = 0.05$

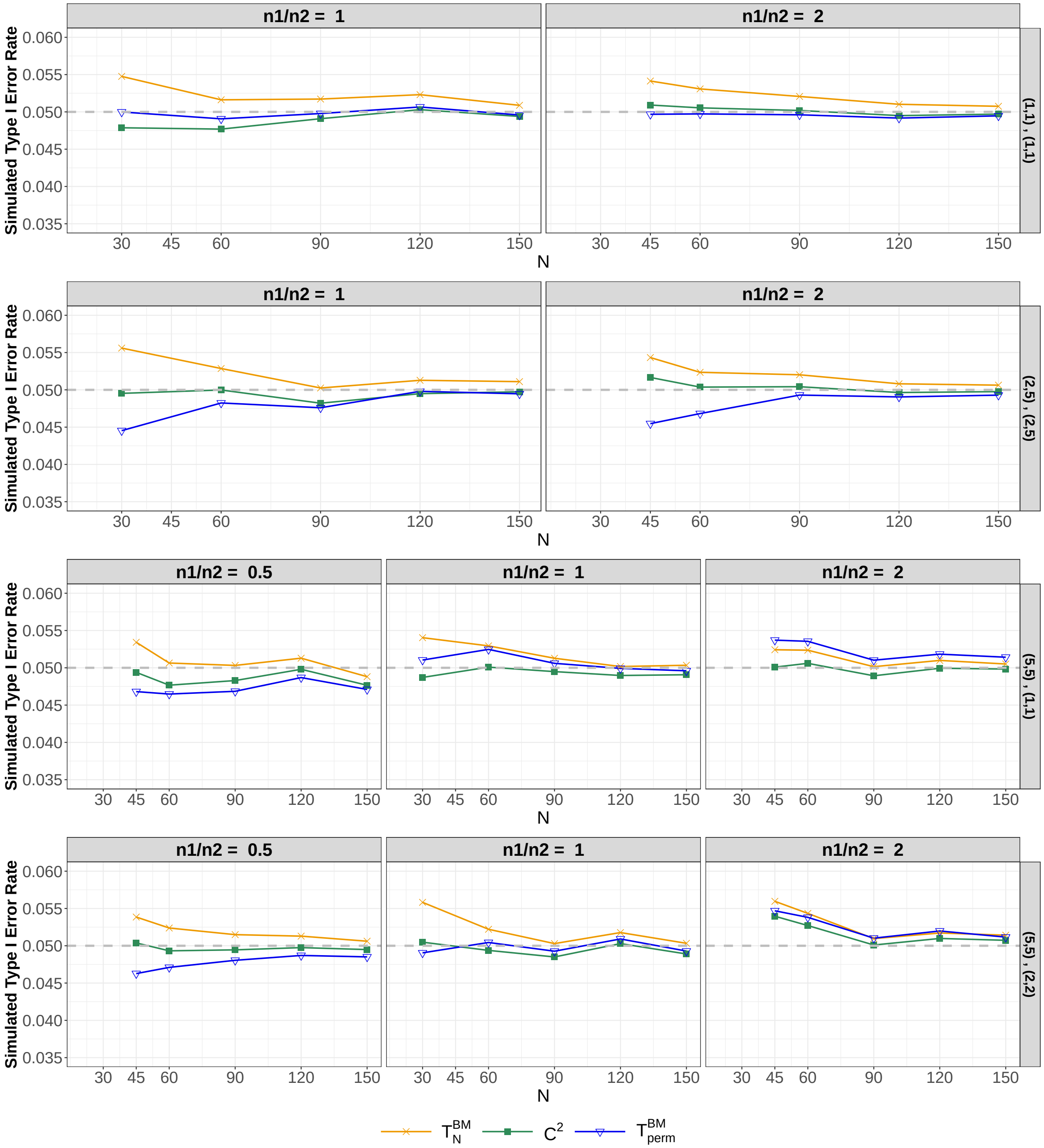

2.3.2 $\alpha = 0.01$

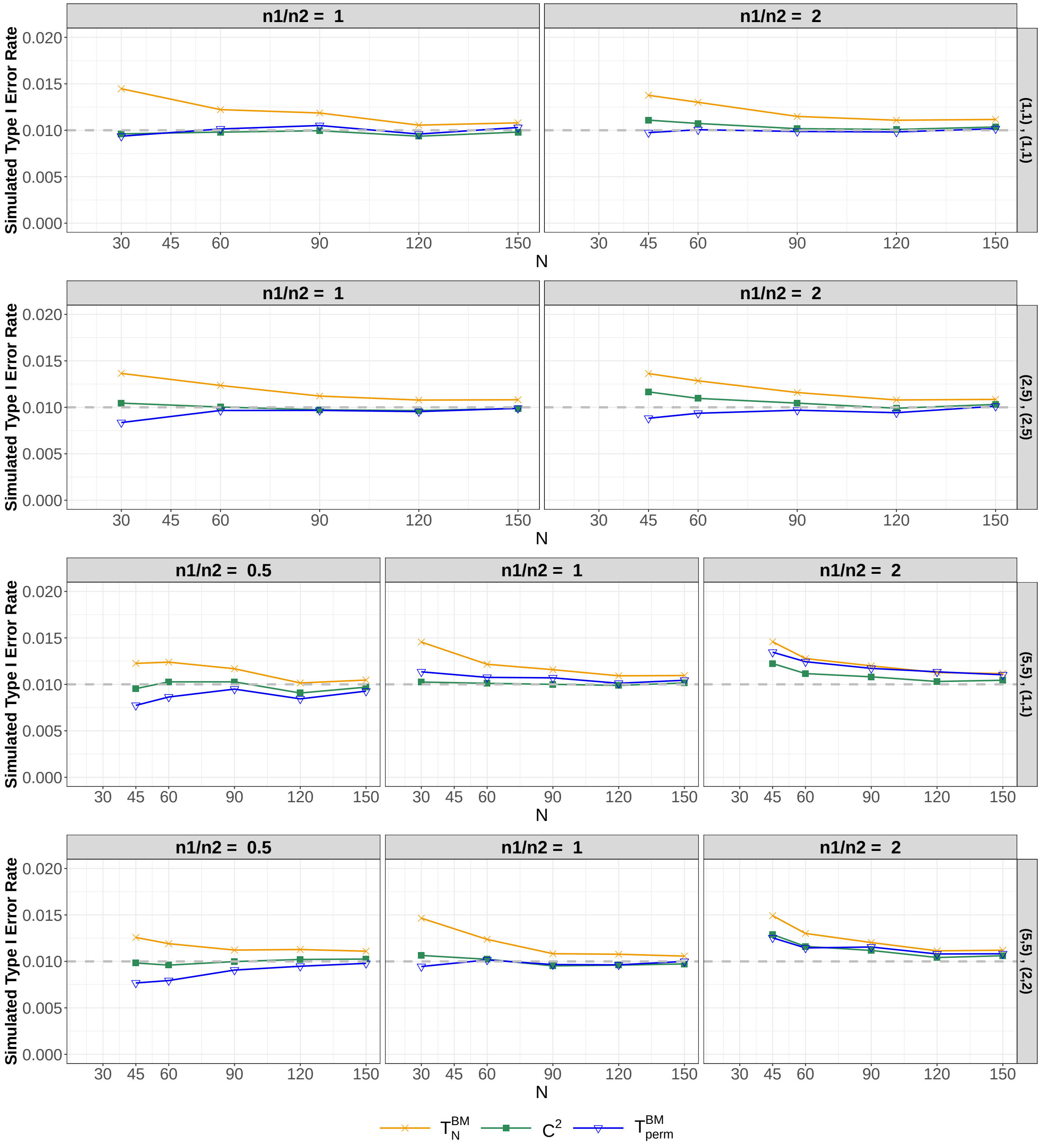

© YYYY WILEY-VCH Verlag GmbH & Co. KGaA, Weinheim

2.3.3 $\alpha = 0.005$

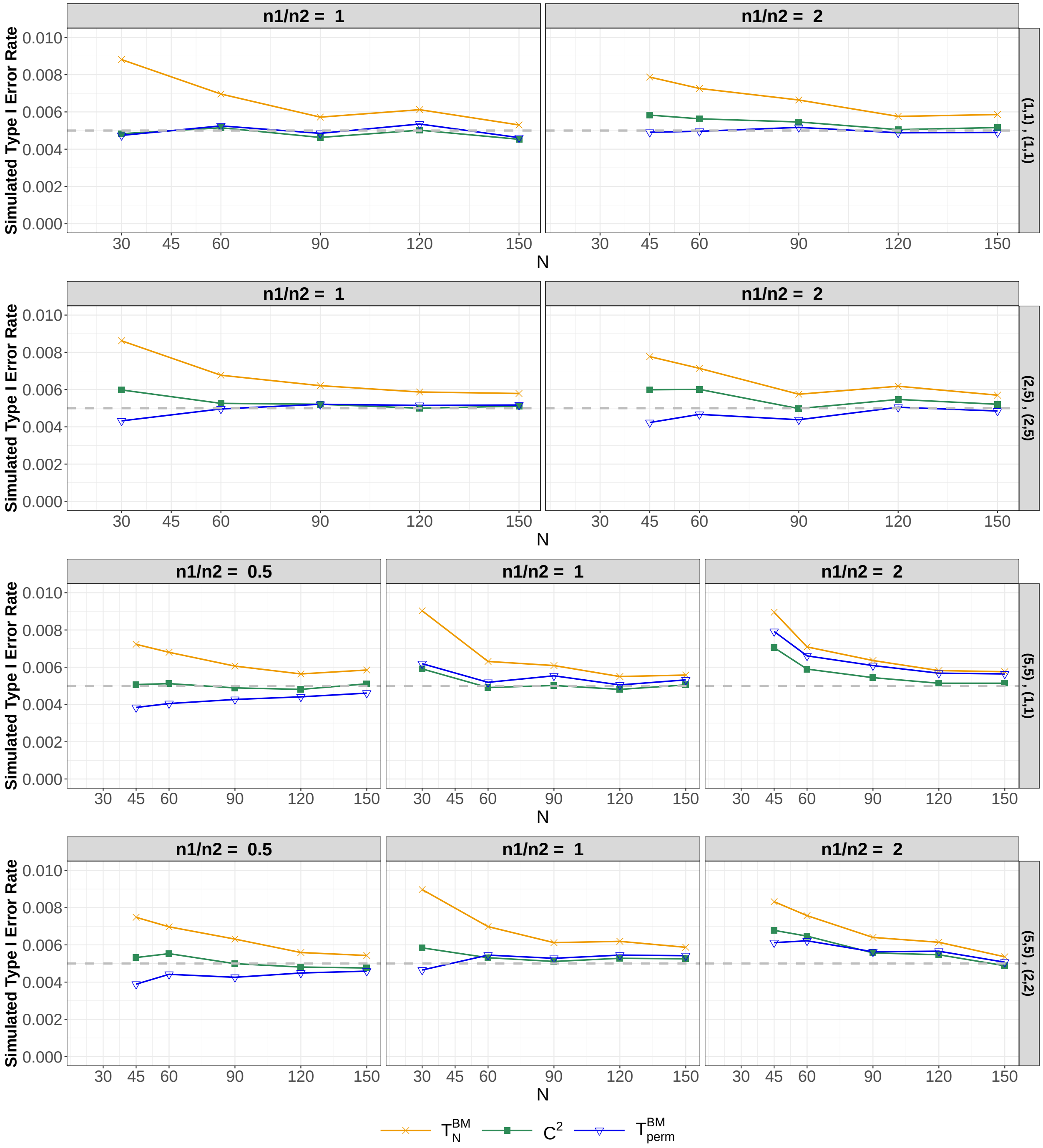

2.3.4 $\alpha = 0.001$

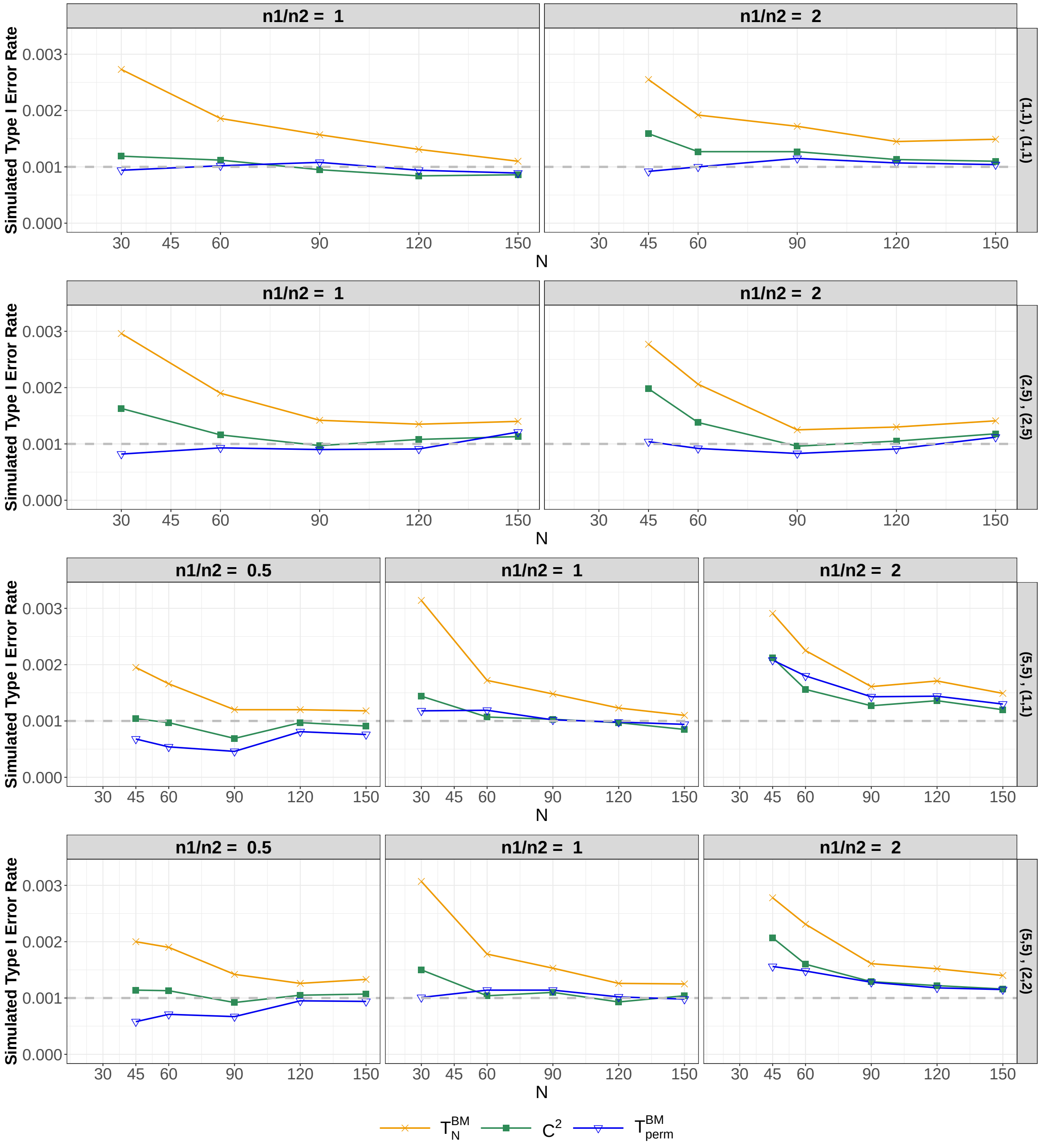

2.4 Poisson Distribution

2.4.1 $\alpha = 0.05$

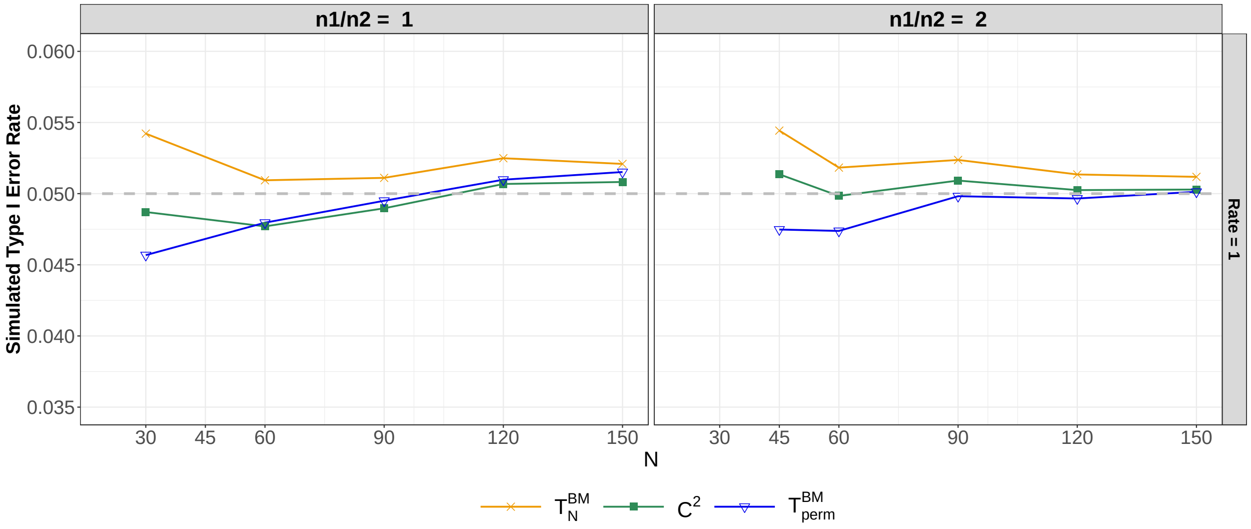

2.4.2 $\alpha = 0.01$

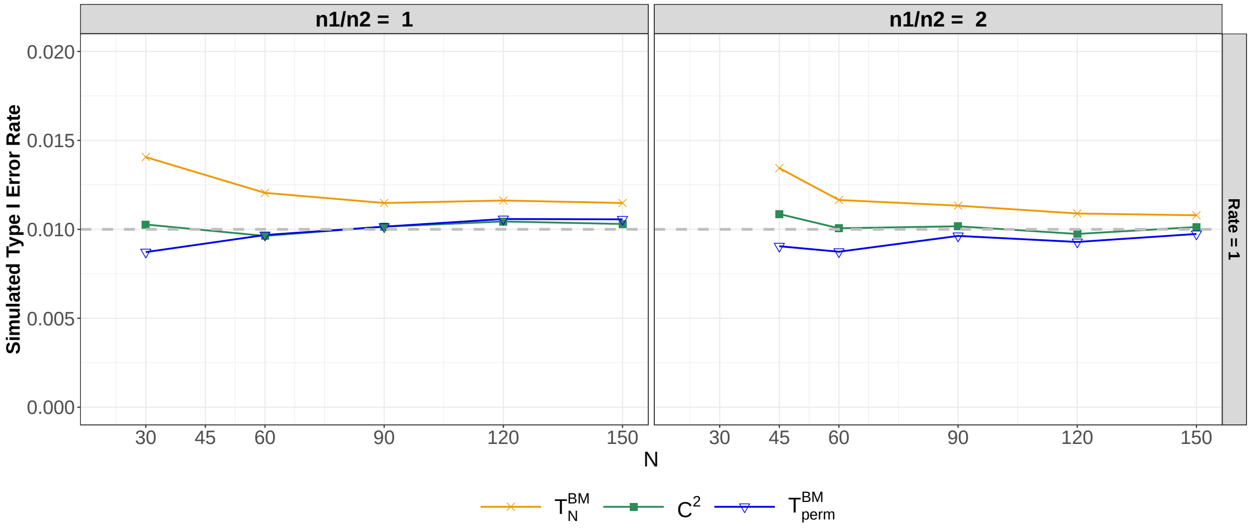

© YYYY WILEY-VCH Verlag GmbH & Co. KGaA, Weinheim

2.4.3 $\alpha = 0.005$

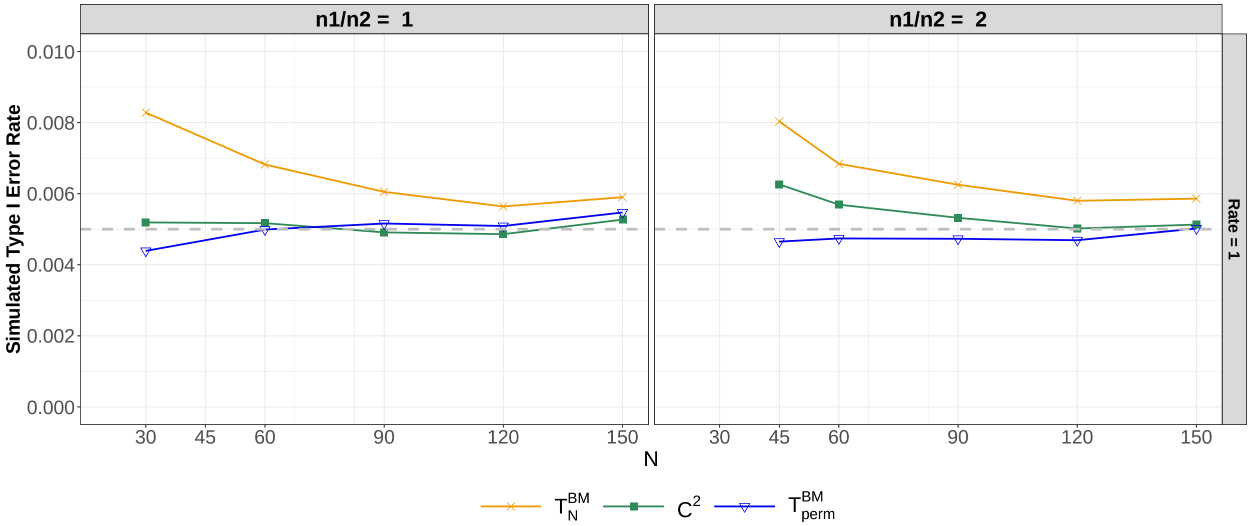

2.4.4 $\alpha = 0.001$

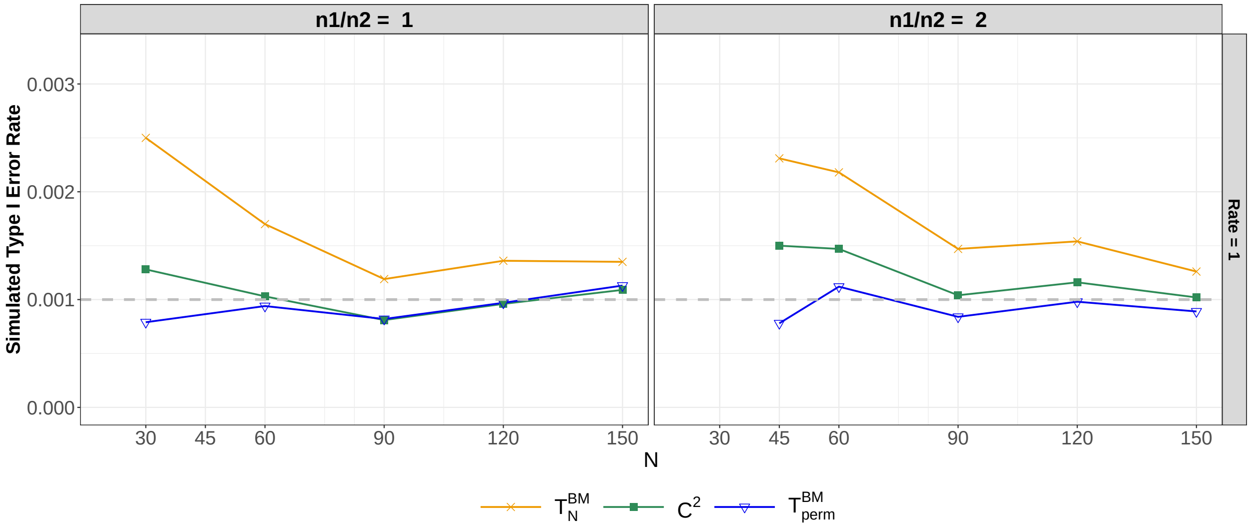

© YYYY WILEY-VCH Verlag GmbH & Co. KGaA, Weinheim

2.5 Exponential Distribution

2.5.1 $\alpha = 0.05$

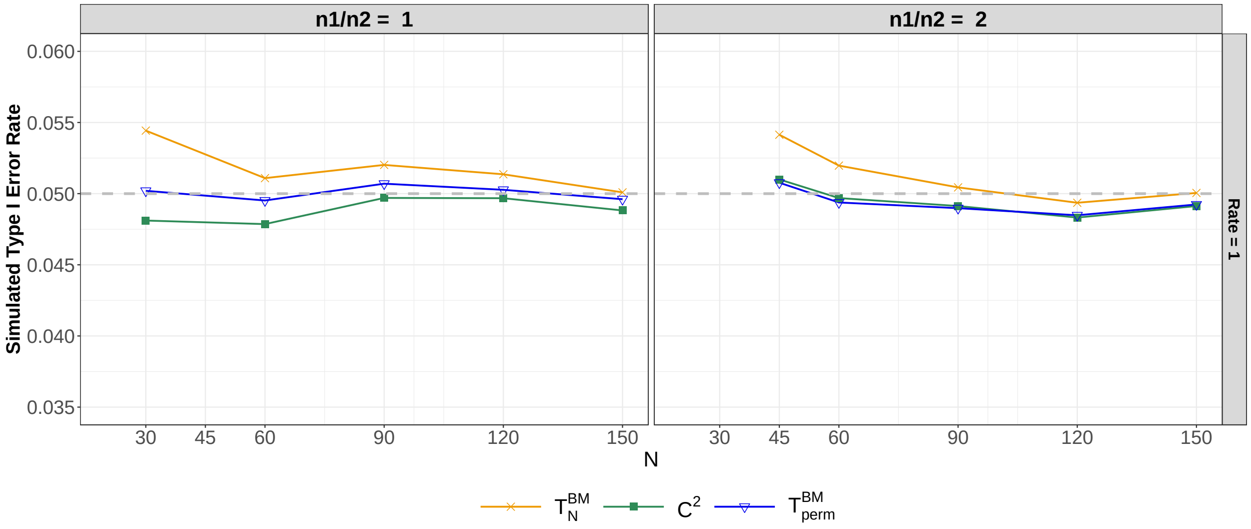

2.5.2 $\alpha = 0.01$

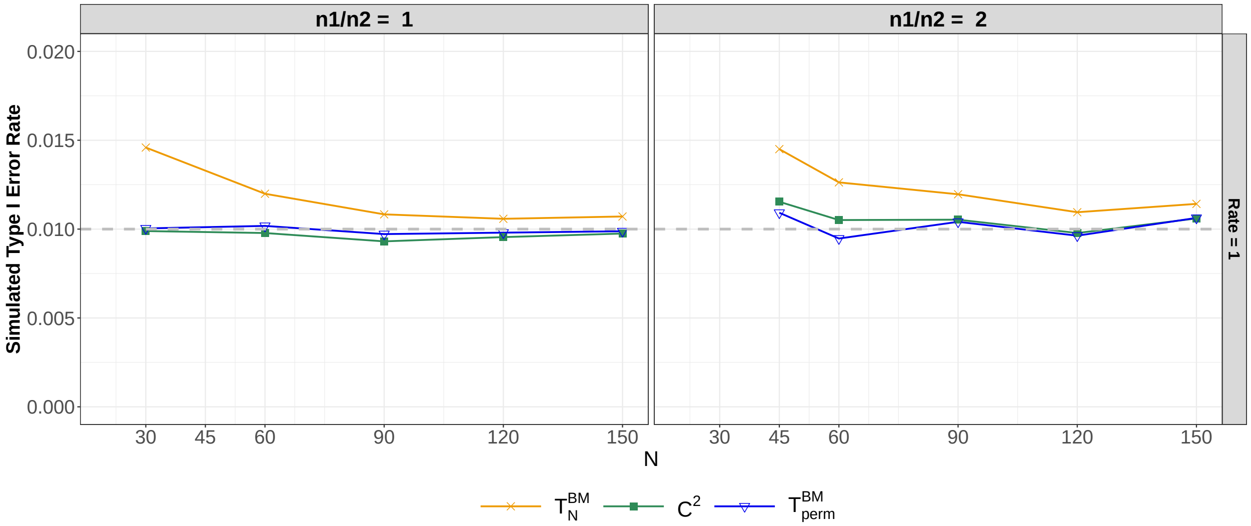

© YYYY WILEY-VCH Verlag GmbH & Co. KGaA, Weinheim

2.5.3 $\alpha = 0.005$

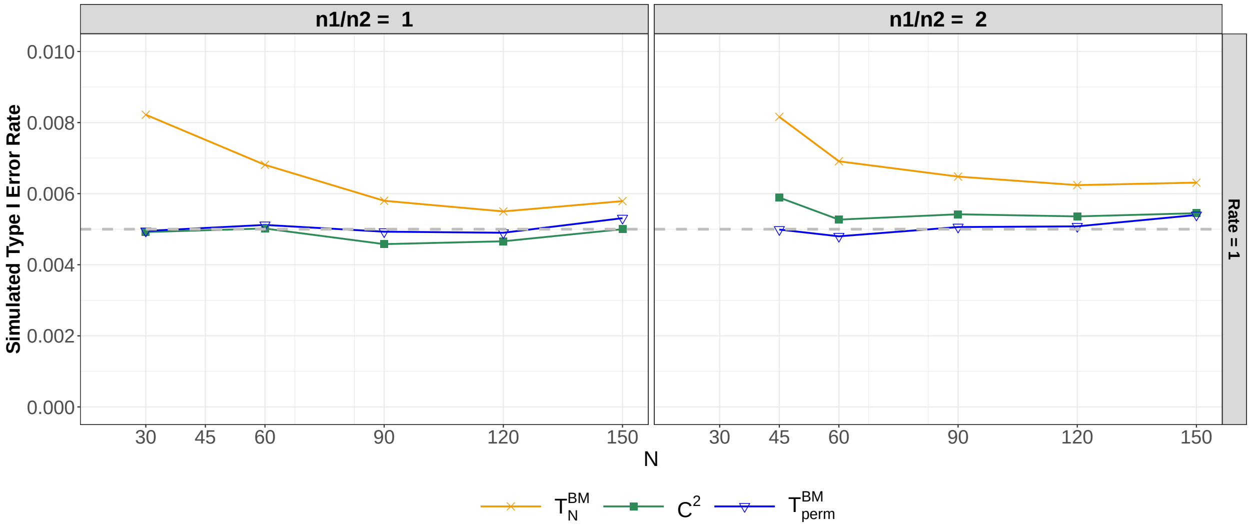

2.5.4 $\alpha = 0.001$

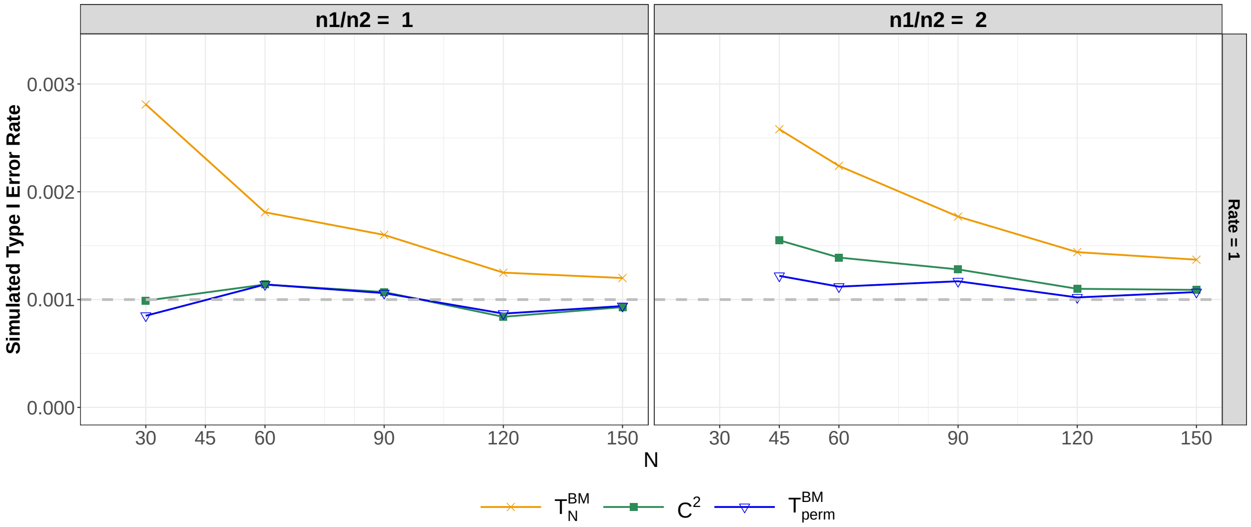

© YYYY WILEY-VCH Verlag GmbH & Co. KGaA, Weinheim

2.6 Laplace Distribution

2.6.1 $\alpha = 0.05$

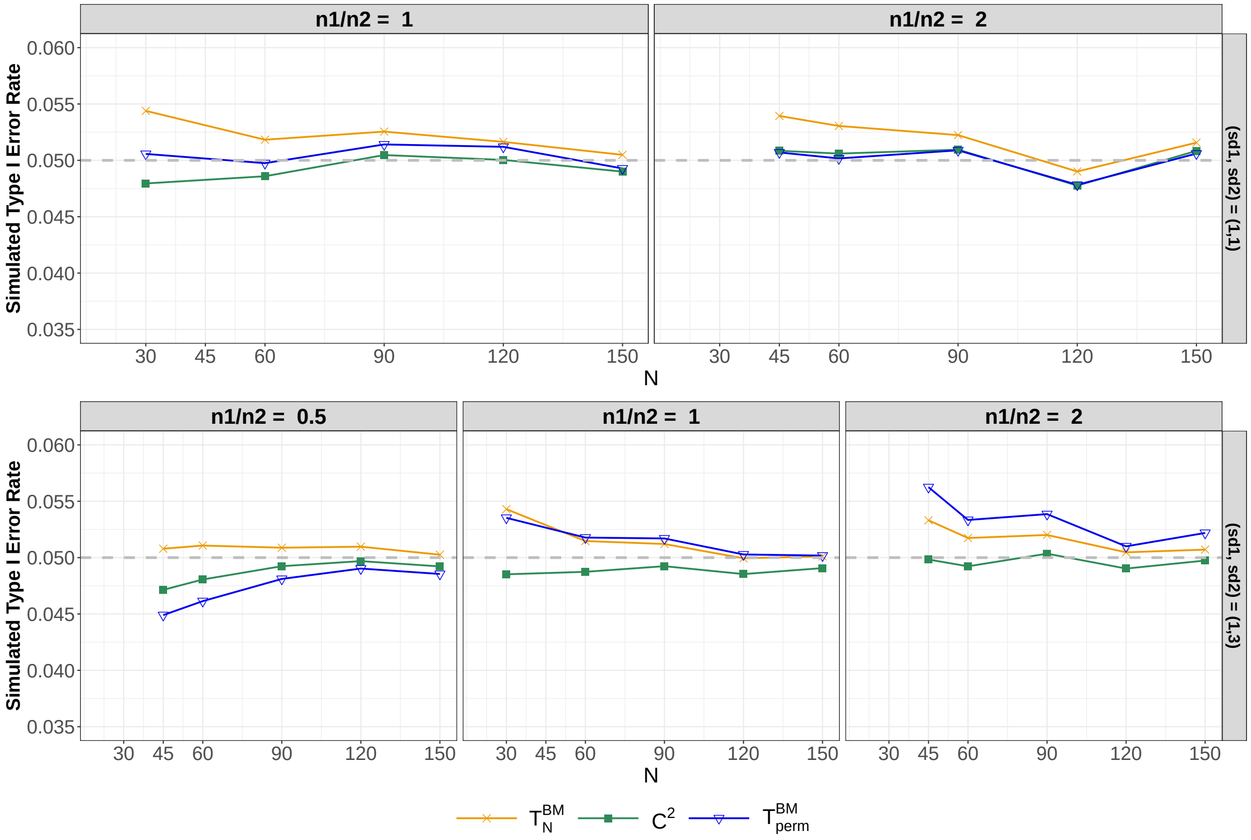

© YYYY WILEY-VCH Verlag GmbH & Co. KGaA, Weinheim

2.6.2 $\alpha = 0.01$

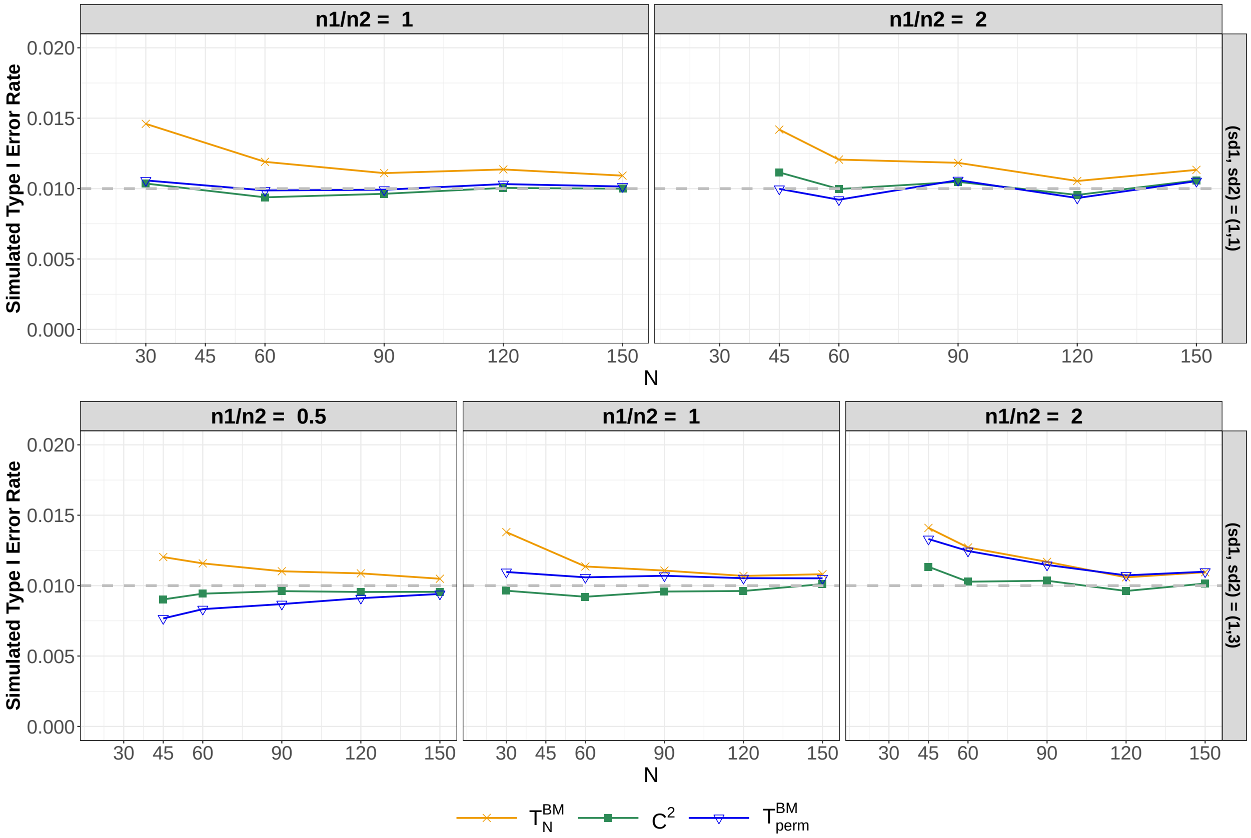

© YYYY WILEY-VCH Verlag GmbH & Co. KGaA, Weinheim

2.6.3 $\alpha = 0.005$

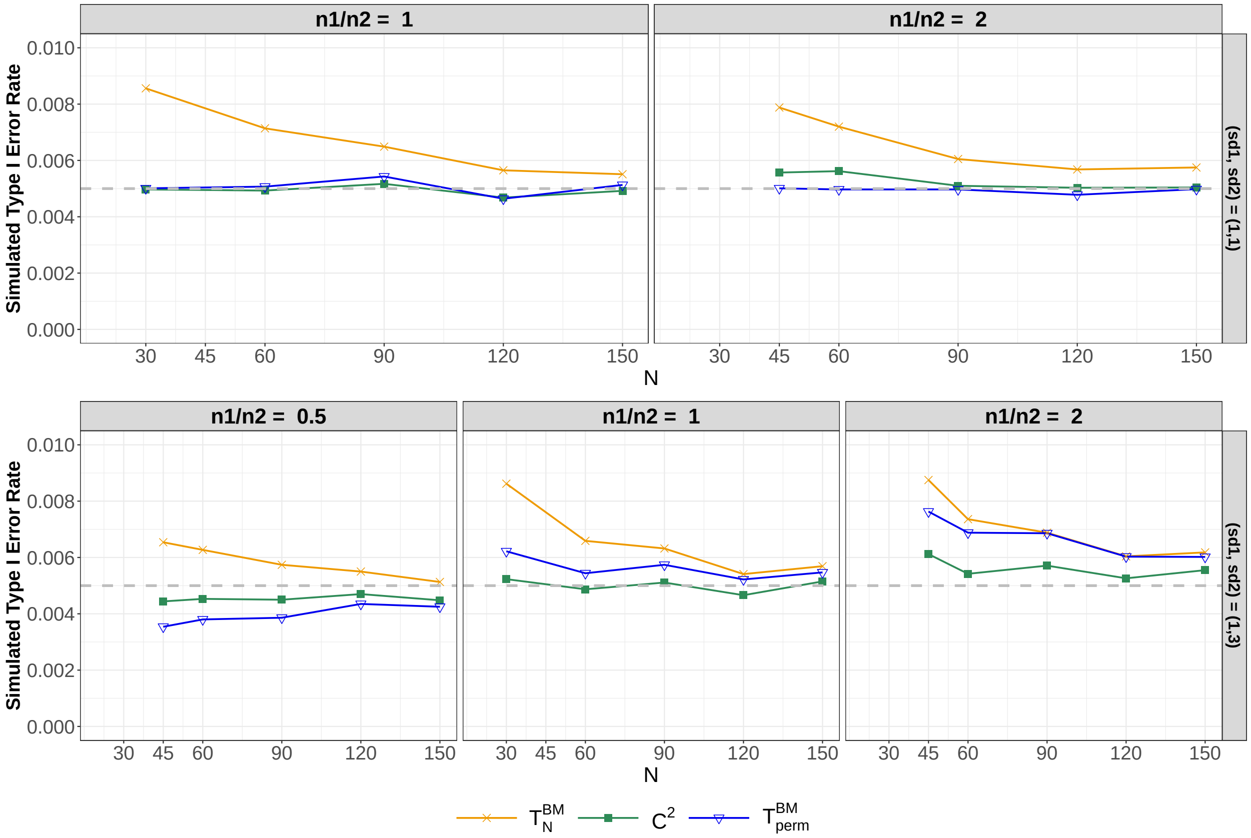

© YYYY WILEY-VCH Verlag GmbH & Co. KGaA, Weinheim

2.6.4 $\alpha = 0.001$

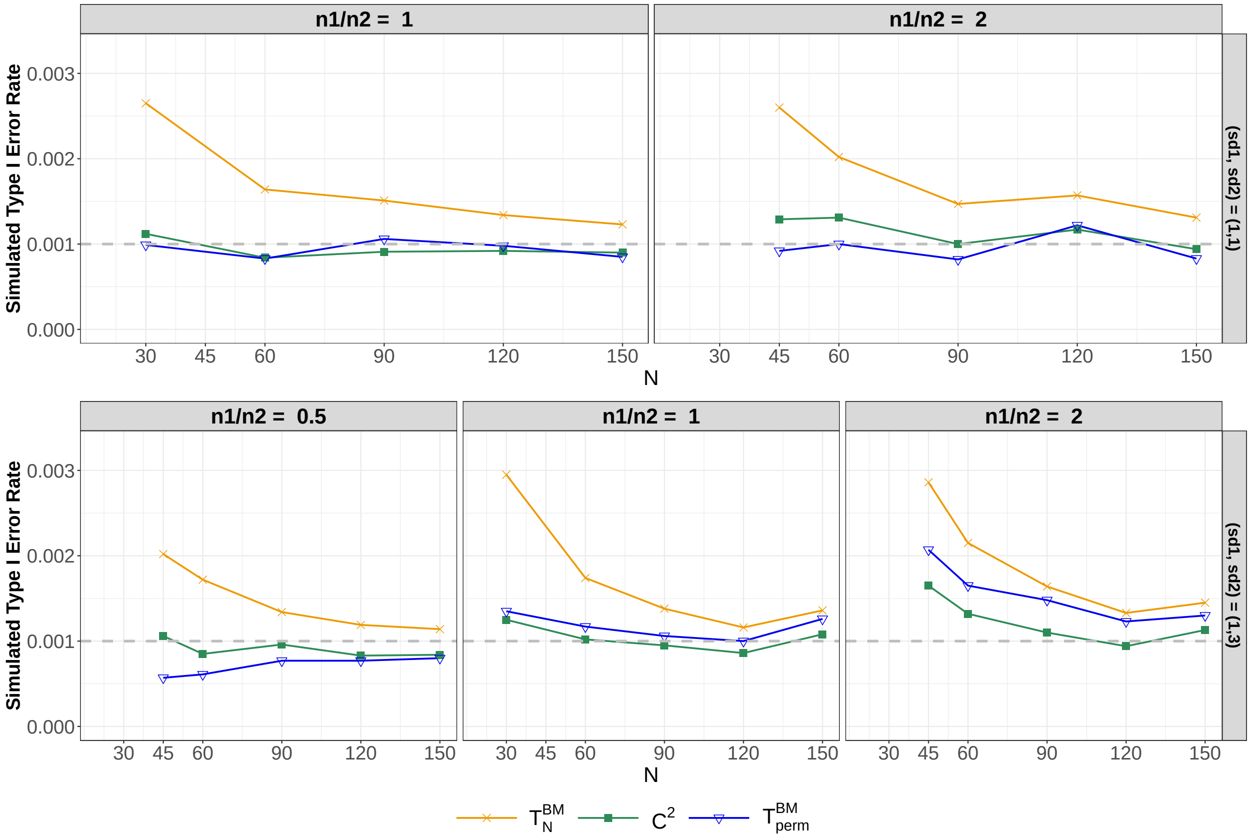

3 Power Results

3.1 Normal Distribution

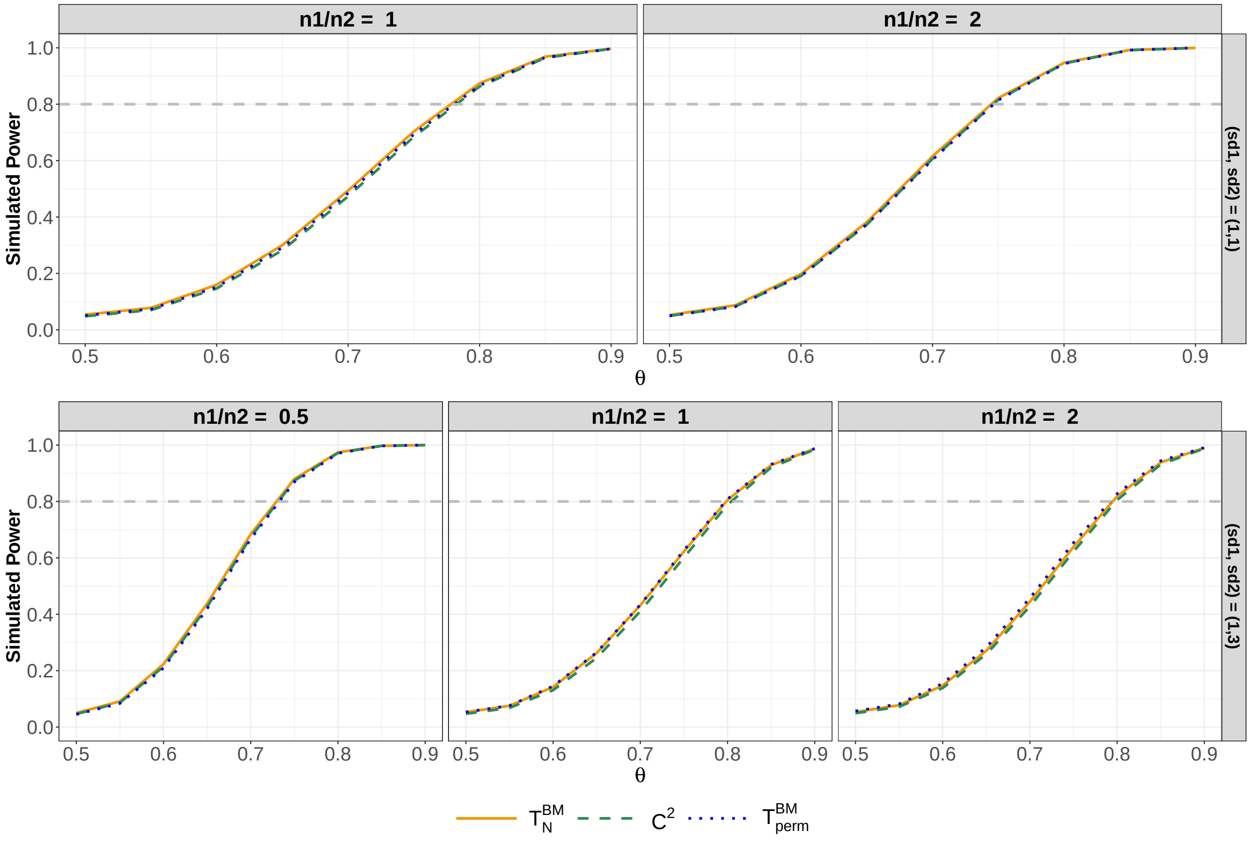

3.2 5-Point Ordered Categorical Data

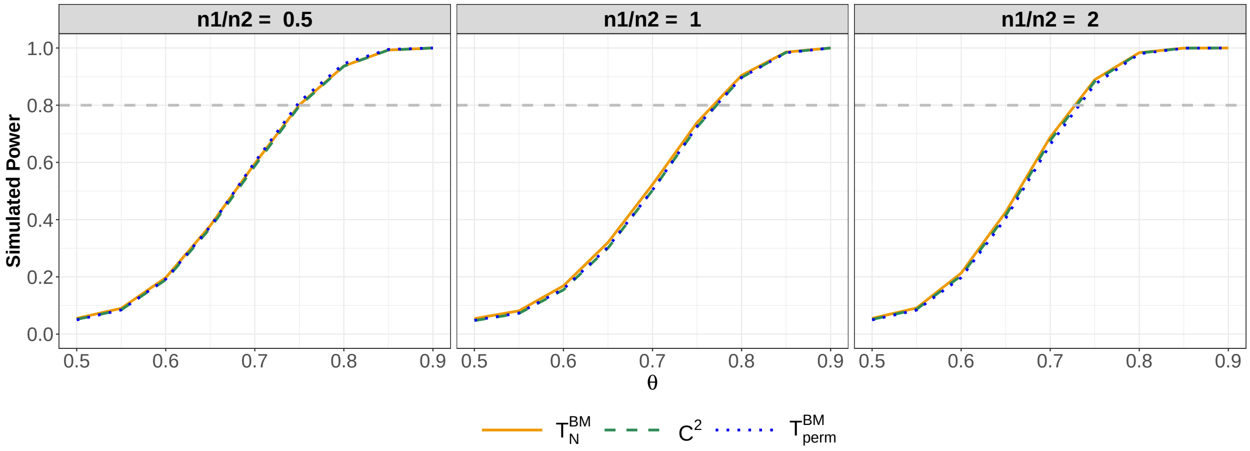

© YYYY WILEY-VCH Verlag GmbH & Co. KGaA, Weinheim

3.3 Exponential Distribution

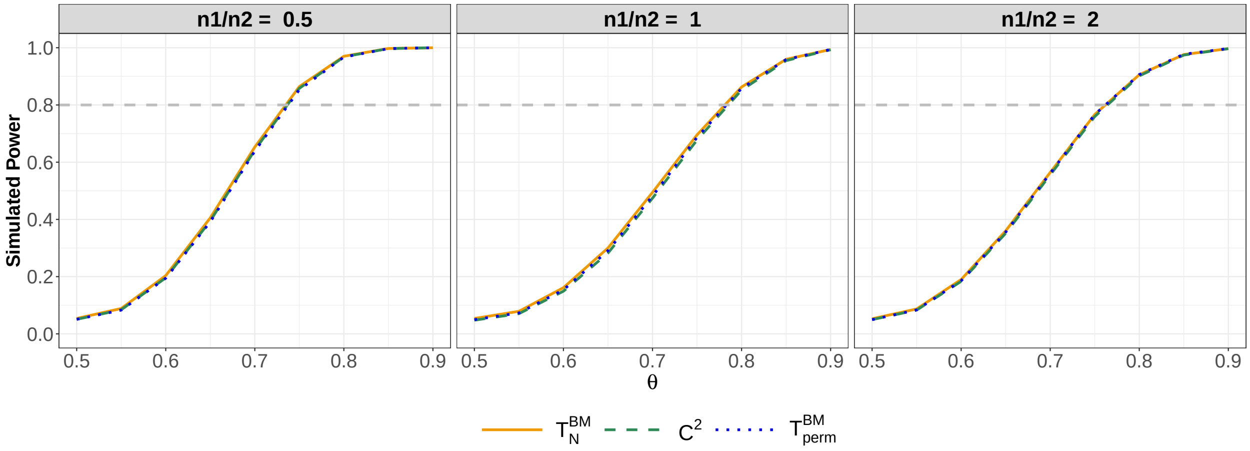

4 Coverage Probability Results

4.1 Normal Distribution

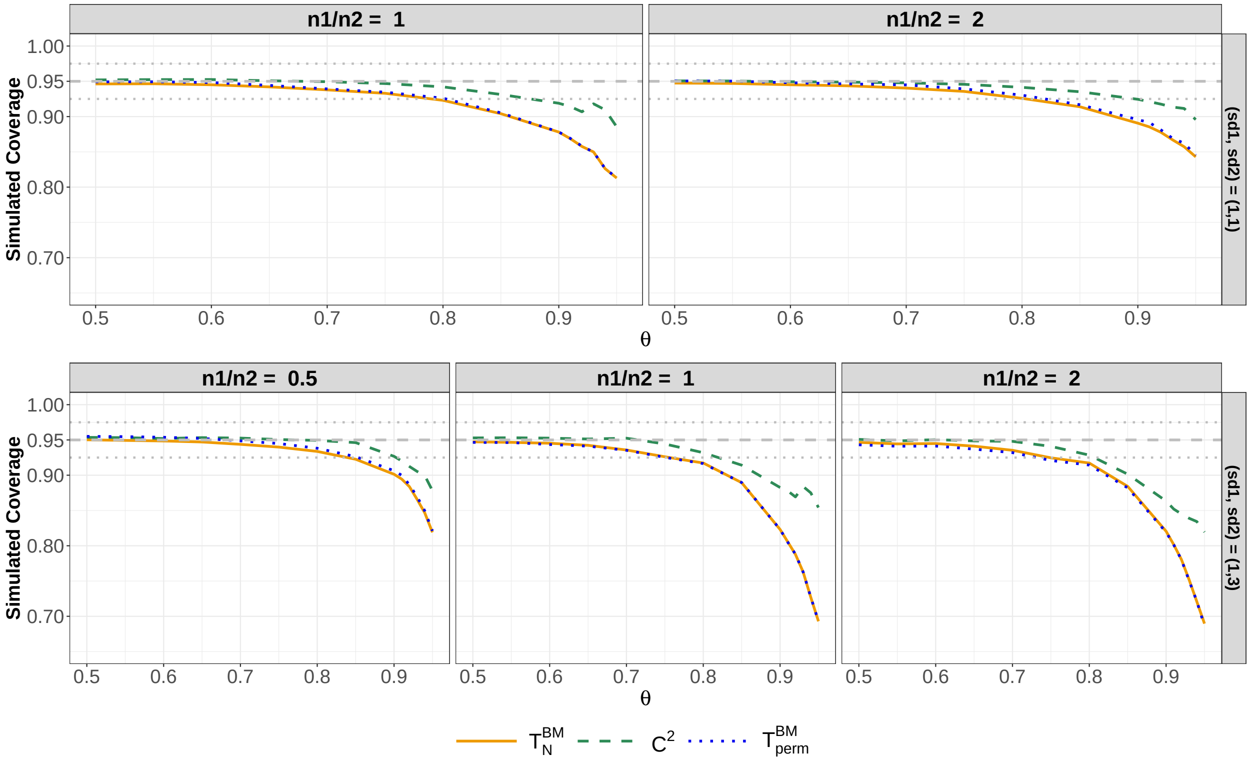

© YYYY WILEY-VCH Verlag GmbH & Co. KGaA, Weinheim
